# Synaptic Plasticity Engineering for Neural Precision, Temporal Learning, and Scalable Neuromorphic Systems

**DOI:** 10.1007/s40820-025-02028-0

**Published:** 2026-01-04

**Authors:** Zhengjun Liu, Yuxiao Fang, Qing Liu, Bobo Tian, Chun Zhao

**Affiliations:** 1 School of Advanced Technology, Xi’an Jiaotong-Liverpool University, Suzhou, 215123 People’s Republic of China; 2 Department of Electrical Engineering and Electronics, University of Liverpool, Liverpool, L69 3BX UK; 3 Chongqing Key Laboratory of Precision Optics, Chongqing Institute of East China Normal University, Chongqing, 401120 People’s Republic of China; 4 Key Laboratory of Polar Materials and Devices, Ministry of Education, Shanghai Center of Brain-Inspired Intelligent Materials and Devices, Department of Electronics, East China Normal University, Shanghai, 200241 People’s Republic of China

**Keywords:** Neuromorphic hardware, Synaptic plasticity, Edge artificial intelligence

## Abstract

Manipulating the expression of synaptic plasticity in neuromorphic devices provides essential foundations for developing intelligent, adaptive hardware systems. In recent years, advances have shifted from static emulation toward dynamic, network-oriented plasticity design, offering enhanced computational accuracy and functional relevance. This review highlights how diversified plasticity behaviors, including multilevel long-term potentiation and depression for spatial models, tunable short-term memory for temporal models, as well as wavelength-selective response, excitatory and inhibitory synergy, and adaptive threshold modulation, collectively support key tasks such as stable learning, temporal processing, and context-aware adaptation. Beyond behavioral innovations, strategies such as multifunctional single-device integration, multimodal fusion, and heterogeneous system assembly enable compact, energy-efficient, and versatile neuromorphic architectures. Recent developments at the array level further demonstrate high-performance scalability and system-level applicability. Despite notable progress, current modulation strategies remain constrained in flexibility, diversity, and large-scale coordination. Future research should focus on enriching the behavioral repertoire of plasticity, advancing cross-modal convergence, and improving array-level uniformity, paving the way toward deployable, high-efficiency neuromorphic intelligence.

## Highlights

- This review provides an in-depth discussion of computing-unit optimization through synaptic plasticity engineering, enabling precise weight modulation in spatial models and effective temporal information processing in dynamic neural networks.
- It delves into algorithmic advancement through plasticity modulation, improving accuracy, stability, and convergence in neuromorphic computing models.
- It explores resource-efficient neuromorphic architectures, integrating multifunctional devices, multimodal fusion, and heterogeneous arrays for scalable, low-power, and generalizable intelligent systems.

## Graphical abstract

**Figure:**
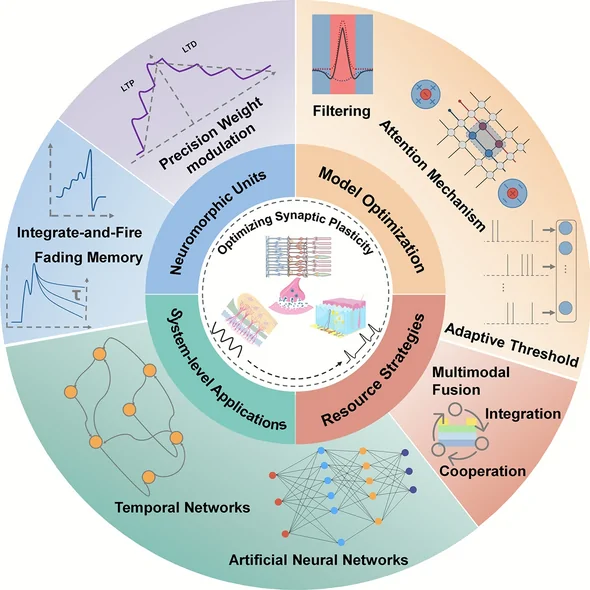

## Introduction

Driven by the rapid evolution of brain-inspired intelligence and the proliferation of ubiquitous intelligent terminals, neuromorphic devices and networks are increasingly recognized as the foundational hardware underpinning next-generation artificial intelligence (AI) systems [1]. Neuromorphic engineering, situated at the intersection of neuroscience, materials science, electronics, and AI, aims to emulate the multiscale information processing and adaptive learning capabilities intrinsic to biological neural systems [2–5]. By overcoming the fundamental limitations of traditional von Neumann architectures, such as high energy consumption, the separation of memory and computation, and data transfer bottlenecks, neuromorphic hardware aspires to integrate sensing, memory, and computing within unified physical substrates [6–8]. This vision unlocks new possibilities for building AI systems that are energy-efficient and adaptive, as well as deployable in resource-constrained, edge, and extreme-environment scenarios [9–11].

Two functionally differentiated but equally critical research directions have emerged to meet the unique requirements of static and dynamic neural architectures [12, 13]. For spatial-domain, weight-driven networks such as convolutional neural networks (CNNs) and multilayer perceptrons (MLPs), the emphasis is placed on achieving precise, stable, and symmetric modulation of synaptic weights [14]. To support accurate and scalable training and inference in neuromorphic networks, long-term memory (LTM) synaptic devices must exhibit high-resolution analog weight states, minimal nonlinearity, excellent long-term retention, and strong environmental drift resistance [15]. Substantial progress has been made in this area, as demonstrated by advances in multilayer memristors, ferroelectric synapses, and two-dimensional heterostructure devices, which collectively offer excellent linearity, multistate modulation, and symmetry [16–18]. In parallel, solid-state ionic systems have demonstrated outstanding endurance and drift resistance, further supporting the feasibility of deploying these devices in large-scale neuromorphic arrays [19, 20].

On the other hand, temporally dynamic architectures such as reservoir computing (RC) and spiking neural networks (SNNs) require devices capable of emulating transient synaptic behaviors that support time-domain information processing [21]. Short-term memory (STM), paired-pulse facilitation (PPF), and spike-timing-dependent plasticity (STDP) are critical for encoding spatiotemporal correlations and enabling event-driven learning [15, 22, 23]. To address these requirements, researchers have developed neuromorphic devices that emulate such behaviors through a variety of mechanisms. Notable advances include fully quantum dot optoelectronic memristors, photon-avalanche nanocrystal-based synapses, and hybrid organic transistor systems [24–26]. These devices offer tunable memory windows, nonlinear temporal dynamics, and dynamic threshold modulation, features that render them highly suitable for real-time pattern recognition, sequence prediction, and low-power adaptive computing [27].

**Fig. 1:**
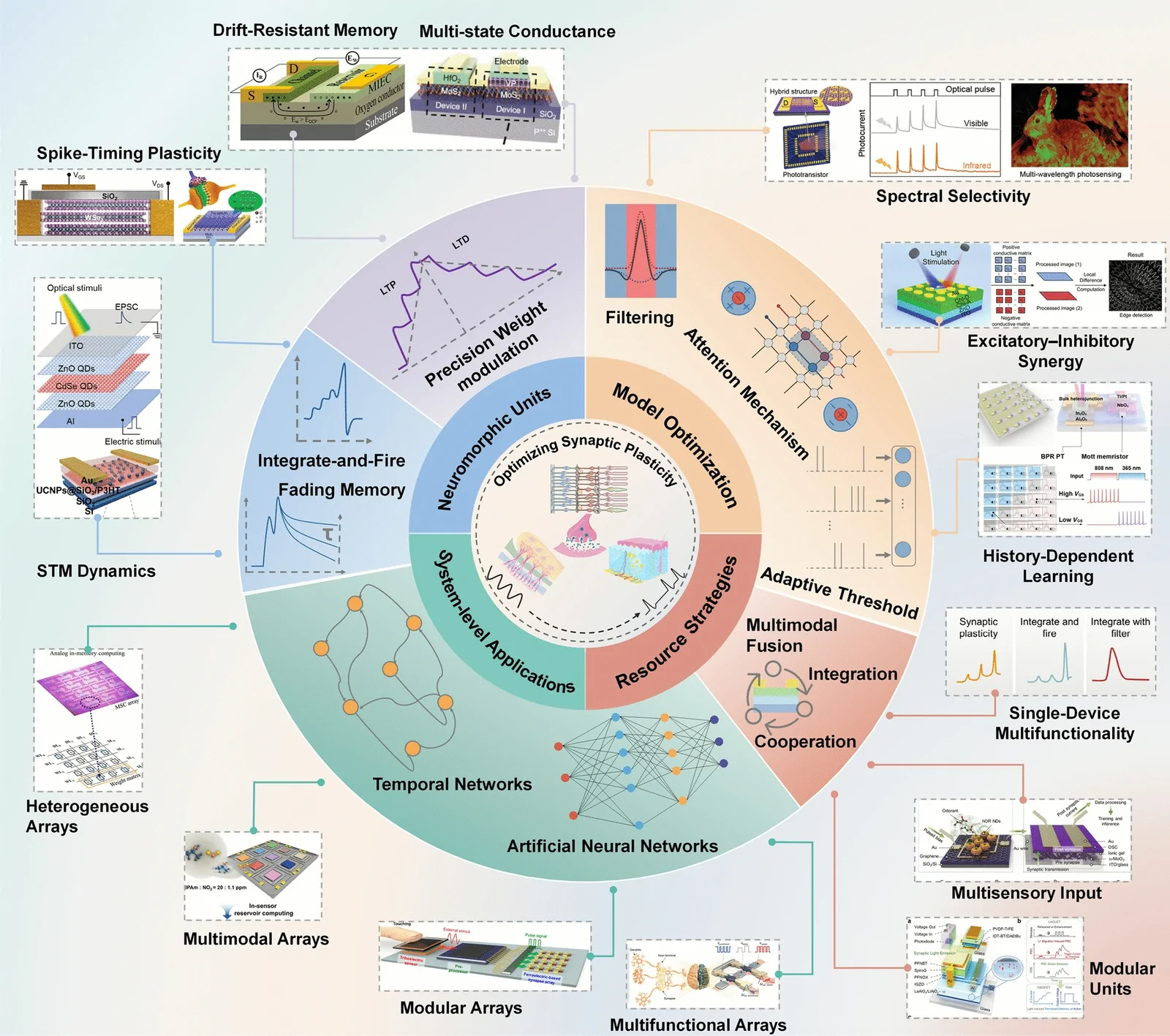
Overview of this review. Neuromorphic Units. Reproduced with permission [35]. Copyright 2023, The Authors. Advanced Science published by Wiley‐VCH GmbH. Reproduced with permission [36]. Copyright 2024, Wiley–VCH GmbH. Reproduced with permission [37]. Copyright 2025, Wiley‐VCH GmbH. Reproduced with permission [38]. Copyright 2024, The Author(s). Advanced Materials published by Wiley‐VCH GmbH. Reproduced with permission [39]. Copyright 2024, Wiley‐VCH GmbH. Reproduced with permission [40]. Copyright 2021, Wiley‐VCH GmbH. Model Optimization. Reproduced with permission [41]. Copyright 2024, Wiley‐VCH GmbH. Reproduced with permission [42]. Copyright 2024, Wiley‐VCH GmbH. Reproduced with permission [43]. Copyright 2025, The American Association for the Advancement of Science. Resource Strategies. Reproduced with permission [44]. Copyright 2024, The American Association for the Advancement of Science. Reproduced with permission [45]. Copyright 2024, American Chemical Society. Reproduced with permission [46]. Copyright 2024, The Authors. Advanced Materials published by Wiley‐VCH GmbH. System Integration. Reproduced with permission [47]. Copyright 2024, The Author(s). Advanced Materials Technologies published by Wiley–VCH GmbH. Reproduced with permission [48]. Copyright 2024, The American Association for the Advancement of Science. Reproduced with permission [49]. Copyright 2023, American Chemical Society

Earlier research mainly emphasized synaptic behaviors at the single-device level. However, the translation of these advances into network- and system-level benefits has been comparatively underexplored. In contrast, this review emphasizes how diverse synaptic plasticity mechanisms can directly enhance neural network algorithm optimization, resource efficiency, and generalization (Fig. 1). Recent advances in wavelength-selective plasticity, excitatory and inhibitory synergy, and dynamically tunable metaplasticity demonstrate significant algorithmic implications [28–34]. Wavelength-selective synapses facilitate targeted spectral perception and intrinsic noise filtering, streamlining neural network algorithms by reducing preprocessing complexity and enhancing robustness under noisy, real-world conditions [35]. Excitatory and inhibitory cooperative synapses, mirroring biological receptive fields, realize spatial attention mechanisms at the hardware level, prioritizing critical inputs and suppressing irrelevant signals, thus reducing computational overhead and improving recognition accuracy even under constrained computational budgets [36]. Additionally, dynamically adjustable threshold plasticity and metaplasticity enable neural networks to modulate learning sensitivity and response thresholds based on environmental variability and historical activity, accelerating model convergence, reducing required training epochs, and enhancing adaptability to uncertain conditions [37].

In parallel with the advancement of higher-order synaptic plasticity mechanisms, recent developments in multimodal integration, single-device multifunctionality, and heterogeneous component co-design have established powerful strategies for constructing energy-efficient, resource-compact neuromorphic systems [50–52]. Multimodal synaptic devices capable of responding to diverse stimuli such as light, pressure, gas, or humidity facilitate device-level information fusion and reduce data transfer overhead, supporting robust and low-latency perception in dynamic environments [53]. Multifunctional synapses emphasize internal behavioral richness, allowing single devices to concurrently exhibit various plasticity modes, significantly enhancing learning flexibility, reducing peripheral circuitry, and enabling task-adaptive learning under tight hardware constraints [54]. Complementing these approaches, heterogeneous integration architectures amalgamate sensory transducers, memory units, logic elements, and actuators into unified neuromorphic modules, supporting real-time closed-loop operations. Compared to monolithic systems, these heterogeneous platforms offer enhanced versatility, scalability, and task-specific configurability, essential for intelligent operation in power-constrained and bandwidth-limited environments [55].

**Fig. 2:**
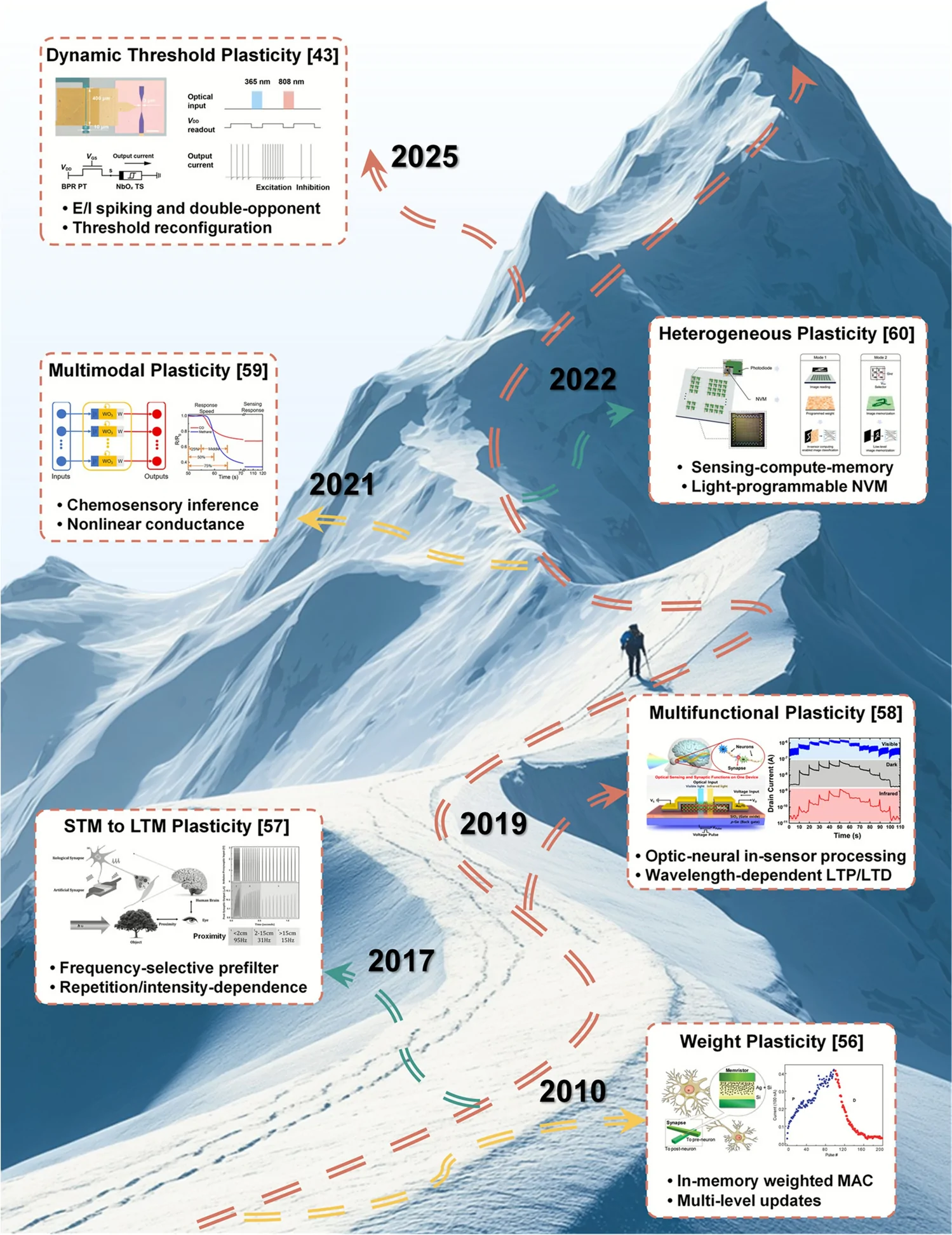
Roadmap of synaptic plasticity engineering. Weight Plasticity. Reproduced with permission [53]. Copyright 2010, American Chemical Society. Short-term memory (STM) and long-term memory (LTM) Plasticity. Reproduced with permission [54]. Copyright 2017, WILEY‐VCH Verlag GmbH & Co. KGaA, Weinheim. Multifunctional Plasticity. Reproduced with permission [55]. Copyright 2019, American Chemical Society. Multimodal Plasticity. Reproduced with permission [56]. Copyright 2021, The Authors. InfoMat published by UESTC and John Wiley & Sons Australia, Ltd. Heterogeneous Plasticity. Reproduced with permission [57]. Copyright 2022, The Authors. Advanced Optical Materials published by Wiley‐VCH GmbH. Dynamic Plasticity. Reproduced with permission [43]. Copyright 2025, The American Association for the Advancement of Science

The evolution of synaptic plasticity has shifted from early demonstrations emphasizing STM and LTM for precise weight updates in artificial synapses toward more sophisticated behaviors meeting emerging network training requirements, including multimodal integration, excitatory-inhibitory interactions, multifunctionality, and dynamic threshold modulation, thereby enabling enhanced model generalization and algorithm-level strategies for neuromorphic hardware, as illustrated in Fig. 2. Reflecting this conceptual trajectory, the review first examines the material and device engineering strategies that enable precise, stable, and high-resolution synaptic weight modulation. It then considers mechanisms of dynamic plasticity and threshold adaptation that are essential for temporal learning within RC and SNN architectures. Subsequent sections highlight higher-order synaptic behaviors such as wavelength selectivity, excitatory and inhibitory synergy, and metaplasticity, all of which play pivotal roles in enhancing algorithmic efficiency and adaptive learning. Further discussion addresses the impact of multifunctional and multimodal integration strategies in realizing compact, energy-efficient, and resource-conscious neuromorphic systems. The final section surveys recent advances in array-level integration and intelligent system demonstrations, underscoring the practical translation of these innovations into scalable and robust neuromorphic platforms.

## Synaptic Engineering for Analog Weight States

High-fidelity, multistate, and stable synaptic plasticity is the cornerstone of weight-driven neural networks [58]. Realizing analog conductance tuning with linear and symmetric long-term potentiation (LTP) and long-term depression (LTD) enables precise parameter updates, which are essential for achieving high-accuracy and robust learning performance [59]. In this context, expanding the resolution and linearity of synaptic weights and ensuring long-term weight stability and drift resistance have become parallel priorities in the development of advanced artificial synaptic devices. Together, these two facets underpin reliable weight mapping, efficient training convergence, and the scalable deployment of neuromorphic hardware in large-scale, parameter-driven networks.

### Multistate and Symmetric Weights

The realization of high-resolution, multistate, and linearly/symmetrically tunable weight modulation is fundamental to advancing artificial synaptic devices for neuromorphic computing [60]. In biological networks, the ability to finely and continuously modulate synaptic strength underpins the complex adaptive learning behavior of the brain, serving as an essential blueprint for weight-driven neural architectures such as convolutional and multilayer perceptrons [61]. In hardware implementations, this translates into the need for synaptic devices that can provide abundant, reproducible, and evenly spaced conductance states with minimal nonlinearity and variation, thereby ensuring precise weight mapping and stable training convergence [62]. Consequently, material and device engineering has been directed toward strategies that enhance the density, linearity, and symmetry of accessible conductance states, laying the foundation for high-accuracy and reliable performance in large-scale neuromorphic networks.

Among the early breakthroughs, Tian et al. reported an organic ferroelectric transistor synapse with a poly(vinylidene fluoride-trifluoroethylene) [P(VDF-TrFE)]/MoS_2_ structure, in which polarization-driven switching enabled more than 1000 quasi-continuous conductance states, providing a robust platform for precise and scalable weight modulation [63]. Subsequent efforts explored organic heterojunction synapses, for example, vertical p-n devices based on p-type poly(2,5-bis(2-octyldodecyl)-3,4-dicyanothiophene) (PDPP4T) and naphthalene tetracarboxylic diimide derivative (NTCDI-F15), which utilized enhanced exciton dissociation and suppressed recombination to achieve several hundred clearly separable potentiation-depression states with low LTP nonlinearity [64, 65]. In parallel, ferroelectric phototransistors based on α-In_2_Se_3_ exploited dynamic interfacial polarization switching, enabling highly symmetric, optically controlled bidirectional weight updates [66]. Along with innovations in quantum dot devices and charge transfer modulation, these advances have greatly broadened the palette for analog weight expression and conductance state engineering.

Nevertheless, persistent challenges, including residual nonlinearity, device variability, and incomplete symmetry, continue to hinder the widespread deployment of these systems in large-scale, high-accuracy neural networks. As a result, recent research has increasingly focused on optimizing a diverse range of material platforms, including organic, ferroelectric, two-dimensional, and heterostructure systems, each offering unique opportunities for achieving robust, high-density, and precisely controllable multistate weight expression [67].

**Fig. 3:**
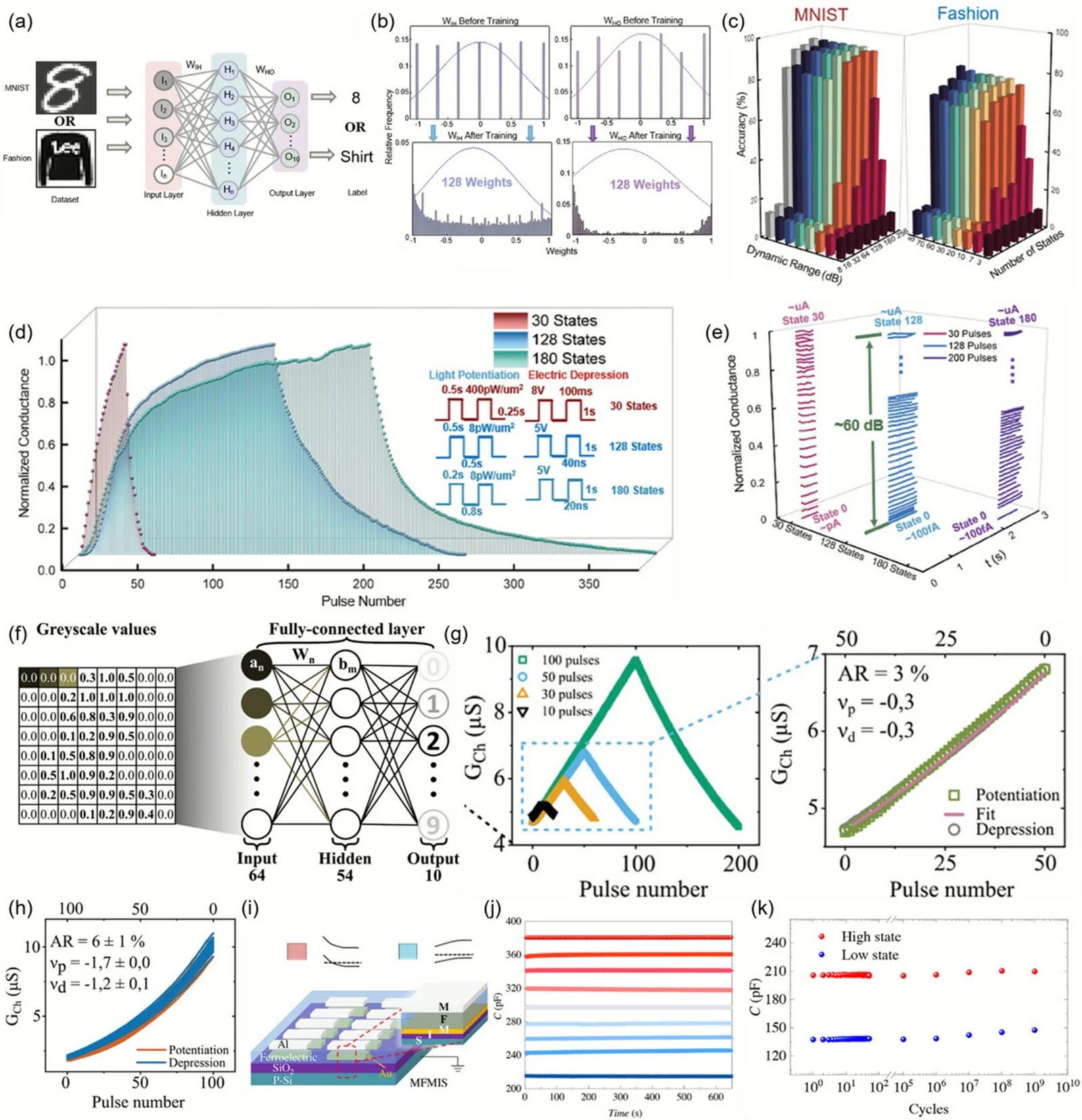
Multistate weight modulation and stability in neuromorphic synaptic devices. **a** Schematic of the multilayer neural network mapped from violet phosphorus (VP)-molybdenum disulfide (MoS_2_) synaptic devices. **b** Simulated weight distributions before and after training. **c** Classification accuracy versus dynamic range (DR) and state number. **d** Normalized long-term potentiation and depression (LTP/LTD) curves for VP-MoS_2_. **e** Representative waveforms for individual conductance states. **a-e** Reproduced with permission [35]. Copyright 2023 The Authors. Advanced Science published by Wiley‐VCH GmbH.** f** Neural network simulation using Bi_2_V_0.9_Cu_0.1_O_5.35_ (BICUVOX)/La_0.5_Sr_0.5_FeO_3-δ_ (LSF50) device weights for Modified National Institute of Standards and Technology (MNIST) digit recognition. **g** Linearity and symmetry of 50 potentiation/depression pulses. **h** Linearity and symmetry over all cycles. **f–h** Reproduced with permission [36]. Copyright 2024, Wiley–VCH GmbH. **i** Sketch of metal/ferroelectric/metal/insulator/semiconductor (MFMIS) memcapacitor array. **j** Robust retention of 3-bit capacitance states. **k** Cycle-to-cycle variation of high/low capacitance states. **j-k** Reproduced with permission [68]. Copyright 2023, The Authors. Exploration published by Henan University and John Wiley & Sons Australia, Ltd

A compelling example of this progression is found in the two-dimensional violet phosphorus (VP)–molybdenum disulfide (MoS_2_) heterostructure synaptic device, which sets a new benchmark for analog synaptic performance in neuromorphic computing [38]. The system-level significance of these advances is directly demonstrated through neural network simulations of image classification tasks: as shown in Fig. 3a, the synaptic weights of the VP-MoS_2_ device are mapped into a multilayer network model comprising 40,000 W_IH_ and 1,000 W_HO_ synapses, with both the training and inference stages faithfully emulating deep learning frameworks. The evolution of weight distributions before and after training (Fig. 3b) underscores the capacity of high-resolution analog weights to realize sharp and robust separation within large-scale networks. Crucially, systematic variation of dynamic range (DR) and state number (Fig. 3c) reveals that both parameters exert a decisive influence on classification accuracy: once DR drops below 20 dB, performance degrades precipitously, illustrating the fundamental necessity of high-fidelity, multistate synaptic mapping for complex pattern recognition. At the device level, the VP-MoS_2_ heterostructure leverages the wide bandgap and pronounced light–matter interactions of VP to achieve an ultrahigh dark-to-light ratio (> 10^6^), a dynamic range exceeding 60 dB, and 128 (7-bit) clearly separated conductance states (Fig. 3d, e). This exceptional state density is complemented by the device’s ability to support optically driven LTP and electrically driven LTD, both exhibiting highly reproducible and linear transitions across repeated programming cycles. The ultralow off-state current effectively minimizes weight mapping errors, a critical consideration for high-accuracy network applications.

To realize multistate, symmetric weights, evidence across material platforms points to a clear progression. Symmetric carrier injection barriers, shallow and narrowly distributed traps, and well-passivated interfaces produce monotonic, nearly uniform, and mirror-symmetric conductance updates. Ferroelectric channels then supply rapid and reversible polarization that avoids slow ionic drift, while organic and two-dimensional heterojunctions use interfacial charge transfer to suppress nonlinearity. Stability is reinforced by ion-blocking interlayers and robust dielectrics, which curb long-term drift and cycle-to-cycle variation. Wide bandgap channel stacks with ultralow off-state current further enlarge the dynamic range and raise the signal-to-noise ratio, reducing weight mapping error.

### Stable and Drift-Resistant Weights

The pursuit of highly stable, drift-resistant synaptic weight modulation stands as a foundational requirement for neuromorphic hardware targeting large-scale, weight-driven neural network applications [69]. Biological synapses possess the remarkable capacity for reliable and long-lasting information retention, a property underpinned by sophisticated ionic dynamics and homeostatic regulatory mechanisms [70]. In contrast, many artificial synaptic devices suffer from conductance drift, cycle-to-cycle variability, and progressive degradation under repeated operation or environmental fluctuations, which compromise weight precision, hinder training convergence, and limit system endurance [71, 72]. Addressing these challenges has thus become a central motif in synaptic device innovation, particularly as next-generation AI architectures demand robust in-memory computing platforms capable of both high-precision storage and operational endurance.

To overcome the volatility and reproducibility bottlenecks of conventional memristors, the community has developed a rich diversity of material and interface strategies aimed at suppressing drift and enhancing cycle stability [73]. For example, electrochemical synaptic transistors, especially those leveraging solid-state ionic conductors, have enabled finely tunable and highly stable analog weights through deterministic ion migration mechanisms. Among these, devices based on lithium- and proton-conducting electrolytes exhibit rapid switching and reasonable retention but are often hampered by environmental sensitivity and limited compatibility with standard CMOS processes [39, 68, 74].

Amidst these diverse approaches, a pivotal advance is exemplified by the solid-state oxide-ion synaptic transistor based on a Bi_2_V_0.9_Cu_0.1_O_5.35_ (BICUVOX) electrolyte and La_0.5_Sr_0.5_FeO_3-δ_ (LSF50) channel [39]. At the system level, the impact of such stable weight modulation is vividly illustrated by neural network simulations: when the experimentally derived BICUVOX/LSF50 weight curves are mapped into an 8 × 8 or larger-scale artificial neural network, the resulting Modified National Institute of Standards and Technology (MNIST) digit recognition accuracy reaches 96% (Fig. 3f). This not only approaches the ideal software benchmark but also demonstrates that minimizing device drift and ensuring long-term analog stability can markedly reduce inference errors and support sustained high-accuracy operation in large-scale, weight-driven neural networks. The remarkable device-level performance of BICUVOX/LSF50 underpins this system-level accuracy. Specifically, the device leverages the high ionic conductivity and thermal/environmental stability of the BICUVOX film to achieve deterministic, reversible, and low-voltage modulation of synaptic weights. Unlike earlier oxide-ion or protonic transistors that suffered from high switching voltages and unreliable ion migration, the BICUVOX/LSF50 system operates at sub-1 V levels, ensuring stable oxide-ion motion and minimal stochasticity in weight updates. This architecture yields more than 100 discrete, linearly spaced conductance states (7-bit precision), with a nonlinearity factor of 0.3–1.7 and an asymmetric ratio as low as 0.03, metrics that directly reflect its capacity for highly symmetrical and repeatable LTP/LTD modulation (Fig. 3g, h). Notably, these features are retained over 5000 programming cycles and persist even under elevated temperatures, attesting to the robustness of the device in edge computing and harsh environmental scenarios. Complementarily, Feng et al. introduced a ferroelectric fin diode (FFD) that achieved an exceptional endurance of over 10^10^ switching cycles together with stable analog memory states, highlighting the critical role of ferroelectric domain engineering in suppressing drift and ensuring long-term reliability for in-memory computing [75].

In addition, Tian et al. reported a ferroelectric memcapacitor network based on a P(VDF-TrFE)-integrated metal/ferroelectric/metal/insulator/semiconductor (MFMIS) structure, in which the stacked MFMIS configuration (Fig. 3i) enabled reconfigurable multilevel capacitance states governed by ferroelectric domain dynamics [76]. Benefiting from uniform polarization-induced fields, the device achieved stable intermediate states with retention times exceeding 10^4^ s and endurance beyond 10^9^ switching cycles, as confirmed by both cycle-to-cycle stability tests (Fig. 3j, k). In comparison, other contemporary platforms have also made notable strides in device stability and endurance [68, 73]. The novel solid-state sodium alginate (NaAlg)/ polyacrylic acid (PAA)/indium gallium zinc oxide (IGZO) device introduces a polyacrylic acid interface to buffer Na^+^ ion dynamics, achieving 64 stable conductance states over 12,000 cycles and supporting high-fidelity pattern recognition. Similarly, ultra-flexible Si nanomembrane arrays integrated with hybrid polyimide-Al_2_O_3_ dielectrics maintain high linearity, ultra-low conductance fluctuation (< 1.6%), and excellent endurance even after 10,000 bending cycles, achieving digit recognition rates up to 93.2%.

Across studies, consistent evidence indicates that drift-resistant and reproducible analog weights arise when ionic transport is deterministic in chemically and thermally robust solids, operating voltages are kept in the subvolt regime to confine dynamics to reversible ranges, polarization is tightly controlled, and interfaces are buffered or passivated. Under these conditions, devices deliver monotonic, nearly uniform, and mirror-symmetric conductance states with low nonlinearity and low asymmetry, and they retain these characteristics over thousands of programming cycles and at elevated temperatures. Endurance extends from 10^9^ to 10^10^ switching events, with intermediate-state retention on the order of 10^4^ s.

## Synaptic Engineering for Temporal Plasticity

The growing demand for neural networks that can process complex temporal signals in real-world environments has made dynamic synaptic plasticity central to the development of next-generation RC and SNNs [77]. Unlike conventional static architecture, temporal neural models require devices capable not only of rapid and reversible information encoding, but also of modulating memory retention and synaptic responsiveness on demand [78]. This shift presents two parallel challenges: first, how to implement tunable STM windows at the device level to enable real-time temporal correlation, and second, how to realize precise threshold and spike-timing-dependent plasticity for efficient event-driven learning and adaptive sequence recognition [79, 80].

### Tunable STM Windows

In the era of intelligent sensing and ubiquitous Internet of Things, neuromorphic hardware capable of encoding and manipulating dynamic temporal information has become foundational for advancing time-dependent machine learning architectures such as RC and SNNs [81]. While traditional feedforward artificial neural networks are optimized for static tasks, they lack the intrinsic STM necessary to capture, store, and process the temporal correlations inherent in real-world signals. This gap has driven the development of synaptic device platforms that directly emulate the transient, volatile, and highly tunable memory windows exhibited by biological synapses, attributes that underpin the temporal perception and adaptive response in natural neural circuits [82]. Building on this foundation, physical reservoir computing offers a promising route. It leverages the intrinsic nonlinear response and fading memory (FM) dynamics of devices. Through these properties, temporal inputs are projected into a high-dimensional state space, where simple linear readout layers can efficiently extract spatiotemporal correlations. The optimization of STM window depth and adaptability is therefore crucial, since it determines the trade-off between memory retention and nonlinear transformation, enhances the richness of reservoir states, and ultimately improves temporal encoding capacity and computational efficiency for edge AI applications [83].

**Fig. 4:**
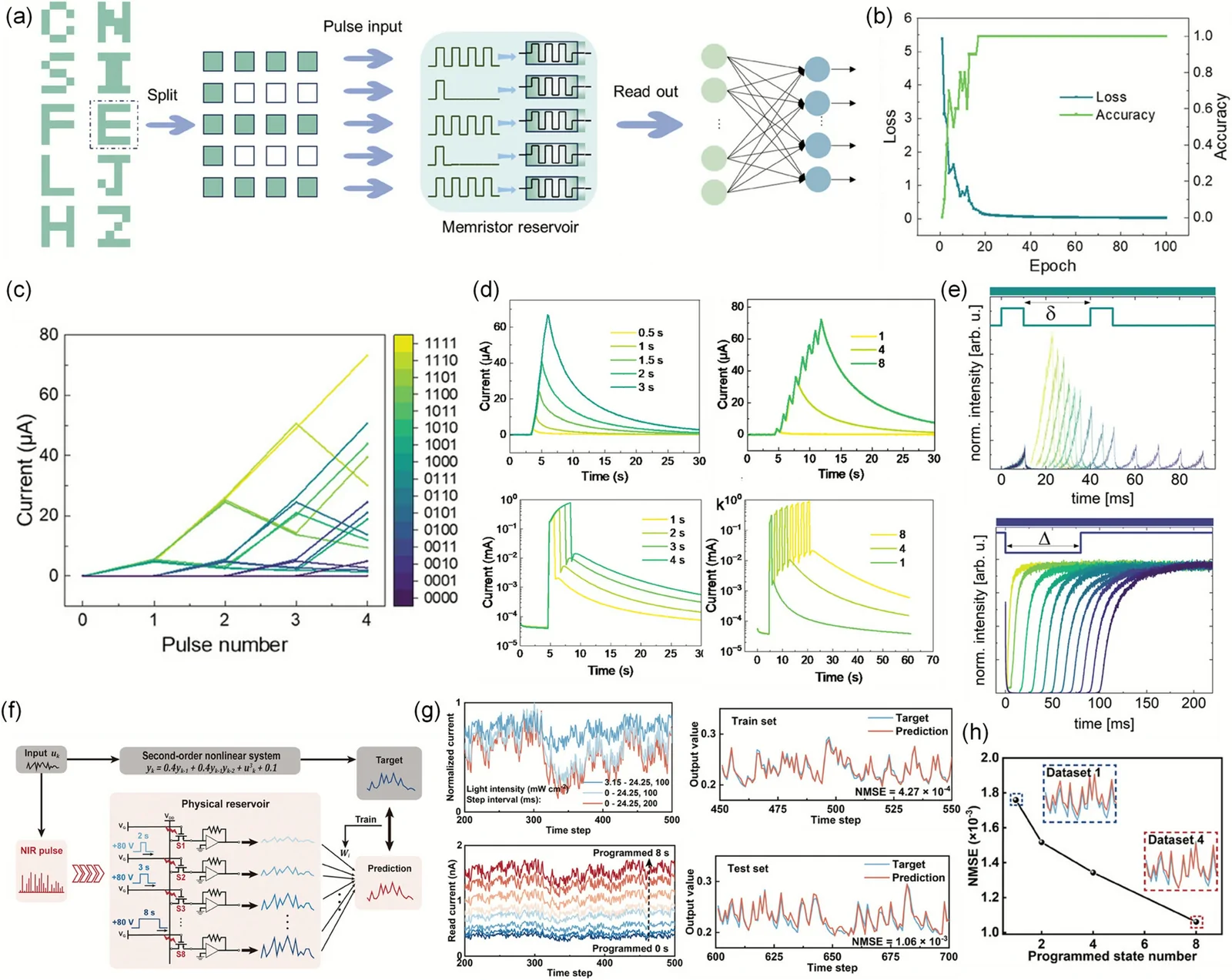
Temporal dynamics for tunable STM in neuromorphic hardware. **a** Schematic of pulse-encoded letter/image classification using in-sensor reservoir computing (RC). **b** Training accuracy and loss curves for the fully quantum dot optoelectronic memristor (FQDOM)-based system. **c** Excitatory postsynaptic current (EPSC) responses of optoelectronic memristors under different pulse sequences. **d** Modulation of STM window in FQDOMs by varying light/electrical pulse parameters. **a-d** Reproduced with permission [37]. Copyright 2025, Wiley‐VCH GmbH. **e** Paired-pulse facilitation (PPF) and transient photon-avalanche (PA) luminescence in nanocrystal-based all-optical synapses. Reproduced with permission [82]. Copyright 2023, The Authors. Advanced Materials published by Wiley‐VCH GmbH. **f** Schematic and current outputs of nonlinear dynamic task solving with core–shell upconversion nanoparticles (UCNP@SiO_2_)/ poly(3-hexylthiophene) (P3HT) reservoir. **g** Dynamic reservoir responses under different near-infrared (NIR) input and programmed states. **h** Prediction accuracy with increasing reservoir state diversity. **f–h** Reproduced with permission [38]. Copyright 2024, The Author(s). Advanced Materials published by Wiley‐VCH GmbH

A diverse array of material systems and device strategies has emerged to address this challenge, each contributing unique mechanisms for STM modulation. Among these, the fully quantum dot optoelectronic memristor (FQDOM), constructed from a ZnO QDs/CdSe QDs/ZnO QDs heterojunction, exemplifies an integrated approach that unifies broadband photodetection (ultraviolet–visible (UV) to red spectrum), nonlinear STM decay, color selectivity, noise-tolerant preprocessing, and reservoir computation within a single two-terminal volatile device, as shown in Fig. 4a [40]. As a physical reservoir, the FQDOM achieves near-ideal performance in dynamic tasks: in letter classification, nonlinear temporal mapping of pulse-encoded images results in 100% recognition accuracy within fewer than 30 training cycles (Fig. 4b, c). Furthermore, the ability to tune memory decay and synaptic response by varying pulse number, intensity, and spectral content extends the diversity of accessible reservoir states, a critical enabler for high-dimensional temporal information processing (Fig. 4d).

Parallel innovations in all-optical synaptic platforms, such as photon-avalanche (PA) nanocrystals, leverage excited-state absorption and energy looping within upconversion nanoparticles to yield ultrasteep nonlinear luminescence dynamics and robust STM behavior [84]. The PA system demonstrates an exceptionally high PPF index that depends sensitively on inter-pulse delay (Fig. 4e), faithfully replicating the FM enhancement observed in biological synapses. Such all-optical synaptic models not only enable dynamic feature extraction and temporal summation in pure photonic domains but also facilitate hardware-embedded preprocessing for sequence-based neuromorphic computing, eliminating the need for external network training.

Extending the STM paradigm to the near-infrared, Leng et al. introduced a hybrid transistor architecture based on core–shell upconversion nanoparticles (UCNPs@SiO_2_) embedded in a poly(3-hexylthiophene) (P3HT) channel [41]. This device exploits photon-electron coupling and electrical programming to achieve multilevel nonvolatile conductance states (≥ 8), adjustable relaxation times, and rich nonlinear and asymmetric memory dynamics under narrow-band near-infrared (NIR) stimulation. The resulting reservoir enables in situ encoding and computation for both static and dynamic pattern recognition, achieving, for instance, 91.13% accuracy in static digit classification and a normalized mean squared error as low as 1.06 × 10^–3^ in predicting complex nonlinear dynamic sequences (Fig. 4f–h). Crucially, the expansion of reservoir states via variable programming and optical input parameters translates directly to enhanced prediction accuracy and adaptability in time-dependent computational tasks, highlighting the value of tunable STM windows and multimodal input fusion for temporal neuromorphic platforms.

Across neuromorphic platforms, the aim is to realize tunable temporal memory and coding that support reservoir computing and spiking models operating on real-world time-varying signals. When devices combine intrinsic nonlinearity with fading memory dynamics and allow on-demand modulation of retention and responsiveness, temporal inputs are embedded into high-dimensional state trajectories that preserve temporal correlations while remaining linearly decodable. Heterojunction stacks, core–shell photonic architectures, and engineered energy-transfer pathways stabilize excited-state kinetics and broaden spectral responsivity, yielding controllable volatility with reduced environmental sensitivity. Controlling pulse number, amplitude, width, inter-pulse interval, and spectral content tunes short-term memory depth and relaxation times, enhances temporal summation, and expands the accessible reservoir state space.

### Spike-Timing Plasticity

The pursuit of hardware-efficient SNNs has elevated dynamic threshold modulation and STDP to the forefront of neuromorphic device innovation [85]. Biological systems leverage precisely timed spiking and adaptive membrane thresholds to achieve energy-efficient encoding and rapid learning of temporal patterns [86]. In this context, SNNs introduce the principle of event-driven computation, where neurons accumulate inputs until a dynamic threshold is reached and emit discrete spikes that encode information in their timing and frequency [87]. Through STDP, the precise correlation between pre- and postsynaptic spikes enables local and unsupervised weight updates, which not only replicate biological learning rules but also enhance the temporal precision, energy efficiency, and adaptability of hardware SNNs [88].

Emulating these dynamics in hardware, however, remains a formidable challenge, particularly in the compact, low-power integration of leaky-integrate-and-fire (LIF) neuron behavior, spike frequency adaptation, and direction-selective learning. While early CMOS- and MOSFET-based artificial neurons provided proof-of-concept LIF operation, they were hindered by high power consumption and bulky form factors [89]. To address these limitations, recent research has focused on developing novel device architectures and material systems that more closely emulate the rich temporal dynamics of biological neurons and synapses. Of particular interest are steep-slope, energy-efficient devices and co-integrated systems capable of precise temporal information processing and unsupervised learning. Among these advances, device-level innovations that directly implement both LIF spiking and synaptic plasticity within scalable, low-power hardware have demonstrated particularly promise for next-generation SNNs [90].

**Fig. 5:**
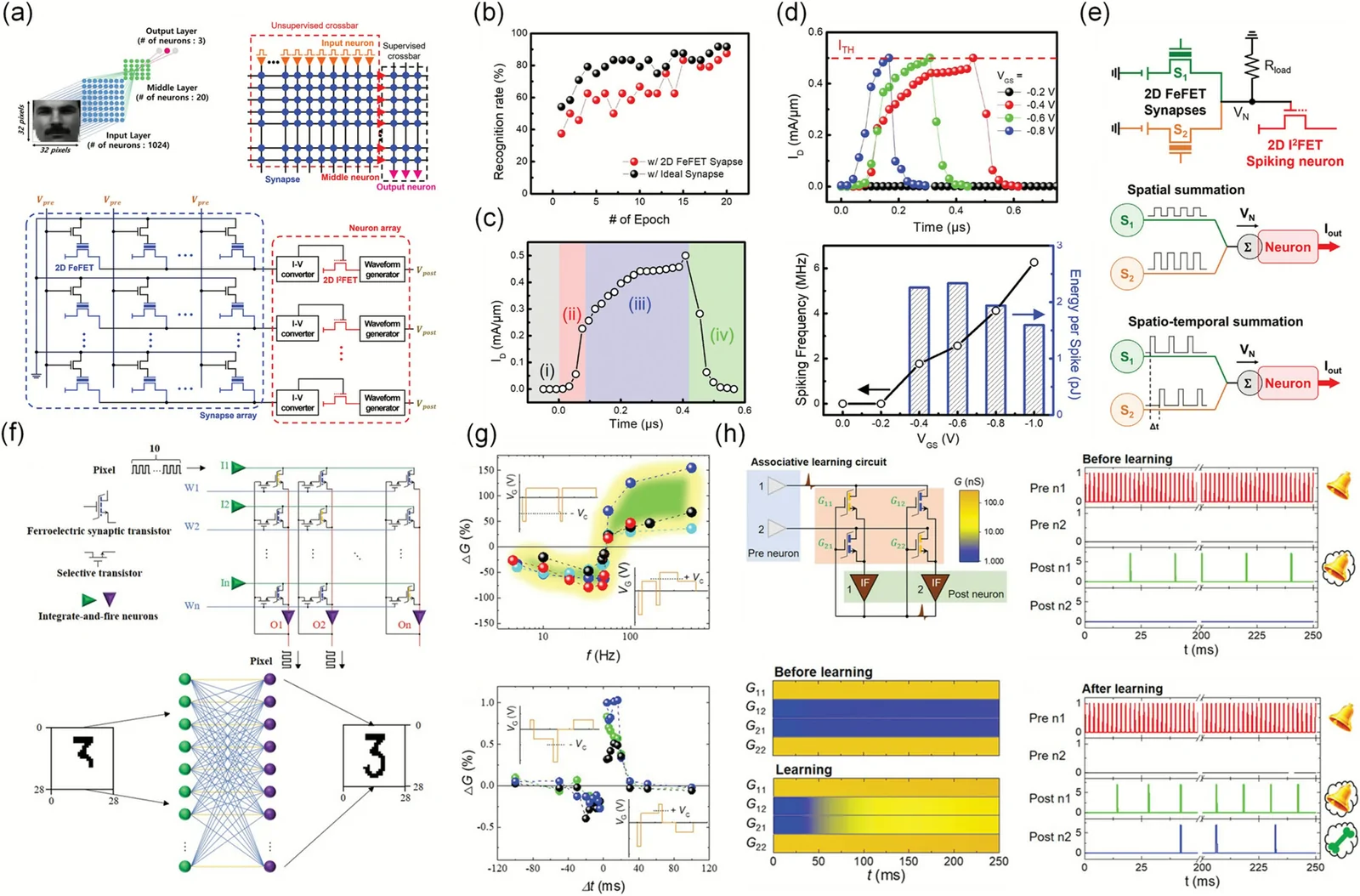
Spiking neural networks (SNN) implementation and device mechanisms for dynamic threshold modulation and spike-timing-dependent plasticity (STDP). **a** Simulation of face classification with a 2D SNN: spiking neural network scheme for 32 × 32 grayscale image recognition, including crossbar layouts (unsupervised and supervised layers) and device-based pseudo-crossbar array design with α-In_2_Se_3_ ferroelectric transistor (FeFET) synapses and tungsten diselenide (WSe_2_) impact-ionization transistor (I^2^FET) neurons. **b** Recognition rate of the SNN as a function of the number of training epochs. **c** Leaky-integrate-and-fire (LIF) operation of I^2^FET neuron. **d** Spiking characteristics and frequency modulation of the I^2^FE neuron. **a-e** Reproduced with permission [39]. Copyright 2024, Wiley‐VCH GmbH. **e** Spatiotemporal summation and circuit integration in the SNN. **f** FeFET network for associative memory and one-shot pattern completion. **g** Demonstration of Hebbian plasticity: SRDP and STDP in FeFET synapses. **h** Associative learning realized by the FeFET synaptic network. **f–h** Reproduced with permission [40]. Copyright 2021, Wiley‐VCH GmbH

Choi et al. proposed a significant breakthrough by demonstrating fully two-dimensional material-based SNNs that integrate WSe₂ impact-ionization ferroelectric FET (I^2^FET) neurons with α-In_2_Se_3_/hexagonal boron nitride (h-BN)/CuInP_2_S_6_ (CIPS) ferroelectric FET (FeFET) synapses in Fig. 5a [42]. Notably, this platform achieved an impressive 87.5% accuracy in unsupervised face classification after only 20 training epochs, substantially outperforming typical low-parameter SNNs (Fig. 5b). At the device level, the 2D (I^2^FET neuron exploits a locally ungated, high-field region within the tungsten diselenide (WSe_2_) channel to enable abrupt, sub-microsecond avalanche spiking at ultralow energy consumption (~ 2 pJ/spike), a 20- to 5000-fold reduction compared to conventional silicon neuron circuits (Fig. 5c). This architecture ensures linear spike-frequency modulation with input bias, conferring robust event-driven adaptability and precise threshold dynamics, critical for real-time temporal coding and event detection, as shown in Fig. 5d. System-level integration with FeFET synapses allows each artificial neuron to perform both spatial and spatiotemporal integration of distributed, asynchronously timed inputs, faithfully recapitulating dendritic computation and gating in biological systems (Fig. 5e). Experiments demonstrate that individual subthreshold inputs may be insufficient to trigger a spike, while coincident or temporally proximate inputs summate efficiently to elicit firing, underscoring the network’s ability to capture complex temporal features with high selectivity.

Equally remarkable, the MoS_2_-based ferroelectric synaptic transistor associative spiking neural network (aSNN) demonstrates state-of-the-art associative memory and one-shot completion capabilities [43]. In digit completion tasks, the aSNN can accurately reconstruct entire patterns from partial cues, achieving a classification accuracy of up to 91.13% in static digit tests, surpassing traditional iterative associative networks (Fig. 5f). At the device level, the ferroelectric synapse harnesses gate-controlled domain wall dynamics to enable analog, symmetric, and linear weight modulation with sub-femtojoule energy per event, supporting a full suite of short-term repetitive depression/potentiation (SRDP) and STDP learning rules (Fig. 5g), where the relative timing of pre- and postsynaptic spikes governs LTP or LTD. This permits on-device, unsupervised Hebbian learning and LTM retention (Fig. 5h).

In neuromorphic hardware, an important direction is hardware-efficient, event-driven computation achieved by coupling adaptive thresholding with STDP. Deterministic leaky-integrate-and-fire neurons with linear rate control, paired with synapses that deliver analog, symmetric, and linear weight updates from precise spike timing, enable accurate temporal coding and local unsupervised learning. High field regions, ferroelectric or dielectric domain control, and interface passivation stabilize thresholds and plasticity kinetics while limiting leakage and variability. Tuning threshold set points and adaptation, shaping spike timing and intervals, and operating at latency and per spike energy improve efficiency and selectivity. Co-integrating steep-slope neuron devices with low energy, timing-sensitive synapses, aligning STDP windows to task time constants, and maintaining low voltage operation yield robust coincidence detection, associative recall, and competitive accuracy with few training epochs for edge-scale temporal intelligence.

## Synaptic Engineering for Context-Aware Sensory Gating

The realization of robust, intelligent sensory perception in neuromorphic systems increasingly depends on the ability to selectively extract salient features, suppress interference, and flexibly adapt to complex, dynamic environments [91, 92]. Achieving these functions at the hardware level requires artificial synaptic devices that go beyond simple signal transduction, integrating a rich diversity of programmable plasticity mechanisms for context-aware information processing [93]. Rather than relying on fixed or single-modal responses, state-of-the-art sensory synaptic architectures must dynamically encode spectral, spatial, and contextual cues to enable high-fidelity feature encoding, efficient attention, and adaptive learning in the presence of noise and uncertainty [94, 95].

### Wavelength-Selective Response

The capacity for wavelength-selective perception and encoding is foundational for the advanced feature extraction and robust information processing demanded by next-generation neuromorphic vision systems [96, 97]. Biological retinas achieve these functions through diversified photoreceptor responses, most notably, the selective encoding of colors by rod and cone cells, to filter redundant background signals and accentuate task-relevant spectral features [98, 99]. This architecture enables color discrimination and noise-tolerant recognition even in challenging and complex environments [28, 100]. Motivated by this natural paradigm, artificial visual synapses are being engineered to exhibit customized photoresponse windows and spectrum-specific memory retention, with innovations encompassing ion-doped perovskite nanocrystals, molecular cocrystal networks, π-π hybrid nanocomposites, and plasmonic 2D heterostructures, thereby unlocking new directions for hardware-level, context-aware spectral processing [44, 101–103].

**Fig. 6:**
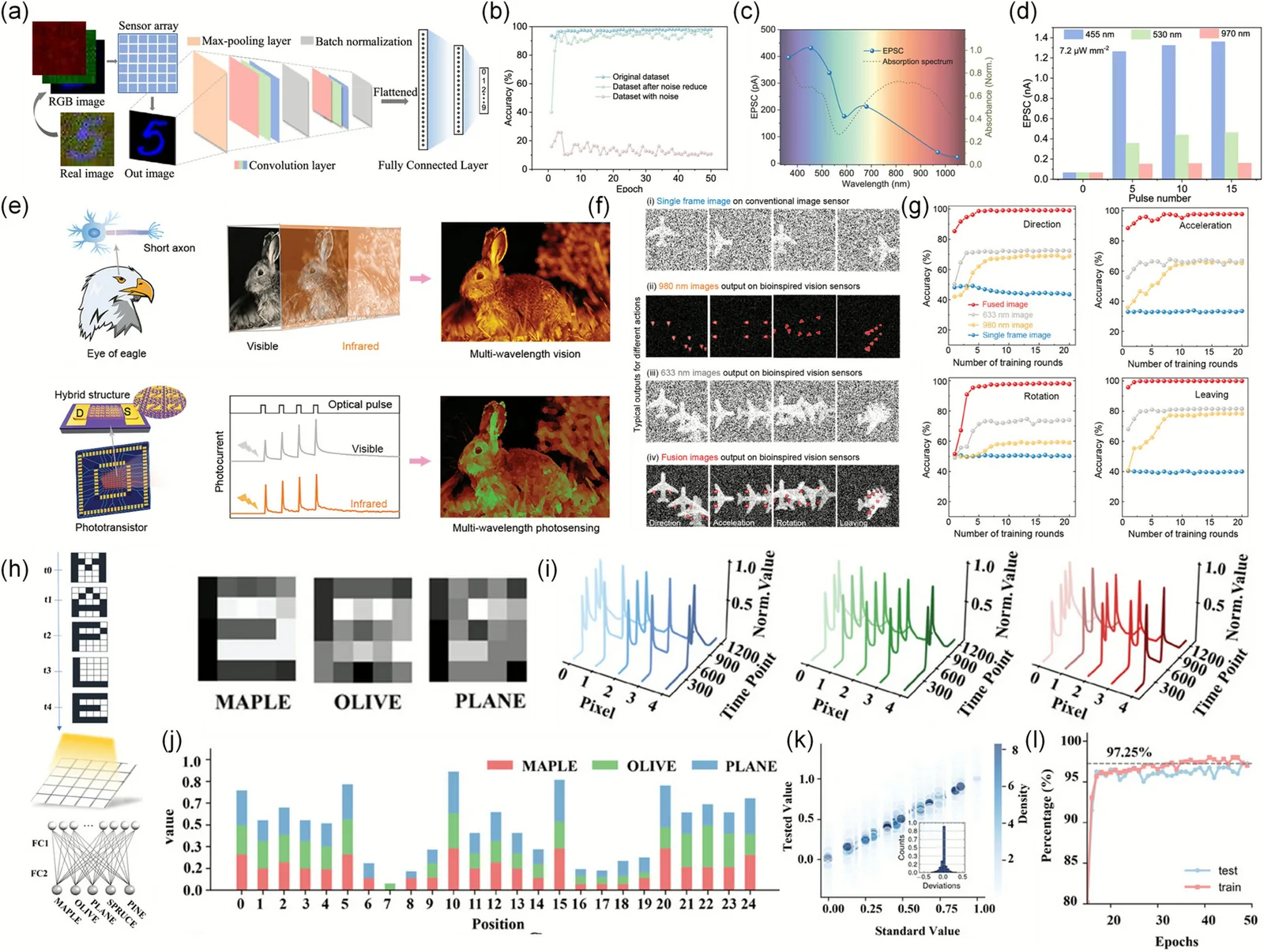
Wavelength-selective synaptic plasticity for spectral perception and feature encoding. **a** Schematic of molecular cocrystal nanowire (MCN) synaptic sensor array and convolutional neural networks (CNN) for image denoising and color feature extraction. **b** Denoising with MCN array in noisy MNIST images. **c** MCN synapse exhibits broadband photoresponse under ultraviolet–visible (UV), visible, and NIR light. **d** Selective EPSC response of MCN synapse to red, green, and blue light pulses. **a-d** Reproduced with permission [96]. Copyright 2025, American Chemical Society. **e** Schematics of eagle-inspired dual-band visual fusion and optoelectronic vision array for multiwavelength sensing. **f** Image fusion sensor in motion recognition and spatiotemporal information capture. **g** Dual-band fusion sensors across various dynamic tasks. **e–g** Reproduced with permission [41]. Copyright 2024, Wiley‐VCH GmbH. **h** Sequential letter videos drive fullerene (C_60_)@graphene oxide (GO) array, with final frame current used for classification. **i** Output currents of C_60_@GO array during sequential letter input encode spatiotemporal dynamics. **j** Normalized feature vectors from the final frame enable sequence-specific classification. **k** Device demonstrates robust, monotonic temporal mapping over repeated trials. **l** Lightweight CNN based on temporal readout of the device array. **h–l** Reproduced with permission [97]. Copyright 2025, The Author(s). Advanced Science published by Wiley‐VCH GmbH

Recent research highlights a flourishing diversity of underlying mechanisms for wavelength-specific synaptic plasticity, as exemplified by several representative works. In the study by Dong et al., the perylene-7,7,8,8-tetracyanoquinodimethane (TCNQ) molecular cocrystal nanowire (MCN) synapse leverages highly ordered donor–acceptor charge transfer to achieve both broadband UV–Vis-NIR responsivity and efficient exciton dissociation [101]. As illustrated in Fig. 6a, their MCN synaptic sensor array is tightly integrated with a CNN, forming a complete workflow for blue-targeted image preprocessing and high-precision feature recognition. The quantitative advantage of this scheme is reflected in Fig. 6b, where denoising red–green Gaussian-corrupted MNIST images using the MCN array increases recognition accuracy from only 12% (raw input) to 90%, close to the performance on clean data. The material’s robust broadband photoresponse across 365–1050 nm is further demonstrated in Fig. 6c, while Fig. 6d underscores the device’s selective and strong excitatory postsynaptic current (EPSC) response under pulsed blue (455 nm) light, essential for targeted blue feature isolation from complex backgrounds.

For dynamic and motion-rich environments, Huang et al. advance the field with a plasmon-enhanced 2D MoS_2_ neuron array, drawing direct inspiration from the architecture of eagle eyes [44]. Figure 6e depicts the schematic of the eagle-inspired visual system, emphasizing the role of short axons and dual-wavelength integration, key principles guiding the design of their hybrid plasmonic/2D semiconductor optoelectronic neuron arrays. Figure 6f contrasts the imaging outcomes of conventional, visible-only, NIR-only, and dual-band fusion sensors, revealing that dual-band fusion yields the most information-rich spatiotemporal frames for motion analysis. This systematic advantage is clearly quantified in Fig. 6g, where recognition of four different motion types with the neural network reaches near-perfect accuracy (≈ 99.8%) using fused visible-NIR inputs, dramatically surpassing all single-band and conventional approach and highlighting the practical impact of biomimetic, multispectral sensing.

Addressing the challenge of sequence-dependent visual information encoding, Liu et al. developed a π-π coupled fullerene (C_60_)/graphene oxide (GO) heterosynaptic array, implemented in a 5 × 5 configuration for real-time video-based recognition [102]. Figure 6h presents the experimental scheme, where sequential letter inputs generate distinctive spatiotemporal current maps within the device array. The array’s capacity to accumulate and store temporal information is evidenced in Fig. 6i, with the output for the final frame (“E”) reflecting the memory of the full stimulus sequence. Figure 6j further details the classification vectors, where each word produces unique feature maps as a function of prior input. Figure 6k demonstrates the device’s robust, monotonic current variation with pulse number, supporting precise and noise-resistant temporal mapping. Ultimately, as summarized in Fig. 6l, a lightweight CNN readout trained on these temporal signatures delivers a dynamic video recognition accuracy of 97.3%, confirming both excellent generalization and high noise robustness.

Across neuromorphic vision platforms, the objective is wavelength-selective sensing and encoding that support robust feature extraction. Spectrally tailored absorption, efficient exciton dynamics, and photogating, combined with plasmonic coupling or π stacking, produce spectrum-specific plasticity and retention that suppress background while emphasizing salient cues. Donor acceptor cocrystals, plasmonic and two-dimensional heterostructures, and π-stacked composites define photoresponse windows, enhance local fields, stabilize spectrum-specific memory, expand dynamic range, and improve readout signal to noise; tuning pulse number, amplitude, interval, and spectral content adjusts short-term memory depth and spatiotemporal mapping. Prioritizing stacks that couple spectrum-specific gain with controllable retention, and matching device time constants to task timescales, yields noise-tolerant color and motion perception that lightweight readout networks can decode.

### Excitatory and Inhibitory Synergy

The evolution of neuromorphic visual systems is fundamentally driven by the pursuit of artificial retinas capable of emulating the human eye’s sophisticated balance of feature enhancement and noise suppression [104]. In biological vision, the retina achieves high-fidelity perception through the orchestrated synergy of excitatory and inhibitory pathways in bipolar and ganglion cells, allowing for the selective amplification of salient cues, real-time dynamic background suppression, and spatially adaptive attention [105, 106]. This architecture not only underpins robust image preprocessing, such as edge detection, contrast enhancement, and motion sensitivity, but also facilitates energy-efficient, context-aware decision-making at the sensor level [107]. However, the majority of existing implementations still depend on hybrid electrical/optical control or unidirectional (predominantly excitatory) modulation, which constrains their capacity for real-time, low-power, and context-aware attention mechanisms, especially when precise bidirectional (excitatory and inhibitory) spatial encoding is required for tasks such as edge detection, motion discrimination, and dynamic background suppression [108, 109].

While artificial synaptic devices, especially optoelectronic memristors, have enabled progress toward this biomimetic goal, most conventional systems still rely on hybrid electrical/optical modulation or exhibit only unidirectional (excitatory) response profiles, thereby limiting their capacity to implement real-time, hardware-level attention and bidirectional spatial encoding [36]. Addressing these challenges, the field is experiencing a paradigm shift toward fully optical, symmetric bidirectional modulation of synaptic weights [110]. Such advances are rapidly setting new benchmarks for neuromorphic hardware by enabling spatially resolved, energy-efficient in-sensor computation, and providing direct analogs to biological processes of context-sensitive information filtering and feature selection [111].

**Fig. 7:**
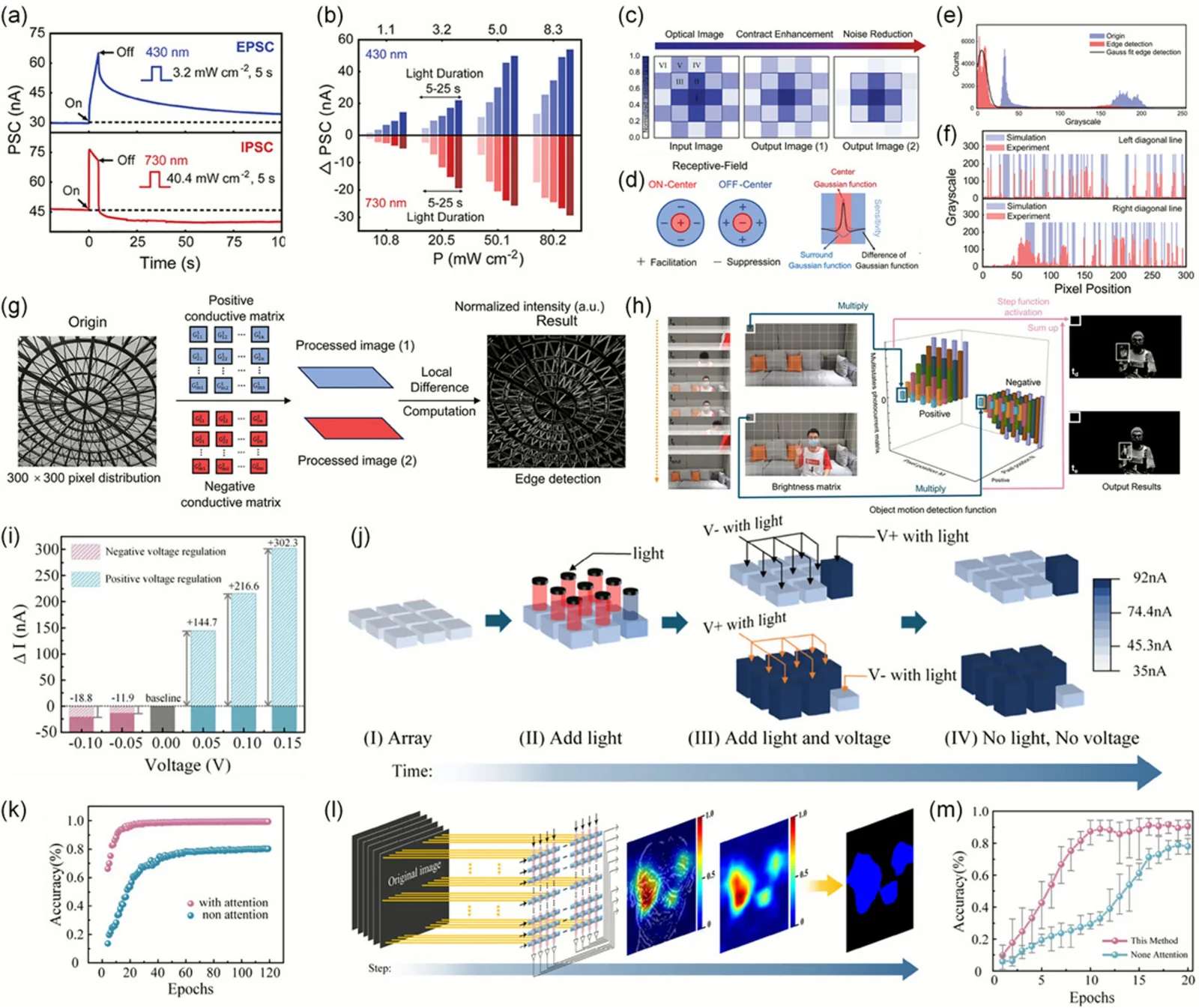
Excitatory and inhibitory synergy and spatial attention in neuromorphic vision systems. **a** Light-driven, reversible EPSC/ inhibitory postsynaptic current (IPSC) in zinc oxide (ZnO)/zinc methyl 3-devinyl-3-hydroxymethyl-pyropheophorbide-*a* (Chl-A)/methyl 13^1^-deoxo-13^1^-dicyanomethylene-pyropheophorbide-*a* (Chl-D) heterojunction memristor. **b** Robust bidirectional LTP/LTD switching under varying illumination. **c** 5 × 5 memristor array enables real-time image contrast enhancement and denoising. **d** Receptive field models for hardware edge detection. **e** Pixel distribution of 300 × 300 input image for edge extraction. **f** Hardware versus Canny operator edge detection, validating biological plausibility. **g** Large-scale edge extraction via superposed conductance matrices. **a-g** Reproduced with permission. Copyright 2024, Wiley‐VCH GmbH. **h** Motion detection by sequential conductance subtraction in WSe_2_/poly(vinylidene fluoride-trifluoroethylene) (P(VDF-TrFE)) sensors. Reproduced with permission [105]. Copyright 2024, American Chemical Society. **i** Region-specific optical gain control in indium tin oxide (ITO)/Nb:SrTiO_3_ synapses. **j** Programmable spatial attention in a 3 × 3 synapse array with memory retention. **k** Accuracy curve shows improved accuracy with hardware attention. **l** Class activation mapping highlights key facial features. **m** Device-level attention boosts ORL facial recognition. **i-m** Reproduced with permission [106]. Copyright 2025, American Chemical Society

A landmark advance in this direction is exemplified by the zinc oxide (ZnO)/zinc methyl 3-devinyl-3-hydroxymethyl-pyropheophorbide-*a* (Chl-A)/methyl 13^1^-deoxo-13^1^-dicyanomethylene-pyropheophorbide-*a* (Chl-D) heterojunction optoelectronic memristor, as proposed by Jiang et al. [45]. By leveraging spectrally selective photoionization and deionization of oxygen vacancies at the interface, this device enables precise, fully light-driven potentiation and inhibition, mirroring the antagonistic behavior of retinal bipolar cells. Figure 7a demonstrates distinct EPSC and inhibitory postsynaptic current (IPSC) responses under 430 and 730 nm light, respectively. The reversibility and stability of this bidirectional modulation, as evidenced in Fig. 7b, support robust LTP/LTD switching and underline the device’s potential for long-term synaptic encoding. Figure 7c extends these findings to array-level image preprocessing: using a 5 × 5 memristor grid, image regions are selectively amplified or suppressed according to luminance, implementing spatial contrast enhancement and dynamic noise reduction directly at the hardware level. This center-surround antagonism, functionally analogous to biological receptive fields, is further supported by the hardware extraction of object edges (Fig. 7d), where the device-based edge maps closely parallel those generated by computational Canny operators. The Gaussian-like pixel distribution of processed images (Fig. 7e) and the high degree of experimental-computational agreement (Fig. 7f) provide quantitative validation of the biological plausibility and precision of this approach. For large-scale or high-resolution edge extraction, Fig. 7g details the construction of a 300 × 300 positive–negative conductance matrix, establishing the scalability and robustness of this optical strategy.

Crucially, this excitatory and inhibitory synergy is not limited to static feature enhancement but also underpins advanced dynamic feature selection. For example, in reconfigurable WSe_2_/P(VDF-TrFE) neuromorphic vision sensors proposed by Dang et al., symmetric, nonvolatile bidirectional photocurrent states enable the encoding of temporal frame differences [112]. As shown in Fig. 7h, the subtraction of sequentially programmed positive and negative conductance matrices cancels static backgrounds and accentuates motion, directly mimicking ganglion cell dynamics and yielding high-accuracy gesture and trajectory recognition when integrated into neural networks.

Beyond basic edge and motion processing, the ability to dynamically weight spatial regions at the device level paves the way for high-level, hardware-based attention mechanisms. Wang et al. proposed indium tin oxide (ITO)/Nb:SrTiO_3_ heterojunction synapses, where voltage-assisted optical modulation enables real-time, region-specific tuning of synaptic gain [113]. As depicted in Fig. 7i, a positive bias enhances the response of target regions, while a reverse bias suppresses irrelevant signals. Figure 7j illustrates this principle using a 3 × 3 device array, with spatially programmed voltages precisely focusing or defocusing attention. The effectiveness of this biomimetic attention mechanism is further confirmed in Fig. 7k, which uses a color confusion matrix to visualize classification results; pixel darkness directly maps to the accuracy of predicted categories, demonstrating that the artificial synapse substantially boosts recognition performance by extracting key information and suppressing background interference.

Building on these foundational mechanisms, the integration of artificial synaptic arrays with neural network models enables intelligent and robust recognition in complex environments. Figure 7l presents a system for high-precision facial recognition, where synaptic resistive states are mapped to critical facial features, and class activation mapping (CAM) visually identifies regions of highest relevance. The network’s learning curve, documented in Fig. 7m, shows that the incorporation of device-level attention raises recognition accuracy on the ORL dataset from 77 to 90% and reduces data load by 35%–65%, even under noise and spatial distortions. These results underscore not only the device’s precise attention-guided recognition capability but also its reliable cycle-to-cycle memory retention and adaptability to real-world challenges.

Complementary progress is observed in all-optical cadmium sulfide (CdS)/graphene/Ge and tin selenide (SnSe) thin-film synapses, which offer continuously tunable, symmetric persistent photoconductivity (PPC) and negative photoconductivity (NPC) modulation and high-fidelity weight updating [114, 115]. These devices support advanced convolutional operations, real-time motion tracking, and integrated in-sensor computations, including Gaussian blurring, sharpening, and dynamic suppression, entirely within the photonic domain and without the need for complex digital conversion or additional circuitry. Collectively, these advances delineate a clear pathway toward fully integrated, scalable, and context-aware neuromorphic visual processors.

Progress in neuromorphic vision hinges on retinal-inspired, context-aware encoding that balances feature enhancement and noise suppression. Spectrally selective photoionization–deionization and photogating that realize fully optical, symmetric potentiation and inhibition, together with center-surround antagonism at the device or array level, amplify task-relevant spectral and spatial cues while suppressing background. Heterojunction stacks with engineered interfacial vacancies and bias-assisted optical gating define precise photoresponse windows, stabilize reversible LTP/LTD, and sustain spectrum-specific retention. Prioritizing fully light-driven symmetric weight control and receptive field antagonism, matching device kinetics to frame rates, and using region-specific gain raises recognition accuracy, reduces data load, and enables integrated, energy-efficient, context-aware vision.

### Adaptive Threshold Modulation

The continuous evolution of neuromorphic computing is fundamentally driven by the aspiration to emulate the brain’s extraordinary capacity for adaptive learning, environmental robustness, and context-aware response, capabilities intrinsically rooted in the dynamic modulation of neuronal activation thresholds and higher-order metaplasticity [116]. In biological circuits, such threshold adaptation and metaplasticity underpin not only stable memory formation but also flexible, experience-driven learning in noisy or weak-signal environments [117]. Hardware realization of these functions has long been constrained by the static, first-order behavior of conventional synaptic devices, which often fail to reconcile low power, tunable thresholds, and biologically realistic learning dynamics [118]. Framed as a plasticity engineering strategy rather than a biological analogy, adaptive threshold control supports both neural precision and temporal learning [119]. For precision, programmable thresholds confine activations to informative ranges, suppress spurious activations in low contrast scenes, allocate dynamic range to salient inputs, and help preserve linear and symmetric weight updates; together, these effects improve separability, raise readout signal-to-noise ratio, and stabilize convergence in parameter-driven networks. These roles are consistent with metaplastic control in biology and with recent device strategies for threshold tuning in ferroelectric and related materials [88, 120]. For temporal learning, threshold adaptation shapes short-term memory windows, regulates spike rate and refractoriness, and sets the timing sensitivity required for sequence encoding and spike timing-dependent plasticity, thereby enhancing correlation capture and event selectivity in reservoir and spiking models [121–123]. Recent advances have yielded a diverse suite of material strategies and device architectures that directly implement tunable threshold and metaplastic behaviors, empowering artificial neural networks to adjust their sensitivity and learning rate in real time, a critical leap for handling low-contrast, noisy, or dynamically varying inputs in both convolutional and spiking models [124, 125].

A paradigm shift in adaptive hardware is exemplified by Wang et al.’s dual-adaptive heterojunction synaptic transistor, where photoadaptive threshold sliding and voltage-history-dependent metaplasticity are seamlessly integrated in a single organic p-n heterojunction [119]. Unlike earlier single-mode synaptic devices that implemented only photoadaptation or only history-dependent plasticity, this co-integration co-regulates input dynamic range and learning rate baselines within one element, reducing external contrast handling and calibration overhead and demonstrating a wider operating envelope across varying contrast and noise conditions. This system exhibits bidirectional photoconductivity: light-intensity-modulated photogating supports in-sensor preprocessing such as automatic contrast enhancement and edge sharpening, while dynamically lowering the synaptic depression threshold for adaptive memory erasure under strong illumination. Robust metaplasticity is achieved via unipolar spike-voltage-dependent plasticity (U-SVDP), allowing the LTP/LTD transition point to slide with stimulus history and incorporating an enhanced depression effect (EDE) that encodes experience-dependent inhibition. This second-order plasticity accelerates network convergence fivefold and increases CNN recognition accuracy from 91.2 to 93.8%, even under ultra-low contrast (down to 0.4%) and high noise, underscoring the critical role of threshold modulation in resilient neuromorphic learning.

In parallel, Zhang et al. address the rigidity of static transfer functions by introducing self-sensitizable artificial neurons based on perovskite nickelate (NdNiO_3_), where adaptive hydrogen gradients enable dynamic modulation of spiking thresholds [126]. Plasticity-engineered threshold and gain control reshape the input acceptance range in real time, enabling seamless adaptation between low-intensity and high-intensity scenes and yielding an approximately 250% increase in processed structural information for complex scene detection and classification. These neurons deliver persistent, programmable excitability and selective noise filtering, ensuring robust edge detection and object recognition under substantial environmental drift.

Extending these concepts to multimodal sensing, Lv et al. present a humidity-responsive neuron using cyclo(-Tyr-Tyr) peptide nanowires, in which proton-coupled Ag^+^ migration supports humidity-dependent, ultra-low-voltage threshold switching (≤ 0.1 V) [127]. Mechanistically, ambient humidity increases proton activity in the peptide network, which lowers the activation barrier for Ag^+^ hopping so that each stimulus pulse drives reversible ionic accumulation and release, yielding conductance transients that map pulse amplitude or frequency into spike amplitude or rate. This enables analog environmental signals to be converted into strength-coded spike trains, closely mimicking biological hygroreception and spike encoding. When implemented in spiking networks, these humidity neurons achieve 92.68% diagnostic accuracy for respiratory disease classification, highlighting the expansion of adaptive thresholding to wearable health diagnostics.

At the ionic circuit level, Mei et al. advance transmembrane-potential-gated MXene ionic transistors that emulate voltage-gated conductance changes of biological ion channels [128]. Here, a gate-induced transmembrane potential across the lamellar MXene nanochannels sets the dynamic threshold by creating ion depletion or accumulation zones, while engineered structural asymmetry enables unipolar or ambipolar switching that yields selective excitation and inhibition. The resulting high on/off ratio (up to 2000) and reduced subthreshold swing (560 mV decade^−1^) facilitate biomimetic spike processing, logic operations, and competitive learning in ion-based neuromorphic arrays.

**Fig. 8:**
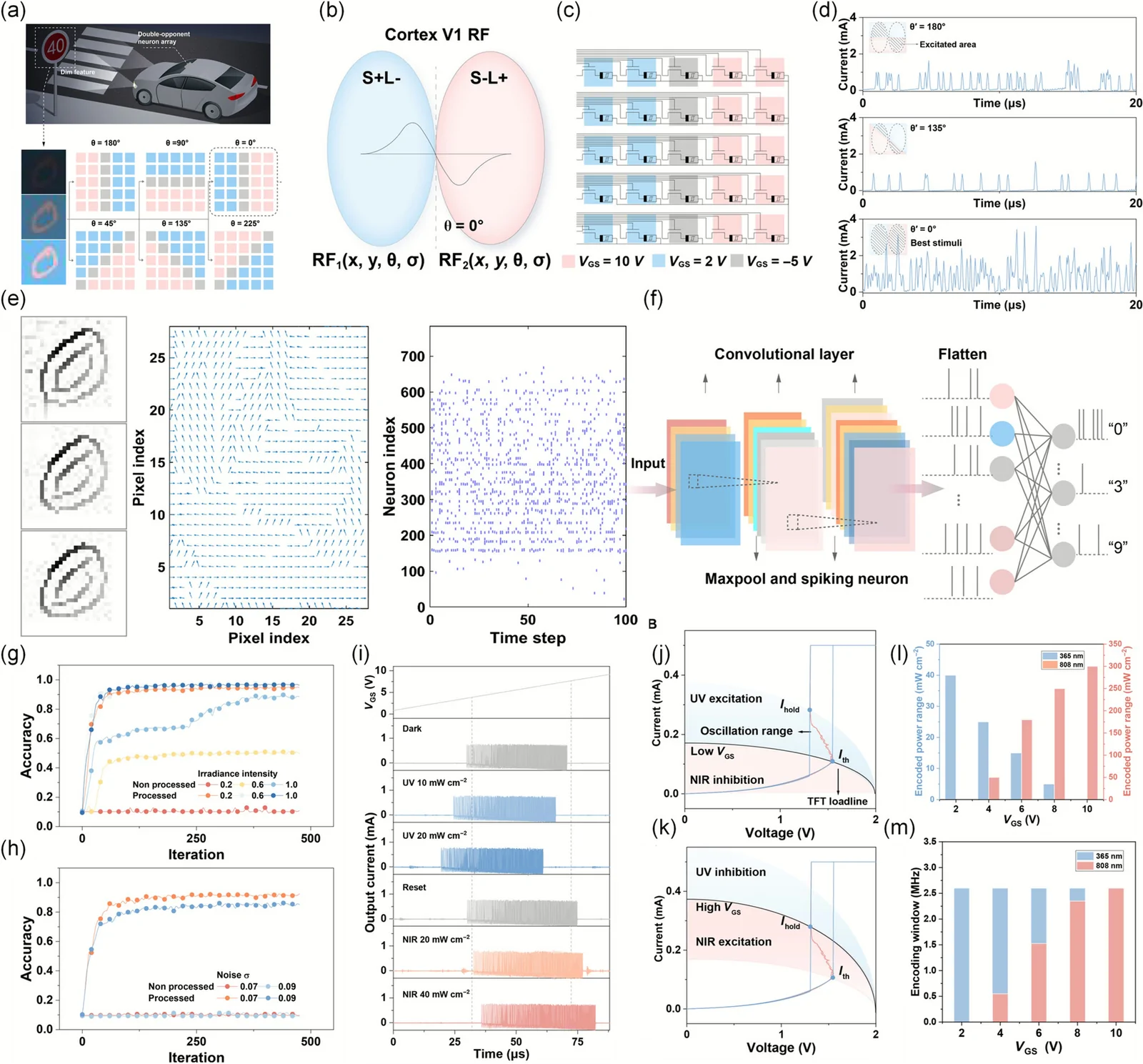
Dynamically tunable threshold plasticity and metaplasticity in neuromorphic neuron arrays**. a** Low-light driving scenario demonstrating enhanced object detection via double-opponent receptive field (DO RF) preprocessing. **b** Biomimetic DO RF structure with spatial-chromatic antagonism. **c** Gate-voltage-tunable neuron array for orientation-selective edge detection. **d** Direction-dependent spiking outputs under angular color stimuli. **e** Hardware-processed edge images and raster plots under varied illumination. **f** Spike-encoded outputs preserve features at all brightness levels. **g** Classification accuracy of SNNs for preprocessed data. **h** Preprocessing accuracy under high noise. **i** Output current evolution across operational regimes as gate voltage (V_GS_) varies. **j** UV-induced spiking and NIR inhibition at low V_GS_. **k** NIR-induced spiking and UV inhibition at high V_GS_, enabling double-opponent color coding. **l** Gate-controlled encoding range for UV and NIR stimuli. **m** Spiking frequency tunable range. **a-m** Reproduced with permission [43]. Copyright 2025, The American Association for the Advancement of Science

Building on the landscape of adaptive threshold modulation and metaplasticity, Li et al. present a pioneering artificial neuron array that unites dynamic threshold tuning with biologically inspired double-opponent receptive field coding at the hardware level [46]. By enabling in situ orientation selectivity and flexible color opponency, this system achieves robust, spike-based feature extraction and preprocessing even under low-light or noisy conditions, directly enhancing the performance and environmental adaptability of downstream neuromorphic networks. Figure 8a frames the real-world challenge by illustrating an autonomous driving scenario under low-light conditions, where traditional vision systems experience substantial recognition failures. The introduction of the artificial neuron array, capable of double-opponent receptive field (DO RF) preprocessing, demonstrates a marked restoration of object detection through the extraction of robust NIR and UV boundaries. Moving to the core mechanism, Fig. 8b visualizes the biomimetic, elliptical DO receptive field inspired by the visual cortex, whose spatial and chromatic antagonism is mathematically described by a two-dimensional Gaussian derivative, providing the basis for enhanced edge selectivity.

Device-level innovation is captured in Fig. 8c, where selective gate voltage tuning (*V*_GS_ at 2 V, 10 V, or − 5 V) configures the neuron array to respond maximally only to edges that align with the preferred orientation, achieving hardware-level context adaptation. This selectivity is substantiated in Fig. 8d, which quantifies the spiking output across input angles and shows a distinct peak only when the input matches the preset orientation, thus validating the direction-dependent coding. Figure 8e further demonstrates the practical impact of this hardware preprocessing: after passing through the array, image edges are sharply delineated across all brightness levels, as confirmed by inverted pixel images and orientation-labeled raster plots. Complementing this, Fig. 8f showcases the spike-encoded images under varying illumination, highlighting the system’s ability to maintain feature clarity for downstream computation. System-level advantages are then revealed in Fig. 8g, where preprocessed datasets fed into a convolutional SNN maintain high (> 90%) classification accuracy regardless of lighting, while non-preprocessed data see a drastic decline. This robustness is echoed in Fig. 8h, which shows that the preprocessed images consistently sustain around 80% accuracy even under heavy noise, a stark contrast to the near-complete degradation of unprocessed data. The underlying device dynamics are elucidated in Fig. 8i, mapping the evolution of output current across three operational regimes as V_GS_ is varied. Figure 8j, k deepens the picture by illustrating how UV and NIR inputs can be flexibly assigned as excitatory or inhibitory through precise V_GS_ modulation, thereby enabling biologically inspired, hardware-level double-opponent color processing. Figure 8l presents how the encoding range for each stimulus shifts as V_GS_ changes, and Fig. 8m underscores the extensive frequency tunability of the neuron’s spiking response, spanning from sub-Hz to 2.6 MHz.

Adaptive threshold dynamics operate as a plasticity engineering strategy that advances neural precision and temporal learning. Devices with tunable thresholds and metaplastic baselines regulate sensitivity and effective learning rate in real time, enabling symmetric weight updates, selective noise suppression, and timing alignment for sequence encoding. Across vacancy engineered heterojunctions, hydrogen gradient sensitized neurons, transmembrane-potential-gated ionic channels with designed asymmetry, and gate biased neuron arrays, thresholds remain stable yet history dependent at low voltage.

## Synaptic Engineering for Resource-Efficient Integration

Building truly adaptive and efficient neuromorphic systems requires not only advanced synaptic plasticity, but also hardware strategies that enable the fusion, compression, and modular integration of multiple sensory and computational functions [129, 130]. In contrast to conventional architectures, where sensing, memory, and logic remain physically separated, next-generation neuromorphic hardware must achieve direct, in-device integration and reconfigurable coupling of heterogeneous modalities, mirroring the cross-modal connectivity and parallel processing of biological neural systems [131]. This technological evolution promises to dramatically reduce hardware redundancy and energy consumption, while enhancing environmental adaptability and system-level intelligence [132].

### Device-Level Multimodal Fusion

Device-level multimodal fusion jointly encodes optical, mechanical, chemical, and environmental cues, including gas composition, temperature, and humidity, within a single, programmable synaptic pathway in the device front end. In synaptic transistors, multimodal inputs modulate the shared internal state of channel carrier density via spectral photogating with vacancy photoionization, strain or pressure induced modulation of contact or heterojunction barriers, surface redox and chemisorption, proton migration in hygroscopic dielectrics, and thermally assisted carrier or ion transport [133–138]. Writing multiple cues into the same synaptic state renders the update magnitude and polarity context dependent, enabling on-device gating and weighting that improves per-modality selectivity, extends effective dynamic range [50]. Signal compression at the device front end reduces energy and area and improves resilience to variations in illumination, temperature, and humidity [53]. Compared with single-modality synapses that sense one stimulus, multimodal synaptic transistors co-register cues within the channel and junction, enabling lighter readouts and more stable performance across diverse scenes [50]. In response, the latest generation of multimodal synaptic devices exemplifies a transformative strategy, compressing sensory input channels, fusing heterogeneous stimuli at the device level, and reducing the neural network parameter load at the system front end [139]. This allows not only a drastic reduction in sensor count and network size but also enhanced resilience and generalization in real-world complex scenarios.

A suite of representative works demonstrates the breadth and value of this approach. For instance, VP/MXene heterostructures combine photogating with adsorption-driven surface-potential tuning to achieve synergistic UV-gas dual-mode perception, enabling reconfigurable synaptic weights and dynamic memory adaptation to ambient changes; this balance between retention and selective forgetting supports adaptive perception [140]. Pentacene/P(VDF-TrFE)/Cs_2_AgBiBr_6_ hybrid systems couple optical and humidity cues at the device level, enabling real-time modulation of synaptic plasticity and memory preservation, which is directly applicable to emotion-state memory or adaptive environmental encoding [141]. Monolayer vacancy-induced oxidized (VO)-MXene-based synapses integrate visual and respiratory (humidity) stimuli, using dual channels for state-dependent weighting and emotional memory transitions, effectively mimicking context-dependent behavioral switching [142]. Olfactory-inspired in-sensor organic electrochemical transistors (OI-OECTs) consolidate chemical sensing, logic processing, and memory storage, dynamically switching between short- and long-term plasticity under varying gas concentrations, thus realizing ultra-low-power and robust chemical event detection [143]. These strategies collectively advance multimodal synaptic hardware toward generalized, resilient, and highly efficient AI perception systems, breaking the constraints of traditional, single-modality designs and pointing the way for real-world, edge-deployable intelligent interfaces.

**Fig. 9:**
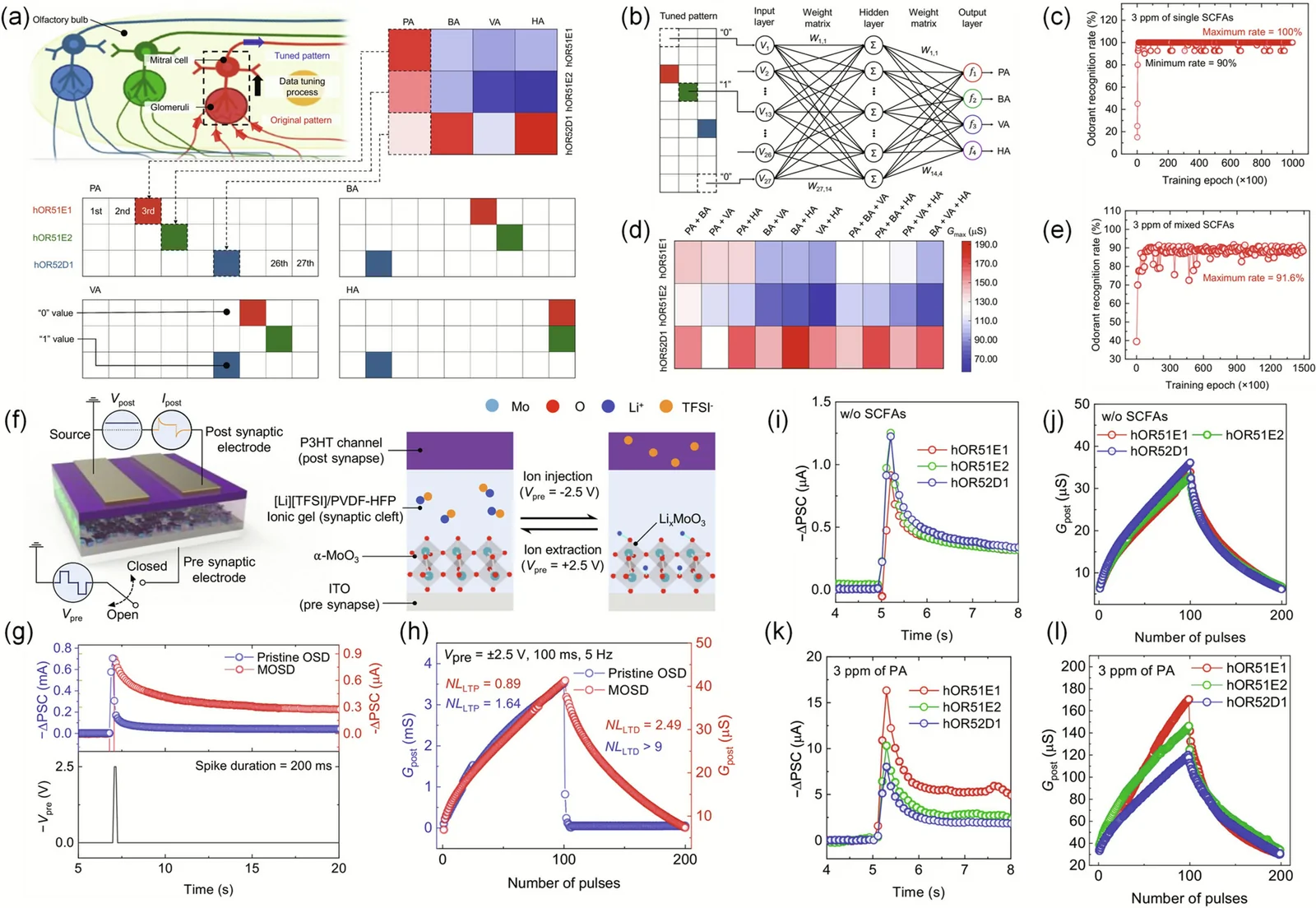
Biomimetic multimodal synaptic olfactory system and device-level characterization. **a** Schematic of data encoding in the artificial glomerulus-mitral cell network, mapping odorant stimuli into distinct conductance patterns. **b** Artificial neural network (ANN) for high-accuracy odor recognition. **c** Single-odorant recognition accuracy after training. **d** Conductance patterns and **e** recognition accuracy for odorant mixtures, validating combinatorial coding and robust inference. **f** Structure and operating principle of the MoO_3_-organic synaptic device (MOSD) with programmable redox modulation. **g** EPSC memory retention in MOSD versus pristine device. **h** Linearity and symmetry of LTP/D in MOSD versus pristine device. **i** EPSC and **j** LTP/D in AOSs without short-chain fatty acids (SCFAs). **k** EPSC and **l** LTP/D responses under PA exposure. **a-l** Reproduced with permission [44]. Copyright 2024, The American Association for the Advancement of Science

Within this technological landscape, the artificial olfactory system (AOS) proposed by Song et al., which integrates human olfactory receptor nanodisks (hOR NDs) with a redox-active MoO_3_-functionalized organic synaptic device (MOSD), marks a distinctive advance in molecular specificity and signal processing precision [47]. Drawing inspiration from the glomerulus and mitral cell hierarchy in the biological olfactory bulb, the AOS translates short-chain fatty acids (SCFAs)-induced conductance into high-dimensional 9 × 3 arrays (Fig. 9a), which are mapped onto a custom artificial neural network (ANN) (27 input, 14 hidden, 4 output neurons; Fig. 9b). This biomimetic refinement enables the system to rapidly achieve 100% recognition accuracy for single odorants (Fig. 9c), and accurately discriminate odorant mixtures via combinatorial conductance patterning and ANN inference (Fig. 9d, e). At the hardware level, the MOSD exploits programmable lithium ions (Li^+^)/ bis(trifluoromethanesulfonyl)imide (TFSI^−^) redox intercalation for stable and linear weight updates (Fig. 9f), yielding dramatically enhanced EPSC memory (∼700 s versus 5 s for pristine OSD; Fig. 9g) and minimized LTP/D nonlinearity (Fig. 9h). The hybrid AOS platform demonstrates hOR-specific conductance responses, remaining stable in the absence of odor (Fig. 9i, j) and providing sensitive, type-dependent readouts under SCFA exposure, with detection limits as low as 0.07 ppm (Fig. 9k, l). Principal component and fluorescence analyses confirm the molecular selectivity and reliability of odor recognition.

Device-level multimodal fusion co-encodes heterogeneous sensory cues in a single synaptic pathway to deliver resource-efficient, noise-robust, context-aware perception. Optical, mechanical, chemical, and environmental inputs modulate a shared channel carrier density through photogating, chemisorption-driven surface potential shifts, and ionic transport, producing context-dependent thresholds and weight updates that increase selectivity, widen dynamic range, suppress cross-sensitivity, and compress data at the source. Heterojunction stacks, MXene lamellae, peptide nanowires, and redox-active electrolytes stabilize interfaces and support reversible storage, enabling linear, low-variance updates. Prioritizing front-end fusion improves accuracy with fewer sensors and lighter readouts, advancing neuromorphic olfaction and vision at the edge.

### Single-Device Functional Integration

The pursuit of low-power, dense, and adaptable neuromorphic hardware has shifted attention to single-device functional integration, where sensing, memory, and elementary computation are implemented within one physical element operating under a single stimulus modality [144, 145]. This focus is distinct from multimodal integration, which fuses two or more physical modalities into a shared pathway. Here, emphasis is placed on single-device multifunctionality realized within one device. This direction targets the energy cost of disaggregated sensing-memory-compute chains whose split data flows inflate power at the edge, in wearables, and in Internet of Things (IoT) devices [146]. Current design, therefore, concentrates on compressing neural primitives into a single device through materials and architectural engineering, together with bias-programmable operating modes. By consolidating roles locally, these devices reduce peripheral circuitry and data movement, improve energy efficiency and area utilization, and do so while preserving analog linearity, symmetry, and endurance required for reliable learning [147].

**Fig. 10:**
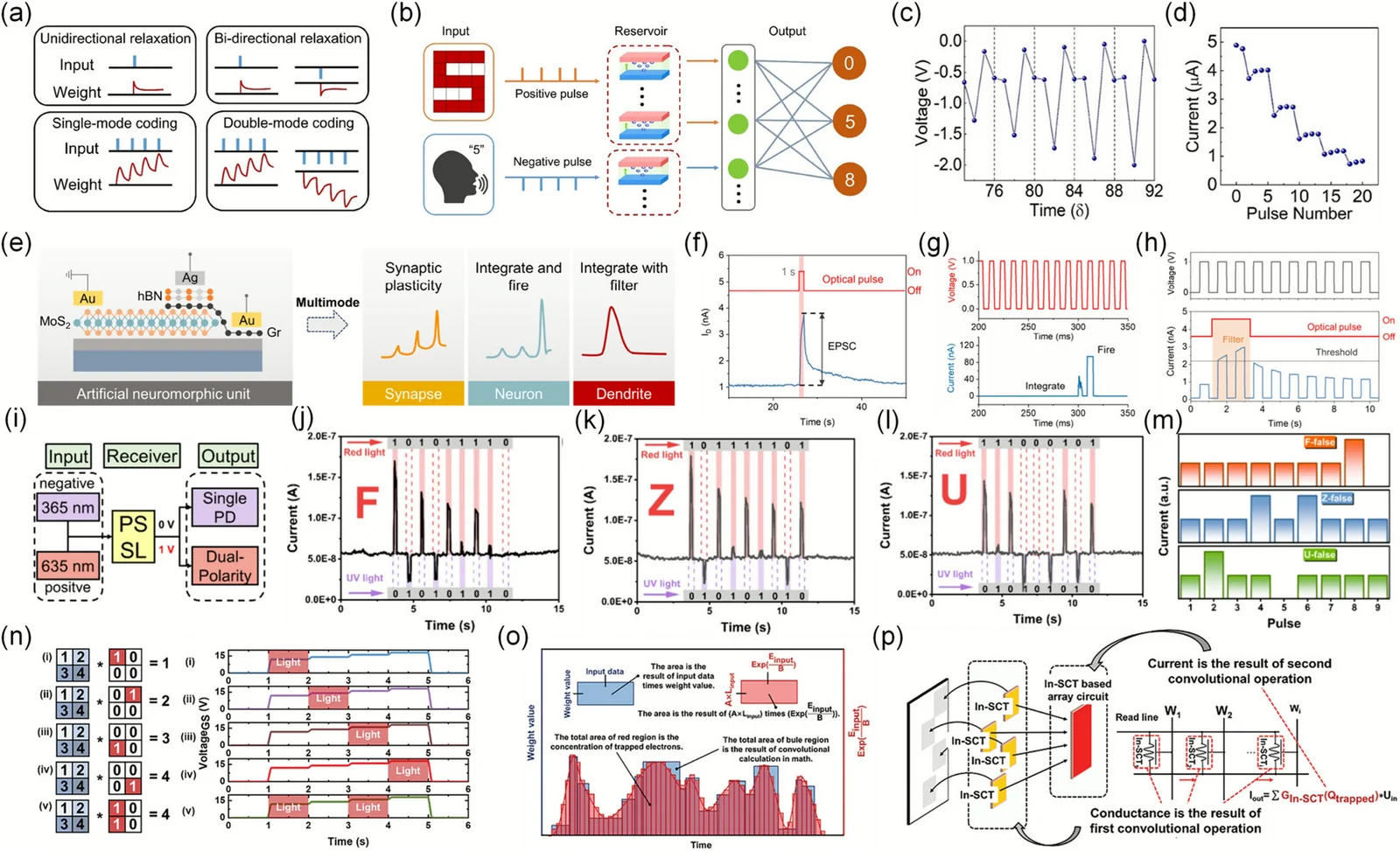
Multifunctional and multimodal single-device neuromorphic platforms. **a** Schematic of unidirectional and bidirectional ferroelectric tunnel junction (FTJ) synapses for single- and dual-mode signal coding. **b** Multimodal digit recognition system: positive pulses encode images, negative pulses encode speech, with the cochlea-gram illustrating audio processing. **c** Input negative voltage sequence for voice encoding. **d** Experimental current response of the FTJ array during multimodal digit recognition. **a-d** Reproduced with permission [130]. Copyright 2025, Wiley–VCH GmbH. **e** Structure of reconfigurable MoS_2_/hBN/graphene neuromorphic units integrating synaptic, neuronal, and dendritic functions. **f** EPSC triggered by optical pulse. **g** Integrate-and-fire response of the artificial neuron under voltage pulses. **h** Dendritic current response filtered by light. **e–h** Reproduced with permission [45]. Copyright 2024, American Chemical Society. **i** Three-mode photosensitive synaptic LED (PSSL) enabling programmable photodetection, logic, and synaptic light emission. **j-l** Encrypted light pulse information for different letters received by the bipolar photodetector mode. **m** Error pulse information received by the conventional unipolar light detector. **i-m** Reproduced with permission [131]. Copyright 2024, American Chemical Society. **n** Schematic of in situ convolutional transistor (In-SCT), showing input stimuli and conductance changes. **o** Device-level correspondence between conductance area and convolution result. **p** In-SCT array with stacked convolution layers for hierarchical in-memory computing. **n-p** Reproduced with permission [133]. Copyright 2024, Wiley–VCH GmbH

A particularly striking electrical approach is exemplified by the ferroelectric tunnel junction (FTJ) synapse proposed by Nie et al., whose true significance lies in its ability to unify volatile and nonvolatile memory dynamics for robust, hardware-level sensor fusion [148]. The FTJ architecture (Fig. 10a) intricately combines ferroelectric polarization with oxygen vacancy migration, producing a device simultaneously capable of high-density, nonvolatile storage and fast, adaptive relaxation. This architecture enables dual-input fusion, as spatially divided array regions (Fig. 10b) encode image and speech modalities through positive and negative pulses, respectively, a direct hardware analog to biological multisensory convergence. Figure 10c details pixel-wise voltage pulse encoding, and Fig. 10d presents the reservoir's experimentally resolved current outputs during digit recognition, where clear multimodal state separation is observed. The seamless fusion of logic and adaptive memory in this design echoes the functional plasticity of neural circuits, underscoring a decisive leap beyond conventional, unimodal synaptic emulators.

The theme of integrated neural computation is further advanced by the reconfigurable MoS_2_/hBN/graphene neuromorphic unit introduced by Hu et al. [48]. This platform uniquely orchestrates synaptic, neuronal, and dendritic behaviors within a compact 2D heterostructure, enabling mode switching through selective biasing and multiterminal configuration (Fig. 10e). The device’s optoelectronic synaptic plasticity is elegantly demonstrated in Fig. 10f, while Fig. 10g captures its neuron-like integrate-and-fire response, essential for spiking neural network emulation. The capacity for hardware-level dendritic filtering and nonlinear logic is evidenced in Fig. 10h, where light-modulated currents realize both passive and active dendritic computations. This versatile architectural motif sets a new benchmark for the emulation of higher-order brain functions in hardware, with the potential to underpin densely reconfigurable, scalable neuromorphic systems.

In the realm of optoelectronic multifunctionality, the three-mode photosensitive synaptic LED (PSSL) platform offers an integrated solution for sensing, preprocessing, and logic-level information security [149]. Its device-level voltage programmability (Fig. 10i) permits seamless toggling among broadband photodetection, dual-polarity logic, and synaptic light emission, thus enabling both robust signal discrimination and neuromorphic processing within a single unit. The encoding of patterns into orthogonal red/UV optical channels is visualized in Fig. 10j, while the superposition and resultant cancelation of dual-wavelength signals for logic operations are shown in Fig. 10k, l. Most notably, Fig. 10m highlights that under simultaneous red and UV illumination, the bipolar photodetector outputs “0” to suppress interference, whereas a conventional device gives “1” and yields decoding errors (“111 111 110”). This highlights the PSSL's inherent superiority for secure, high-fidelity optical communication, where simple device-level operations achieve reliable encryption without algorithmic overhead. In addition, Wu et al. developed ferroelectric-defined reconfigurable MoTe_2_ homojunctions using a P(VDF-TrFE) split gate dielectric to program local ferroelectric domains for tunable p-n/n-p junctions and analog weight storage [150]. The devices exhibited 17 stable positive and negative photoresponsivity states with long retention, and when assembled into a 3 × 3 array, they enabled concurrent sensing, memory, and computing, thereby realizing true single-device functional integration for low-power neuromorphic vision systems.

When it comes to in situ convolution computation, the in situ convolutional transistor (In-SCT) represents a watershed moment by merging analog convolution and synaptic memory within a single hardware node [151]. Its operational mechanism, based on dynamic carrier trapping and optoelectronic modulation, is outlined in Fig. 10n. More compellingly, Fig. 10o visualizes the mathematical equivalence between device conductance change and convolution area, a striking correspondence between device physics and algorithmic logic. Figure 10p extends this logic to demonstrate hierarchical stacking of convolutional operations within a single device/circuit, hinting at a future where memory and computation are inseparable at the hardware level. This approach dramatically reduces network area and power consumption, paving the way for ultra-compact, energy-efficient CNN implementations.

Beyond these, environmentally adaptive and spectrum-compressed synaptic devices contribute new dimensions of flexibility and task specificity. The In_2_O_3_·SnO_2_/Nb:SrTiO_3_ (ITO/NSTO) heterojunction optoelectronic synapse, for example, exemplifies the integration of in-sensor multimodal perception and real-time computation in a minimalist two-terminal design, foundational for robust temporal pattern recognition and low-latency AI in complex environments [152]. Organic memristors such as polymer switching material with triphenylamine and azobenzene (PFTPA-AZO) demonstrate the utility of molecularly engineered dual-mode operation, bridging fast in-sensor feature extraction and deep learning in memory, while Ag/ZnO_x_/TiO_y_/ITO environmental memristors reveal how physical responsiveness to electrical, thermal, and humidity cues can endow hardware with the context awareness and adaptability reminiscent of biological systems [153, 154]. The dual-mode silicon-on-insulator (SOI)/graphene photodetector, mimicking retinal cone-rod switching, achieves an ultrawide dynamic range, providing a resilient solution for vision under fluctuating illumination, a perennial challenge in autonomous perception [155]. Finally, flexible PbS QDs/polymethyl methacrylate (PMMA)-pentacene synaptic transistors, with broadband photoresponse and gate-tunable analog memory, provide in-sensor denoising and contrast normalization that reduces front-end parameter overhead and conversion/traffic burden, yielding more noise-robust classification for wearable, conformable vision systems [29].

Single-device functional integration concentrates sensing, memory, and elementary computation in one element under a single modality, reducing peripheral circuitry and data movement. By co-harvesting volatile and nonvolatile pathways in one stack, optoelectronic transduction with emissive readout, and bias-programmed operating regimes, the channel or junction serves as a unified state variable reconfigurable for sensing, plasticity, neuron-like integration, and logic with local preprocessing and storage. Ferroelectric domain engineering, vacancy control, split gate dielectrics, laminar two-dimensional heterostructures, and redox reservoirs stabilize interfaces, enable low-voltage operation, and sustain linear, low-variance updates. Functionally, selective spectral gain, programmable persistence, conductance-mapped analog convolution, and compact multiterminal layouts for dendritic filtering and spiking move computation to the source, lowering energy and area while preserving accuracy.

### Multidevice Modular Integration

Multidevice modular integration refers to the co-design of locally coupled, functionally distinct blocks, for example, sensors, synaptic elements, neuron circuits, lightweight readouts, and actuators, into a resource-efficient module [156]. Emphasis is on co-location and interface alignment by matching bias ranges, conductance windows, and time constants, so sensing and preprocessing occur in the sensor, plasticity updates run near memory, and computing occurs [6]. This approach reduces conversion and interconnect overhead, improves task-level trade-offs among accuracy, latency, and energy, and scales by replicating standardized modules rather than enlarging monolithic systems [157]. This section, therefore, examines architectural patterns and device choices that support robust coupling, including sensors matched to synaptic dynamic range, synapses and neurons with aligned time constants, and memory and readout paths that preserve analog fidelity while remaining interoperable with digital controllers, enabling resource compact neuromorphic subsystems that are ready for deployment.

**Fig. 11:**
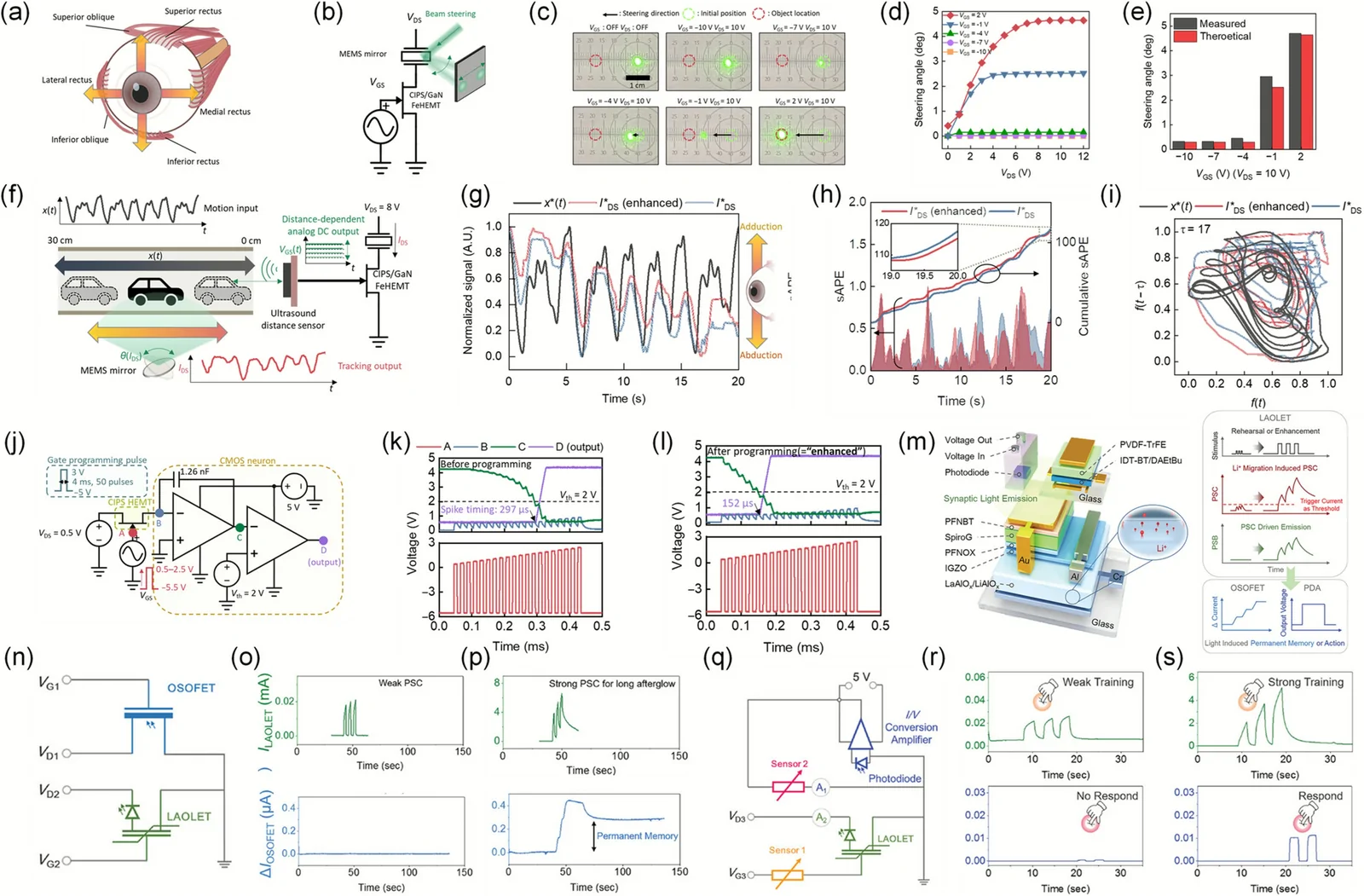
Modular multidevice units for physical functional integration. **a** Schematic of the biological oculomotor system. **b** Microelectromechanical system (MEMS) mirror actuation by CuInP_2_S_6_ (CIPS)/GaN ferroelectric high-electron mobility transistor (FeHEMT). **c** Laser displacement versus FeHEMT output. **d** Proportional control of steering angle by FeHEMT gate voltage. **e** Measured versus theoretical steering angles. **f** Ultrasound distance sensor enables object tracking. **g** Time-resolved tracking output. **h** Tracking absolute error (sAPE) analysis before and after synaptic enhancement. **i** Phase plot of motion input and output. **j** Schematic of FeHEMT-integrate-fire unit (IFU) integration for spike-based computation. Output spike latency **k** before and **l** after synaptic programming; enhancement halves response time. Reproduced with permission [140]. Copyright 2023, The American Association for the Advancement of Science. **m** Structure of memory optocoupler module for threshold-driven, light-mediated signal transfer. **n** Circuit diagram of neurotransmitter transfer memory optocoupler. Real-time optocoupler output under **o** subthreshold and **p** suprathreshold input. **q** Schematic of PSC-triggered action memory optocoupler integrated with tactile sensors. Device responses under** r** weak and** s** strong training. Reproduced with permission [46]. Copyright 2024, The Authors. Advanced Materials published by Wiley‐VCH GmbH

A key exemplar of heterogeneous integration is the CuInP_2_S_6_ (CIPS)/GaN ferroelectric high-electron mobility transistor (FeHEMT)-based artificial neuromuscular junction (NMJ) module, which physically unites synaptic plasticity, high-power actuation, environmental sensing, and hardware learning within a single closed loop [158]. As illustrated in Fig. 11a, the architecture draws direct inspiration from biological oculomotor systems, depicting the extraocular muscles responsible for adduction and abduction of the eyeball. Figure 11b details the system-level integration, where a microelectromechanical system (MEMS) mirror is directly driven by the FeHEMT, establishing amplifier-free, milliamperes-level actuation. The laser beam displacement, as mapped in Fig. 11c, visually confirms the precise, voltage-controlled mechanical steering achieved by the integrated module. Figure 11d quantitatively presents the relationship between gate voltage and steering angle, demonstrating proportional, analog control of the actuator via synaptic modulation. Figure 11e provides a critical benchmark, contrasting experimental steering angles with theoretical predictions and affirming the fidelity of this direct synapse-actuator pathway. Integration of real-time sensory feedback is achieved in Fig. 11f, where an ultrasound sensor is interfaced to the FeHEMT gate, enabling in situ distance measurement and conversion of environmental position data into actuation commands. Figure 11g documents the time-resolved tracking of a moving object: the FeHEMT’s output current dynamically modulates the MEMS mirror in response to positional inputs, allowing the system to track the trajectory of a mobile robot. Figure 11h analyzes tracking accuracy by plotting the symmetric absolute percentage error (sAPE) over time, revealing a significant error reduction after synaptic enhancement, a result of plasticity-induced transconductance gains. Figure 11i presents the phase relationship between input and output signals, showing that postenhancement, the system achieves closer temporal and amplitude matching, indicative of hardware-level learning and adaptive feedback. Figure 11j introduces the system’s ability to interface with a CMOS-based integrate-and-fire unit (IFU), depicting the circuit architecture for temporal spike integration and threshold-based firing. Figure 11k, l compares the output spike response before and after synaptic enhancement; the programmed state yields a marked reduction in output latency (from 297 to 152 μs), mirroring the accelerated reflexes observed in biological systems.

Moving to optoelectronic modules, Fig. 11m shows the memory optocoupler’s structural design: a long afterglow organic light-emitting transistors (LAOLET), an organic field-effect transistor (OSOFET), and a photodiode amplifier (PDA), configured for threshold-gated, light-driven information transfer [49]. Figure 11n diagrams the neurotransmitter transfer memory mechanism, wherein synaptic light emission from the LAOLET induces nonvolatile photomemory in the OSOFET, establishing a photonic bridge for inter-device signaling. Figure 11o records real-time device behavior under subthreshold stimulation, documenting the generation and rapid decay of volatile postsynaptic currents. By contrast, Fig. 11p captures the system response to suprathreshold input: the LAOLET’s long afterglow triggers persistent photomemory in the OSOFET, enabling the transition from transient to permanent memory encoding. To support logic-level operation, Fig. 11q presents the equivalent circuit of the memory optocoupler integrated with tactile sensors for multimodal training and reaction. Figure 11r describes a weak training scenario, wherein repeated low-voltage pressure input fails to elicit a downstream response due to insufficient postsynaptic current (PSC). Figure 11s details the outcome of strong training, where elevated PSCs activate sustained light emission and drive the PDA, resulting in a measurable current response to tactile stimulus, thus demonstrating device-level implementation of sensory learning, threshold gating, and logic-controlled action.

Complementing closed-loop sensor-actuator and memory-decision modules, other modes of heterointegration further broaden the horizons of neuromorphic hardware. The hemispherical Ag-TiO_2_ nanocluster optoelectronic memristor array embodies geometry-aware integration, leveraging a curved substrate to achieve a wide field-of-view, in-sensor visual processing, and real-time binocular depth perception [159]. This platform unites all-optical synaptic modulation, spatial angle encoding, and event-driven computation within a single, conformable array, effectively emulating advanced retinal functions and supporting intelligent visual tasks unattainable by planar or unimodal devices. In parallel, van der Waals one-transistor–one-ferroelectric-memristor (1T1M) hybrid architectures exemplify device-level memory-logic co-integration. By combining multilevel ferroelectric switching with transistor gating, these structures overcome sneak-path and crosstalk bottlenecks, enabling robust, low-power in-memory computing and supporting hardware arithmetic alongside synaptic modulation [160]. Extending to macroscopic scales, fiber-based iontronic synapse networks enable distributed, programmable perception-actuation in smart textiles, illustrating how spatially and functionally distinct modules can be orchestrated to deliver adaptive, high-density neuromorphic systems [161]. Together, these complementary strategies highlight the critical importance of heterointegration as the foundation for resource-efficient, robust, and scalable AI.

Across multidevice modular integration, the goal is to co-design locally coupled heterogeneous blocks into resource-efficient neuromorphic modules that improve task accuracy, latency, and energy. Keeping sensing and preprocessing at the source, placing plasticity and matrix operations near memory, and using sparse event-driven links between modules reduces conversion and interconnect overhead while preserving analog fidelity. At the hardware level, options such as ferroelectric domains on wide bandgap channels, redox-active interlayers for photonic memory coupling, van der Waals 1T1M stacks with controlled contacts, hemispherical substrates for wide field mapping, and fiber-based ionic conduits stabilize interfaces, limit crosstalk and sneak paths, and sustain linear, low-variance updates, providing a practical route to deployable modular systems.

## Synaptic Engineering for Scalable Arrays

Scaling from single synaptic devices to brain-inspired arrays exposes challenges that differ fundamentally from device-level studies. At the device statistics level, device-to-device variability, cycle-to-cycle stochasticity, update nonlinearity and asymmetry, limited state count, retention drift, and endurance spread accumulate over thousands to millions of cells and directly erode learning accuracy and convergence [162]. At the array physics level, passive crossbars suffer from sneak currents and half-select disturb, while line resistance and capacitance introduce voltage drop and timing skew; moving to one transistor one resistor (1T1R) mitigates sneak paths but imposes leakage, and matching constraints [163–165]. At the process and integration level, wafer-scale uniformity and back-end-of-line (BEOL)-compatible thermal budgets govern yield for ferroelectric and 2D stacks; interconnect resistance and 3D stacking add further constraints, and postbonding stress can degrade memory windows, making packaging and encapsulation critical for optoelectronic or environment-responsive and flexible arrays [166, 167].

Against this backdrop, the advance of intelligent and energy-efficient edge computing relies on synaptic arrays and integrated systems that offer scalable uniformity, multifunctional adaptability, and seamless links between sensing, computation, and action [168, 169]. Recent progress in high-uniformity single-device arrays, multifunctional heterostructures, and context-adaptive in-sensor computation enables robust memory encoding, dynamic plasticity, and efficient signal processing at both the pixel and system level [170]. Integrating multimodal sensing, memory, and logic within single devices and arrays now supports real-time cross-modal recognition and adaptive decision-making, while heterogeneous integration of memory, logic, and sensory modules enables closed-loop, low-power operation essential for IoT and autonomous applications. Together, these developments establish the foundation for intelligent, flexible, and resource-efficient neuromorphic hardware at the edge [171, 172].

### High-Uniformity Arrays

Achieving high uniformity and scalability in synaptic arrays is critical for translating synaptic plasticity to robust neuromorphic systems [173]. At the array scale, uniform device statistics yield predictable pulse-to-weight transfer functions, consistent LTP/LTD kernels, and stable read margins, which in turn enable direct weight mapping from software models, reduce per-cell calibration, and improve matrix–vector accuracy under line parasitics and temperature drift. Device-to-device variability, nonlinearity, and fabrication defects hinder the mapping of plastic behaviors across large arrays. Emphasizing array-level uniformity shifts the focus from isolated device figures to system outcomes: higher mapping yield, consistent learning rules across tiles, lower programming time and peripheral energy, and improved reliability over long deployments. Recent strategies across material platforms have tackled these challenges effectively. For instance, a 28 × 28 floating-gate transistor array based on monolayer MoS_2_ with Au nanoparticle charge-trap layers demonstrated excellent uniformity: on–off ratios around 10^6^ and mobility averaging ~ 8 cm^2^ V^−1^ s^−1^ across 784 devices, permitting a single global programming schedule and straightforward weight transfer, which enabled optoelectronic handwriting encoding and achieving ~ 96.5% accuracy in digit recognition [174]. Likewise, fully screen‑printed, paper-based ZnO synaptic transistor arrays fabricated via low-temperature printing achieved large-area uniformity, environmental stability, and biodegradability. The tight row-to-row behavior supported uniform pulse schemes and stable read margins, enabling photoelectric synaptic behaviors such as paired-pulse facilitation and filtering, and achieved 91.4% recognition accuracy with ~ 3.7 pJ per synaptic event [175].

**Fig. 12:**
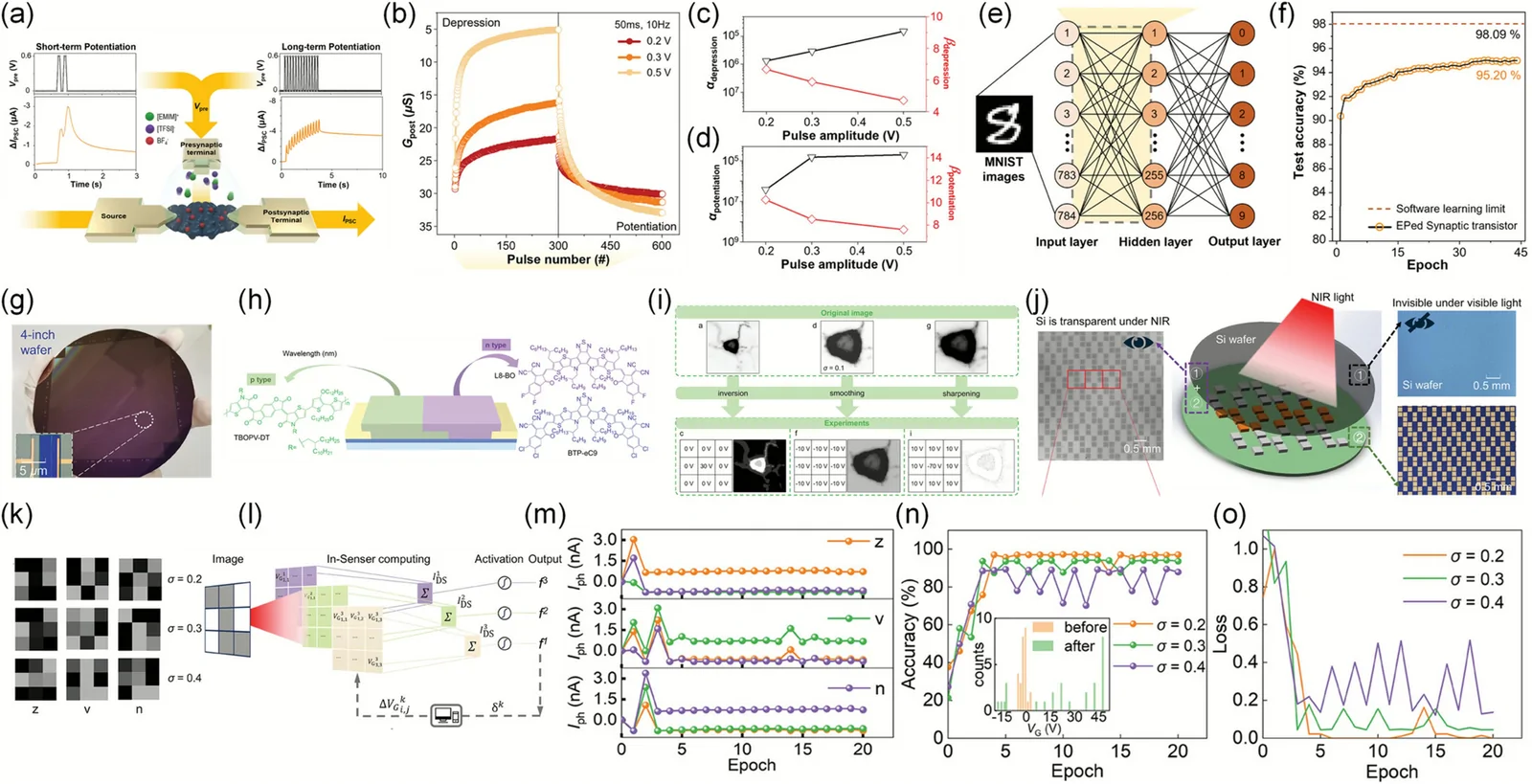
Scalable single-device synaptic arrays and multifunctional organic heterostructure arrays for neuromorphic hardware. **a** Schematic and operation mechanism of poly(3,4-ethylenedioxythiophene) (PEDOT):tetraborofluoroate (BF_4_) synaptic transistor. **b** LTD/P modulation under varying pulse amplitudes. **c** Nonlinearity of depression. **d** Step size of potentiation. **e** Multilayer perceptrons (MLP) architecture for MNIST using hardware synapses. **f** Training accuracy curve. **a-f** Reproduced with permission [47]. Copyright 2024, The Author. Advanced Materials Technologies published by Wiley‐VCH GmbH. **g** 4-inch wafer with 520 devices/cm^2^ organic heterostructure arrays. **h** Device structure and NIR semiconductor chemical structures. **i** Pixel-level grayscale inversion, smoothing, and edge enhancement. **j** NIR readout of letters through silicon wafers. **k** Noisy letter image dataset for classification. **l** Hardware classifier: pixel mapping and training. **m** Photocurrent outputs for letters. **n** Accuracy versus noise level. **o** Loss curve during training at different noise levels. **g-o** Reproduced with permission [157]. Copyright 2024, The Authors. Advanced Materials published by Wiley–VCH GmbH

On the organic front, while organic conductive polymers such as poly(3,4-ethylenedioxythiophene) (PEDOT) offer inherent advantages for low-cost, large-area integration, conventional approaches remain constrained by multistep patterning and variability. The one-shot integrable electropolymerization (OSIEP) method, built on alternating-direct current (ADC) bipolar electrochemistry, fundamentally resolves these issues by enabling remote, maskless, and large-area growth of PEDOT/tetraborofluoroate (BF_4_) channels on ultrathin substrates, yielding synaptic arrays with exceptional uniformity and simplified fabrication workflows [176]. Figure 12a illustrates the architecture of a three-terminal electrochemical synaptic transistor, specifically engineered to emulate the dynamic plasticity of biological synapses. Positive voltage pulses at the presynaptic terminal induce EMIM^+^ cation injection and PEDOT^+^ backbone de-doping within the channel, producing both short-term depression (STD) and LTD depending on pulse protocol. The transition from STD to LTD, manifested as persistent conductance suppression exceeding 100 s, directly mirrors the memory windows of biological synapses. Figure 12b details the LTD/LTP characteristics extracted under various pulse amplitudes, revealing how synaptic weight modulation is stimulus dependent. The extracted nonlinearity and step size parameters in Fig. 12c (depression) and 12d (potentiation) confirm that higher pulse amplitudes yield more linear and effective conductance modulation, which is critical for hardware-compatible neural learning rules. Critically for scalability, the array’s uniformity allowed the experimentally extracted LTD/LTP curves to serve as a single look-up table for weight updates across the network, avoiding per-cell fitting and ensuring consistent learning rules at scale. Moving from device to system, Fig. 12e depicts the neural network simulation architecture, a MLP designed for MNIST digit recognition, where the experimental LTD/LTP curves serve directly as weight-update profiles. The learning trajectory, as shown in Fig. 12f, demonstrates a rapid and robust ascent in test accuracy, ultimately reaching 95.2%, illustrating how array-level uniformity enables reliable weight mapping and system-level reliability, comparable to software-only training.

### Reconfigurable In-Sensor Arrays

The evolution of multifunctional and highly integrated device arrays is rapidly transforming the landscape of neuromorphic sensory hardware, enabling edge systems to break free from the energy and latency constraints inherent to traditional von Neumann architectures [177]. Organic photoelectronic devices, long celebrated for their flexibility, tunable bandgap, and biocompatibility, have shown unique potential for miniaturized, high-density vision platforms [178]. However, in most conventional organic synaptic devices, the input-to-output response is largely fixed by the material stack and processing, which limits the ability to reprogram gain, polarity, or persistence during operation; as a result, pixel-level dynamic reconfigurability, essential for intelligent sensory networks, is still difficult to achieve [179]. Here, reconfigurability is treated as plasticity. At the pixel and array levels, programming the responsivity sign and gain, the effective threshold, and the persistence window constitutes task-conditioned, higher-order plasticity that enhances neural precision and enables adaptive temporal learning. Recent efforts have focused on organic heterostructures and multifunctional device arrays that enable gate-tunable, bidirectional responses and pixel-level reconfigurability.

For example, Xu et al. realized gate‑tunable positive/negative NIR photoconductance in a photolithography‑compatible organic p-n heterostructure array (5 µm channel; ~ 520 devices cm^−2^), enabling pixel‑level filtering and in‑sensor classification with reported accuracy up to ~ 97% under NIR illumination [180]. Here, the gate acts as a metaplastic control that selects the weight sign and dynamic range per pixel, improving separability while keeping a consistent programming schedule across the array, which strengthens precision in weight mapping. Complementarily, Liu et al. demonstrated an all‑photolithography ion‑gated flexible organic transistor (OIGT) array that can be programmed between volatile and non‑volatile modes, supporting multimodal neuromorphic computing at low training cost, highlighting practical array‑level reconfigurability [181]. Switching between volatile and persistent states provides a tunable short- to long-term memory window, aligning device time constants with task dynamics and enabling temporal learning at the sensor plane.

In particular, organic p-n heterostructure arrays have demonstrated gate-tunable bidirectional photoresponses, providing a representative example of how pixel-level reconfigurability can be realized in practice [180]. As demonstrated in Fig. 12g, this approach yields a remarkable device integration density of 520 cm^−2^ on a 4-inch wafer, with each channel miniaturized to just 5 μm, showcasing not only high areal density but also full compatibility with scalable photolithography. The device’s vertical bilayer architecture (Fig. 12h), comprising a partially overlapped p-type conjugated polymer and an n-type small molecule, is engineered via orthogonal solvents to realize a robust, gate-controlled, bidirectional NIR photoresponse, thereby encoding a programmable palette of synaptic weights and memory kernels inside the array.

The practical impact of this architecture is vividly illustrated in Fig. 12i, where individual pixel responsivities are mapped linearly to their respective gate voltages, allowing direct hardware realization of core image processing functions. Grayscale inversion, Gaussian smoothing, and Laplacian edge enhancement are all performed in situ, with experimental results that closely match the outcomes of ideal digital filter kernels, demonstrating the system’s flexibility and effectiveness for analog image preprocessing at the sensor level. Relative to digital preprocessing, these analog operations avoid early quantization, reduce conversion and data movement overhead, and provide continuous-valued kernels whose cutoff and gain can be tuned in real time. Figure 12j further shows that silicon's NIR transparency enables noninvasive readout through packaged devices, which extends analog preprocessing to secure or embedded settings without disturbing the front end.

At the algorithmic and systems level, the versatility of the heterostructure array is further exemplified by its ability to function as a real-time, noise-robust hardware classifier. Here, each pixel acts as a dynamic, trainable synaptic node, with V_G_ updated by error-gradient descent to encode weights for letter image recognition tasks (“z”, “v”, “n”) under varying Gaussian noise. The convergence and learning dynamics are further detailed in Fig. 12m, which shows that high-fidelity classification is achieved after only 3–4 epochs, even for significant noise levels. Quantitative benchmarking in Fig. 12n highlights the platform’s robustness, with classification accuracy peaking at 97.06% for σ = 0.2 and the VG distribution rapidly stabilizing through training; Fig. 12o records a correspondingly rapid drop in the loss function, underscoring the network’s efficiency and learning stability. These results position reconfigurability as plasticity engineering at scale. Programming the sign, gain, and persistence of weights improves neural precision, while tuning volatility provides task-matched temporal windows. Together, these capabilities enable adaptive, low-overhead preprocessing that conventional digital preprocessing chains struggle to deliver.

### Multimodal Perception Arrays

The quest for adaptive multimodal neuromorphic sensing has driven the evolution of edge-intelligent hardware toward systems capable of real-time, high-dimensional perception and robust cross-modal learning [182, 183]. Traditional architectures, which compartmentalize sensing, memory, and computation, have proven inadequate for the demands of dynamic environments such as IoT-enabled pollution monitoring and autonomous robotics, where complexity, signal crosstalk, and nonlinearity prevail [184, 185].

Recent innovations have begun to bridge this gap. For instance, Talanti et al. developed a CMOS-integrated organic neuromorphic imager (640 × 512 pixels) capable of both frame-based imaging and synaptic-mode temporal sensing, with in-pixel memory retention over tens of minutes and hardware-level motion trajectory extraction using charge recombination dynamics [186]. Complementarily, He et al. demonstrated a multimodal electronic skin embedding organic transistors for simultaneous visual and tactile perception, achieving real‑time fusion of light and pressure signals within a compact neuromorphic array [187].

**Fig. 13:**
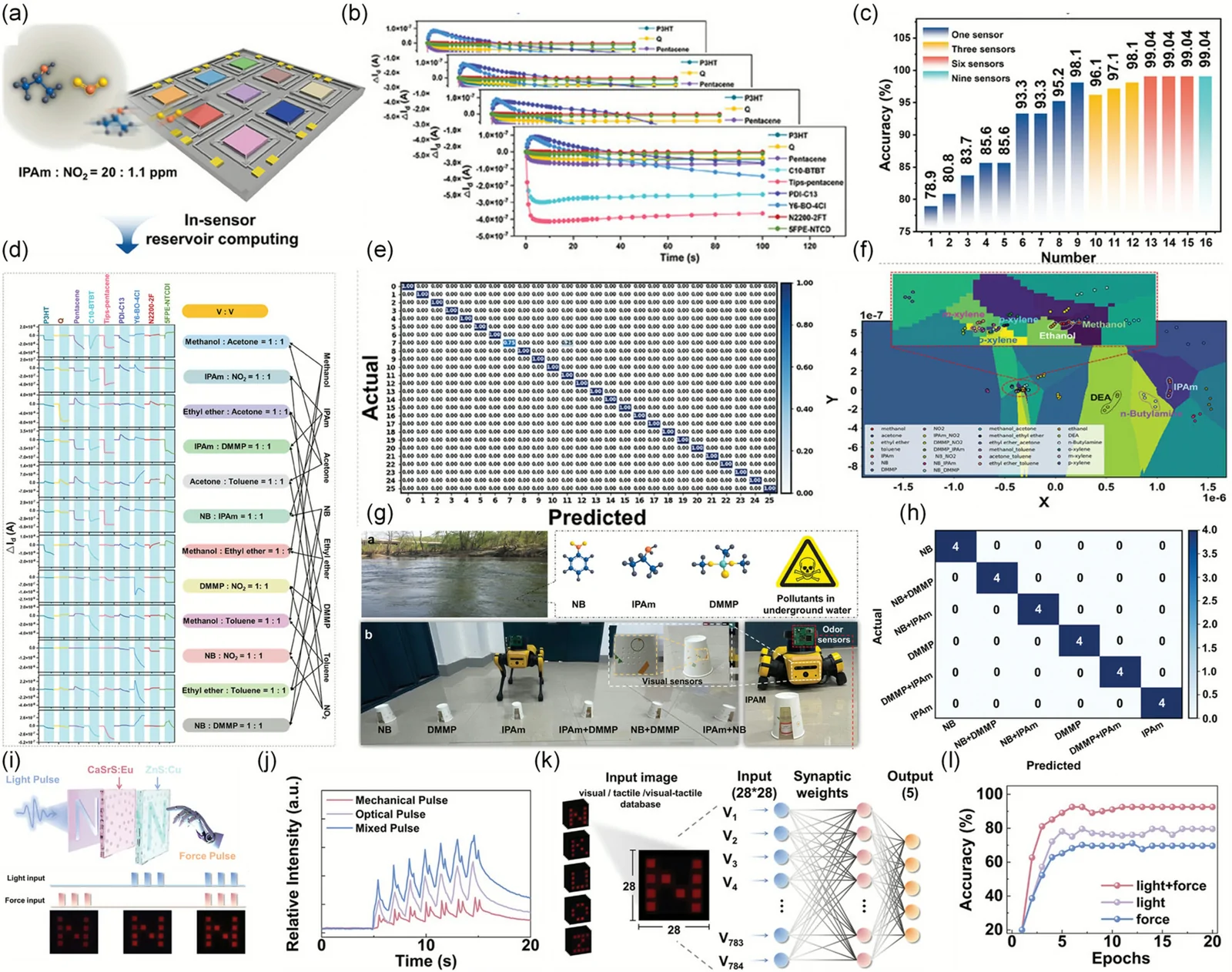
Multimodal single-device arrays for cross-modal perception and intelligent recognition. **a** Schematic of a nine-channel organic field-effect transistor (OFET) sensor array integrated with reservoir computing and k-nearest neighbors (KNN). **b** Time-dependent response profiles of all sensors to 26 gases. **c** Classification accuracy versus sensor number. **d** Protocol and array response to 12 typical gas mixtures. **e** Confusion matrix for 26-class gas recognition. **f** KNN decision boundaries among isomer/homolog gas pairs in feature space. **g** Mobile robot equipped with the olfactory array for real-world groundwater pollutant detection. **h** Confusion matrix of pollutant recognition on 24 samples. **a-h** Reproduced with permission [164]. Copyright 2025, Wiley–VCH GmbH. **i** Schematic of a visual–tactile mechano-optical synaptic array for in-sensor multimodal computing. **j** Array output under separate and combined light/mechanical stimulation. **k** Schematic of pattern recognition using an ANN with unimodal and multimodal datasets. **l** Training curves of ANN accuracy. **i-l** Reproduced with permission [165]. Copyright 2025, Wiley–VCH GmbH

Building on this trajectory, Wu et al. pioneered a biomimetic olfactory neuron array, architected through the synergistic integration of an organic field-effect transistor (OFET) sensor array, in-sensor RC, and K-nearest neighbors (KNN) classification [188]. The design, as illustrated in Fig. 13a, depicts the hardware–software co-design, detailing the flow from OFET sensor signal acquisition to temporal encoding and RC-driven feature extraction, culminating in KNN-based gas classification. The experimental methodology for high-resolution gas fingerprinting is clarified in Fig. 13b, which presents the 32-point temporal response sampling protocol for each analyte, forming the foundational feature set for all subsequent classification.

The impact of sensor diversity on classification performance is quantitatively demonstrated in Fig. 13c, where accuracy is plotted as a function of sensor array size: while single-sensor setups struggle (78.9%–98.1%), arrays comprising up to nine sensors achieve near-perfect discrimination (99.04%). The nuanced response behaviors, especially for gas mixtures, are further visualized in Fig. 13d, where nonadditive, nonlinear mixture signals underscore the necessity of multidimensional encoding and advanced feature extraction. Central to real-world application, the confusion matrix in Fig. 13e compellingly visualizes classification outcomes for the full 26-gas library, its dominant diagonal evidencing minimal misclassification and maximal specificity. The multidimensionality of the feature space and the robustness of boundary delineation are captured in Fig. 13f, which shows KNN-derived decision boundaries for 26 analytes on a pentacene-based sensor, with isomers and homologs clearly resolved as distinct clusters.

Transitioning to practical deployment, Fig. 13g schematizes the integration of the olfactory sensor array onto a robot dog platform for groundwater pollutant analysis, highlighting the augmentation of environmental intelligence through olfactory-visual sensor fusion. Experimental results, shown in Fig. 13h, provide a detailed breakdown of classification accuracy for individual pollutants and their mixtures, nitrobenzene (NB), dimethyl methylphosphonate (DMMP), isopropylamine (IPAm), and their binary combinations, where the KNN-based elimination voting model (KNN-EV) achieves 100% accuracy, again confirmed by the diagonal strength of the confusion matrix. This real-world demonstration validates the efficacy of in-sensor computation and memory in autonomous, resource-constrained contexts.

Expanding the scope to multimodal fusion at the device array level, Guo et al. engineer a 28 × 28-pixel mechano-optical synapse, whose fabrication and architecture are depicted in Fig. 3i [189]. The device enables simultaneous encoding of patterned light (visual) and mechanical (tactile) stimuli, mapped into a photo-stimulated luminescence (PSL)-based memory layer via mechanoluminescent (ML)-mediated luminescence transfer. The dynamical response to 30 consecutive pulses, mechanical, optical, and hybrid is chronologically charted in Fig. 13j, with early pulse data elucidating the pronounced enhancement resulting from synchronized force-light excitation. Integration with an ANN is visualized in Fig. 13k, where datasets are partitioned by sensory modality and the network structure (784 input neurons, 50 hidden, 5 output) is depicted, demonstrating the translation from array-level physical signals to computationally accessible features. The ultimate system-level performance is quantified in Fig. 13l, which tracks recognition accuracy across training epochs for tactile, visual, and visual–tactile modes, revealing a significant multimodal synergy: joint perception achieves a superior 92.5% accuracy, compared to 69.6% and 79.6% for unimodal inputs, respectively.

### Heterogeneous Integration Arrays

The ongoing quest for edge-intelligent systems that seamlessly bridge perception, computation, and action has catalyzed the emergence of heterogeneous integration arrays, amalgamating diverse device functionalities to enable closed-loop, adaptive decision-making in dynamic environments. Traditional von Neumann architectures, marked by a physical separation between memory and logic units, have long been hindered by the inefficiency of data shuttling, an impediment exacerbated by the massive parallelism and high precision required in advanced AI tasks such as dynamic object tracking (DOT), tactile cognition, and real-time multimodal recognition [190–192]. In heterogeneous integration arrays, sensors, synapses, and neurons are co-designed to the target task, with dynamics chosen to match requirements and to enable effective coupling of sensing, memory, and computation. In response, computing-in-memory (CIM) paradigms based on emerging nonvolatile memories, memristors, phase-change memories, and particularly, ferroelectric field-effect transistors (FeFETs), have transformed the landscape by supporting in situ vector–matrix multiplication (VMM) and robust Boolean logic, yet single-modality arrays often fall short in meeting the multidimensional demands of autonomous agents and robotic platforms [43, 193].

Recent efforts have begun to demonstrate how heterogeneous device integration can embed not just memory and logic, but also perception–action coupling within a single hardware fabric. For example, Shan et al. constructed a hemispherical optoelectronic memristor array using Ag-titanium dioxide (TiO_2_) nanoclusters, emulating binocular stereo vision and enabling in-pixel depth perception and motion detection via optical modulation and plasmonic effects [159]. Here, per-pixel optical gain and synaptic state jointly act as reconfigurable “meta-weights,” allowing depth cues and motion salience to be encoded before digitization, which reduces downstream calibration and preserves precision at scale. More recently, Wang et al. reported a vertically integrated tantalum-oxide (TaO_x_) memristor with IGZO photodetector layers to create a spiking cone photoreceptor array (VISCP), achieving ultra-low power (≤ 400 pW), color-selective spiking responses, and hardware-level depth perception via spiking frequency differentials [194]. The spiking threshold and adaptation within the VISCP provide a tunable temporal gate, aligning sensor dynamics with sequence learning and thereby serving as an explicit temporal plasticity at the array level.

**Fig. 14:**
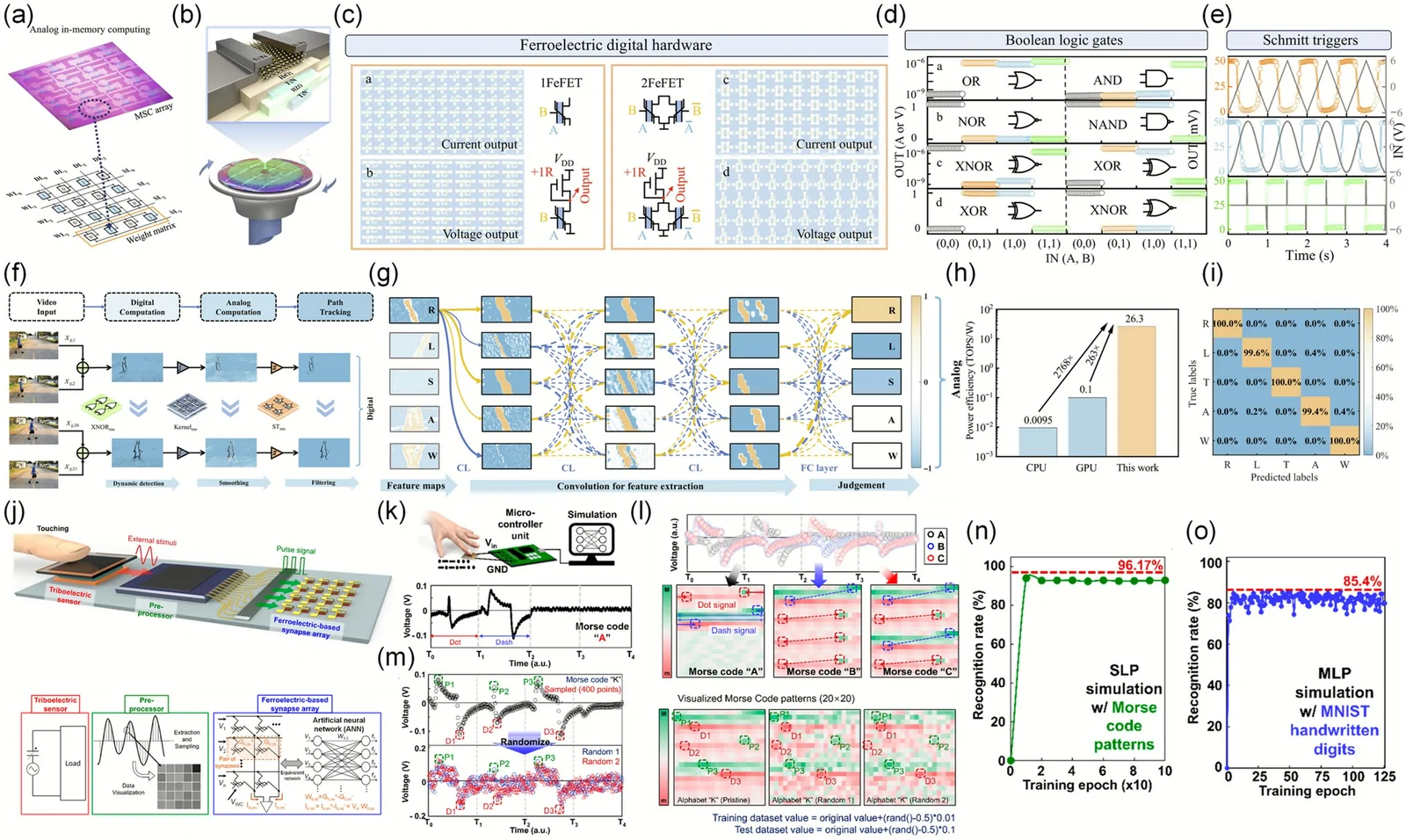
Heterogeneous integration arrays for neuromorphic sensing, computation, and action. **a** Optical image of 4 × 4 ferroelectric multistage cell (MSC) array and weight matrix. **b** Wafer-scale fabrication schematic for 2D MoS_2_-HfO_x_ ferroelectric field-effect transistors (FeFETs). **c** Optical images and circuit diagrams of FeFET-based Boolean logic and Schmitt trigger (ST) arrays. **d** Output signals of FeFET logic gates and STs. **e** Waveform transformation by Schmitt trigger: analog to digital logic. **f** Digital computation with ferroelectric XNOR, convolution, and ST arrays for real-time object detection. **g** Analog CNN computation in MSC arrays for multilayer feature extraction and prediction. **h** Power consumption comparison: ferroelectric hybrid system versus conventional CPUs/GPUs. **i** Confusion matrix showing 99.8% dynamic object tracking (DOT) recognition accuracy. **a-i** Reproduced with permission [48]. Copyright 2024, The American Association for the Advancement of Science. **j** Schematic of tactile neuromorphic system integrating triboelectric sensor, microcontroller, and FeFET array. **k** Tactile Morse code recognition: signal generation, image encoding, and neural network classification. **l** Real-time voltage output for Morse code “A”, “B”, “C” and visual patterns. **m** Training/test dataset generation by randomization. **n** single-layer perceptron (SLP) recognition accuracy on Morse code versus epoch. **o** MLP recognition accuracy on MNIST versus epochs. **j-o** Reproduced with permission [49]. Copyright 2023, American Chemical Society

In particularly, Lu et al. engineered a wafer-scale, 2D MoS_2_-HfO_x_ FeFET hybrid CIM system that monolithically integrates digital logic and analog multistage cell (MSC) arrays for analog VMM, achieving sub-femtojoule energy efficiency and a wafer-scale yield exceeding 96% [195]. The core hardware foundation is visually presented in Fig. 14a, which shows the fabricated 4 × 4 MSC array and its corresponding programmable weight matrix after precise row-by-row initialization, each cell supporting over 6-bit resolution and exceptional symmetry/linearity in conductance states. The platform’s device architecture and fabrication flow, including the solution-processable, wafer-scale growth of MoS_2_ channels and high-k hafnium oxide interfaces, are outlined in Fig. 14b, highlighting the system’s CMOS compatibility and scalability. On the digital front, Fig. 14c depicts optical images and the actual circuit implementation of FeFET-based Boolean logic arrays and Schmitt triggers (STs), forming the digital building blocks for in-memory computation. The practical operation of these digital circuits is detailed in Fig. 14d, which presents the output characteristics for key logic gates (AND, OR, XOR, full adder), as well as dynamic waveform transformations enabled by the Schmitt trigger. Crucially, array-uniform FeFET weight states provide consistent LTP/LTD kernels for direct weight mapping, while the Schmitt trigger supplies a programmable threshold with hysteresis that functions as module-level metaplasticity, shaping spike timing and noise immunity for temporal tasks.

Transitioning to system-level functionality, Fig. 14e provides an explicit demonstration of the Schmitt trigger’s role in pulse shaping, showing the transformation of input triangular and sine waves, and the resulting rail-to-rail logic state transitions with ultrafast response and robust noise immunity. Building on this hybrid platform, the digital computing pipeline for dynamic object tracking is detailed in Fig. 14f, where ferroelectric XNOR logic, convolutional filtering, and Schmitt trigger arrays collaboratively enable real-time moving object detection and dynamic background suppression within the array. In parallel, Fig. 14g visualizes the analog computation conducted in the MSC-based CNN array, showcasing the multilayered feature extraction and trajectory prediction flow powered entirely by analog in-memory operations. The platform’s energy and accuracy advantages are compellingly summarized in Fig. 14h, which benchmarks the hybrid system against traditional CPU (Intel i9) and GPU (NVIDIA Tesla V100) architectures. The results reveal more than two orders of magnitude improvement in energy efficiency, while maintaining high accuracy. The confusion matrix in Fig. 14i further demonstrates the system’s average DOT recognition accuracy of 99.8%, affirming the feasibility of hardware-level, real-time visual intelligence. Taken together, the hybrid array demonstrates plasticity engineering by delivering precise, linear weight updates for high fidelity mapping and by providing tunable thresholding with persistence control for time-aligned learning.

**Fig. 15:**
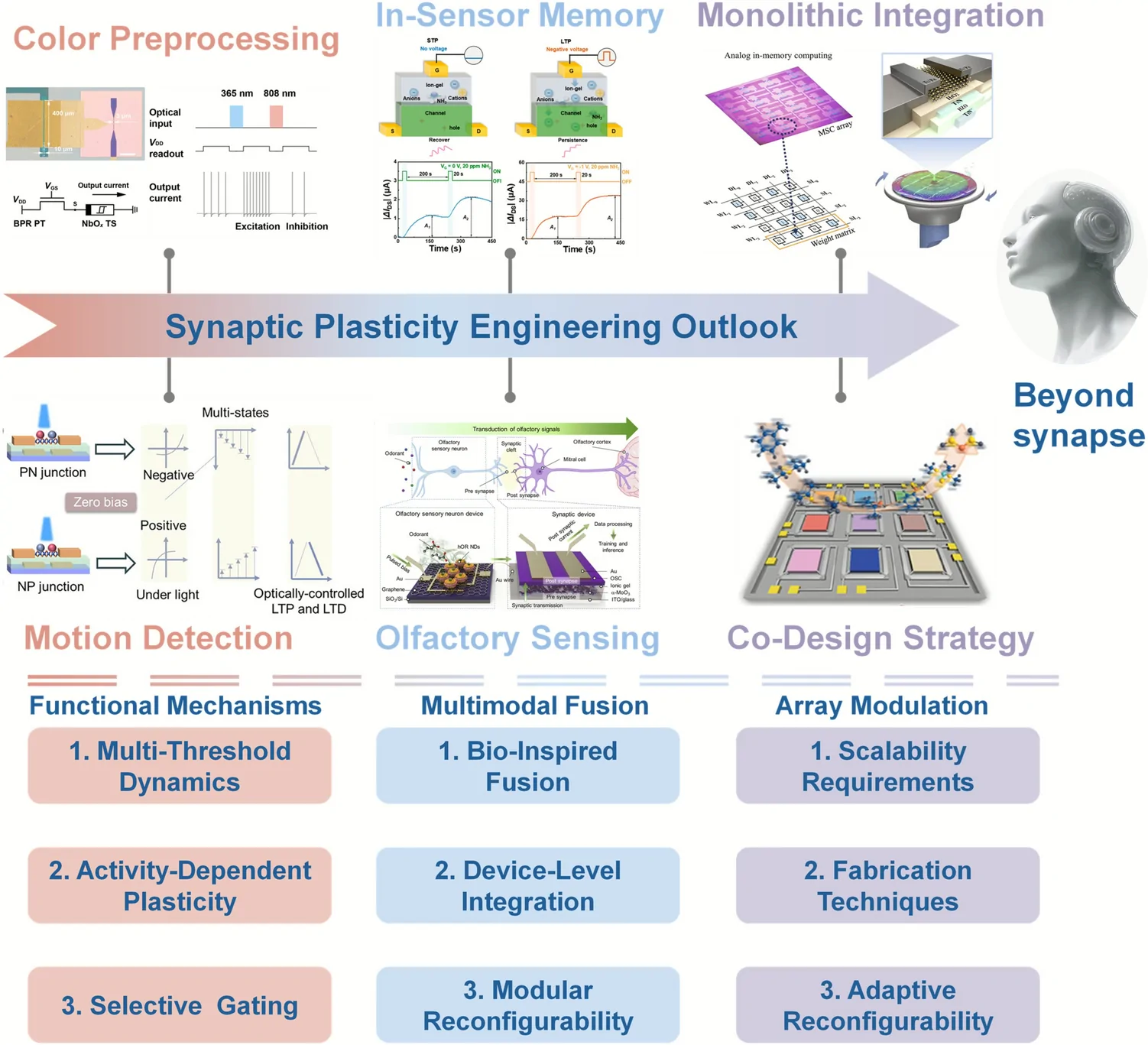
Outlook for future directions in synaptic plasticity engineering, covering functional mechanisms, multimodal fusion, and array modulation. Reproduced with permission [43]. Copyright 2025, The American Association for the Advancement of Science. Reproduced with permission [44]. Copyright 2024, The American Association for the Advancement of Science. Reproduced with permission [48]. Copyright 2024, The American Association for the Advancement of Science. Reproduced with permission [105]. Copyright 2024, American Chemical Society. Multimodal Fusion. Reproduced with permission [126]. Copyright 2024, American Chemical Society. Reproduced with permission [164]. Copyright 2025, Wiley–VCH GmbH Copyright 2025, Wiley– VCH GmbH

To extend this paradigm to tactile cognition, Kim et al. realized a bioinspired tactile neuromorphic system by integrating a triboelectric Cu/ poly(dimethylsiloxane) (PDMS) sensor and a MoS_2_/P(VDF-TrFE) FeFET synaptic array [196]. Figure 14j provides a schematic of the full tactile neuromorphic system, depicting the interaction between the triboelectric sensor, preprocessing microcontroller, and ferroelectric synaptic hardware, which together transduce tactile stimuli into structured electrical signals and process them in real time. For Morse code pattern recognition, Fig. 14k presents the system’s process and representative data, illustrating the conversion of tactile Morse code signals (“A”, “B”, “C”) into time-sequenced voltage patterns and their subsequent encoding as 20 × 20 visual maps for neural network input. Data augmentation, a key strategy for overcoming limited sample sets, is depicted in Fig. 14l, where randomized visualized Morse code images for the letter “K” exemplify robust data expansion and highlight how even significant noise does not obscure key pattern features. System-level recognition performance is quantitatively charted in Fig. 14m, which traces the evolution of single-layer perceptron (SLP) accuracy for Morse code learning and MLP accuracy for MNIST digit recognition, substantiating the system’s robustness and hardware efficiency. Figure 14n further summarizes the final recognition accuracy and confusion matrix, demonstrating high classification fidelity across all classes. Finally, Fig. 14o provides an additional benchmark by comparing the recognition accuracy of FeFET-based synaptic arrays with other synaptic device platforms, confirming their competitive advantage in both training convergence and generalization. In this tactile module, the synaptic array functions as a reconfigurable plastic substrate in which device-level domain polarization establishes stable, low-variance weights for precise mapping, and input rate-dependent integration, together with threshold adaptation, tunes the temporal window, thereby aligning plasticity with the statistics of tactile stimuli (Fig. 15).

## Conclusion and Outlook

This review systematically classifies and discusses artificial synaptic devices with a focus on the functional differentiation of synaptic plasticity behaviors. By examining diverse synaptic behaviors such as high-resolution and multilevel LTP and LTD, STM and FM processes, excitatory and inhibitory synergy, wavelength selectivity, dynamic threshold modulation, and multimodal sensory adaptation, the review clarifies how each type of plasticity enables key computational features in neuromorphic networks. This functional perspective highlights the essential connection between tailored synaptic behaviors and advanced neural system performance. Through comparative analysis and representative examples, it becomes clear that the deliberate modulation of plasticity behaviors, whether for analog weight stabilization, tunable memory retention, or cross-modal fusion, forms the basis for constructing efficient, flexible, and adaptive brain-inspired computing systems. However, current modulation methods still exhibit limitations regarding flexibility, diversity, and overall adaptability, and much of the related research remains exploratory. Therefore, based on this systematic analysis, the following three aspects are identified as critical future development directions in synaptic plasticity modulation techniques.

### Exploration of Novel Synaptic Plasticity Mechanisms

The exceptional intelligence of biological organisms primarily arises from the diverse and sophisticated plasticity mechanisms that operate within their nervous systems [61]. Compared with natural neural structures, artificial neuromorphic devices currently possess relatively simplistic and limited modulation mechanisms [2]. Specifically, existing synaptic plasticity research largely centers on basic transitions between STM and LTM states, modulation between excitatory and inhibitory synaptic activities, and optimization of linearity and symmetry in synaptic weight updating [15]. These relatively narrow modulation pathways significantly restrict the full potential and functionality of neuromorphic systems [197]. The limited scope of plasticity modulation is mainly attributed to the current absence of sufficiently rich and diverse physical mechanisms at the device level. Thus, future research should prioritize exploring and discovering new functional mechanisms, such as multithreshold dynamics, activity-dependent metaplasticity, selective plasticity gating, and biologically inspired heterosynaptic mechanisms, to expand the modulation versatility [198, 199]. Enriching the pool of available plasticity behaviors can effectively enhance the operational speed, power efficiency, computational flexibility, and adaptive learning capability of neuromorphic chips.

### Multimodal Collaborative Plasticity Modulation Techniques

Future advancements in neuromorphic hardware require substantial progress in multimodal collaborative plasticity modulation, shifting away from single-sensory, isolated processing paradigms toward the seamless integration of diverse physical signals and functionalities. Inspired by the multisensory fusion and parallel processing capabilities of biological neural systems, novel device designs must effectively merge multiple sensory inputs, adaptive memory states, and computational functions at a device and module level [142]. Achieving this vision involves exploring advanced heterostructures, integrating sensing, logic, and memory into unified physical nodes, and developing reconfigurable modular units that dynamically emulate closed-loop biological perception–action pathways [152]. Such multimodal integration techniques will significantly enhance the adaptability, robustness, and resource efficiency of neuromorphic systems, laying critical groundwork for intelligent sensing and decision-making in real-world, resource-constrained environments.

### Enhanced Plasticity Modulation Techniques for Large-Scale Neuromorphic Arrays

The practical deployment of neuromorphic computing relies critically on synaptic plasticity modulation methods tailored explicitly for scalable neural arrays. Although significant advancements in individual synaptic devices have been achieved, large-scale integration demands higher standards for uniformity, reproducibility, and stability of plasticity behaviors. To meet these requirements, future research should prioritize the development of advanced fabrication technologies and array-level modulation techniques that can reliably and uniformly regulate synaptic plasticity across extensive device arrays [200]. Furthermore, exploring gate-tunable and dynamically reconfigurable plasticity mechanisms, exemplified by multifunctional organic heterostructure arrays, will allow adaptive synaptic modulation essential for flexible, real-time neural computations [201]. Achieving these goals will significantly enhance the robustness, efficiency, and adaptability of large-scale neuromorphic systems, laying the groundwork for their broader application in intelligent edge computing.

Despite notable progress, current modulation strategies remain constrained in flexibility, diversity, and large-scale coordination. In particularly, application-facing advances should explicitly link device-level plasticity to task requirements-stable analog weights for high-accuracy learning, tunable short-term dynamics for temporal processing, and hardware attention for noise-resilient perception. Future research should focus on enriching the behavioral repertoire of plasticity, especially those supporting temporal learning, adaptive attention, and metaplastic threshold tuning, advancing cross-modal convergence and device-algorithm co-design to bridge device behaviors with network objectives, and improving array-level uniformity and variation-aware calibration for scalable integration and reliable operation in edge and resource-constrained environments. In parallel, standardized, task-linked benchmarks and device-to-network mapping protocols (linearity, symmetry, drift/temperature stability, STM windows, STDP, and threshold adaptability) are needed to ensure fair comparison and reproducible system-level gains. Together, these directions will pave the way toward deployable, high-efficiency neuromorphic intelligence. Priority application sandboxes—wearable health monitoring, low-light/night-vision perception, and extreme-environment sensing—can serve as near-term proving grounds for translating synaptic strategies into deployable systems.

## References

[1] K. Lu, X. Li, Q. Sun, X. Pang, J. Chen et al., Solution-processed electronics for artificial synapses. Mater. Horiz. **8**(2), 447–470 (2021). 10.1039/d0mh01520b 34821264 10.1039/d0mh01520b

[2] J.J. Yang, D.B. Strukov, D.R. Stewart, Memristive devices for computing. Nat. Nanotechnol. **8**(1), 13–24 (2013). 10.1038/nnano.2012.240 23269430 10.1038/nnano.2012.240

[3] L. Sun, W. Wang, H. Yang, Recent progress in synaptic devices based on 2D materials. Adv. Intell. Syst. **2**(5), 1900167 (2020). 10.1002/aisy.201900167

[4] G. Cao, P. Meng, J. Chen, H. Liu, R. Bian et al., 2D material based synaptic devices for neuromorphic computing. Adv. Funct. Mater. **31**(4), 2005443 (2021). 10.1002/adfm.202005443

[5] S. Meng, J. Jiang, Q. Ju, Y. Wang, D. Wu et al., Adaptive sub-nanometer control of a piezoelectric positioning platform. IEEE Trans. Autom. Sci. Eng. **22**, 22755–22765 (2025). 10.1109/TASE.2025.3615572

[6] Z. Wang, H. Wu, G.W. Burr, C.S. Hwang, K.L. Wang et al., Resistive switching materials for information processing. Nat. Rev. Mater. **5**(3), 173–195 (2020). 10.1038/s41578-019-0159-3

[7] X. Zou, S. Xu, X. Chen, L. Yan, Y. Han, Breaking the von Neumann bottleneck: architecture-level processing-in-memory technology. Sci. China Inf. Sci. **64**(6), 160404 (2021). 10.1007/s11432-020-3227-1

[8] R. Pendurthi, D. Jayachandran, A. Kozhakhmetov, N. Trainor, J.A. Robinson et al., Heterogeneous integration of atomically thin semiconductors for non-von Neumann CMOS. Small **18**(33), e2202590 (2022). 10.1002/smll.202202590 35843869 10.1002/smll.202202590

[9] L.F. Abbott, S.B. Nelson, Synaptic plasticity: taming the beast. Nat. Neurosci. **3**(S11), 1178–1183 (2000). 10.1038/81453 11127835 10.1038/81453

[10] L. Lu, B. Sun, Z. Wang, J. Meng, T. Wang, Two-dimensional MXene-based advanced sensors for neuromorphic computing intelligent application. Nano-Micro Lett. **18**(1), 64 (2025). 10.1007/s40820-025-01902-1 10.1007/s40820-025-01902-1 PMC12432000 40938496

[11] Z. Zhu, J. Shui, T. Wang, J. Meng, Mechanical properties analysis of flexible memristors for neuromorphic computing. Nano-Micro Lett. **18**(1), 2 (2025). 10.1007/s40820-025-01825-x 10.1007/s40820-025-01825-x PMC12271050 40676361

[12] I. Boybat, M. Le Gallo, S.R. Nandakumar, T. Moraitis, T. Parnell et al., Neuromorphic computing with multi-memristive synapses. Nat. Commun. **9**(1), 2514 (2018). 10.1038/s41467-018-04933-y 29955057 10.1038/s41467-018-04933-y PMC6023896

[13] Z. Liu, Y. Fang, Z. Cai, Y. Liu, X. Zhao et al., Constructing a complex hybrid neural network for biomimetic spatial and temporal perception. Small **21**(35), e2506100 (2025). 10.1002/smll.202506100 40619773 10.1002/smll.202506100

[14] F. Chen, Y. Zhou, Y. Zhu, R. Zhu, P. Guan et al., Recent progress in artificial synaptic devices: materials, processing and applications. J. Mater. Chem. C **9**(27), 8372–8394 (2021). 10.1039/d1tc01211h

[15] Z. Wang, S. Joshi, S.E. Savel’ev, H. Jiang, R. Midya et al., Memristors with diffusive dynamics as synaptic emulators for neuromorphic computing. Nat. Mater. **16**(1), 101–108 (2017). 10.1038/nmat4756 27669052 10.1038/nmat4756

[16] R. Midya, Z. Wang, J. Zhang, S.E. Savel’ev, C. Li et al., Anatomy of Ag/*Hafnia*-based selectors with 10(10) nonlinearity. Adv. Mater. **29**(12), 1604457 (2017). 10.1002/adma.201604457 10.1002/adma.201604457 28134458

[17] S. Yan, J. Zang, P. Xu, Y. Zhu, G. Li et al., Recent progress in ferroelectric synapses and their applications. Sci. China Mater. **66**(3), 877–894 (2023). 10.1007/s40843-022-2318-9

[18] J. Zeng, G. Feng, G. Wu, J. Liu, Q. Zhao et al., Multisensory ferroelectric semiconductor synapse for neuromorphic computing. Adv. Funct. Mater. **34**(19), 2313010 (2024). 10.1002/adfm.202313010

[19] R.D. Nikam, M. Kwak, H. Hwang, All-solid-state oxygen ion electrochemical random-access memory for neuromorphic computing. Adv. Electron. Mater. **7**(5), 2100142 (2021). 10.1002/aelm.202100142

[20] J.-M. Yu, C. Lee, D.-J. Kim, H. Park, J.-K. Han et al., All-solid-state ion synaptic transistor for wafer-scale integration with electrolyte of a nanoscale thickness. Adv. Funct. Mater. **31**(23), 2010971 (2021). 10.1002/adfm.202010971

[21] W. Maass, T. Natschläger, H. Markram, Real-time computing without stable states: a new framework for neural computation based on perturbations. Neural Comput. **14**(11), 2531–2560 (2002). 10.1162/089976602760407955 12433288 10.1162/089976602760407955

[22] R.S. Zucker, W.G. Regehr, Short-term synaptic plasticity. Annu. Rev. Physiol. **64**, 355–405 (2002). 10.1146/annurev.physiol.64.092501.114547 11826273 10.1146/annurev.physiol.64.092501.114547

[23] M.-K. Kim, J.-S. Lee, Short-term plasticity and long-term potentiation in artificial biosynapses with diffusive dynamics. ACS Nano **12**(2), 1680–1687 (2018). 10.1021/acsnano.7b08331 29357225 10.1021/acsnano.7b08331

[24] Y. He, Z. Ge, Z. Li, Z. Li, R. Liu et al., All-polymer organic electrochemical synaptic transistor with controlled ionic dynamics for high-performance wearable and sustainable reservoir computing. Adv. Funct. Mater. **35**(8), 2415595 (2025). 10.1002/adfm.202415595

[25] P. Guo, J. Zhang, J. Huang, Recent progress in organic optoelectronic synaptic transistor arrays: fabrication strategies and innovative applications of system integration. J. Semicond. **46**(2), 021405 (2025). 10.1088/1674-4926/24120017

[26] Y. Xu, W. Liu, Y. Huang, C. Jin, B. Zhou et al., Recent advances in flexible organic synaptic transistors. Adv. Electron. Mater. **7**(11), 2100336 (2021). 10.1002/aelm.202100336

[27] S. Saïghi, C.G. Mayr, T. Serrano-Gotarredona, H. Schmidt, G. Lecerf et al., Plasticity in memristive devices for spiking neural networks. Front. Neurosci. **9**, 51 (2015). 10.3389/fnins.2015.00051 25784849 10.3389/fnins.2015.00051 PMC4345885

[28] S. Wang, H. Chen, T. Liu, Y. Wei, G. Yao et al., Retina-inspired organic photonic synapses for selective detection of SWIR light. Angew. Chem. Int. Ed. **62**(6), e202213733 (2023). 10.1002/anie.202213733 10.1002/anie.202213733 36418239

[29] J. Zhang, P. Guo, Z. Guo, L. Li, T. Sun et al., Retina-inspired artificial synapses with ultraviolet to near-infrared broadband responses for energy-efficient neuromorphic visual systems. Adv. Funct. Mater. **33**(32), 2302885 (2023). 10.1002/adfm.202302885

[30] Z. Guo, J. Zhang, J. Wang, X. Liu, P. Guo et al., Organic synaptic transistors with environmentally friendly core/shell quantum dots for wavelength-selective memory and neuromorphic functions. Nano Lett. **24**(20), 6139–6147 (2024). 10.1021/acs.nanolett.4c01606 38722705 10.1021/acs.nanolett.4c01606

[31] D. Kim, J.-S. Lee, Neurotransmitter-induced excitatory and inhibitory functions in artificial synapses. Adv. Funct. Mater. **32**(21), 2200497 (2022). 10.1002/adfm.202200497

[32] Y.-C. Mi, C.-H. Yang, L.-C. Shih, J.-S. Chen, All-optical-controlled excitatory and inhibitory synaptic signaling through bipolar photoresponse of an oxide-based phototransistor. Adv. Opt. Mater. **11**(14), 2300089 (2023). 10.1002/adom.202300089

[33] Z. Wang, M. Li, H. Yang, S. Shao, J. Li et al., Enhancement-mode carbon nanotube optoelectronic synaptic transistors with large and controllable threshold voltage modulation window for broadband flexible vision systems. ACS Nano **18**(22), 14298–14311 (2024). 10.1021/acsnano.4c00166 38787538 10.1021/acsnano.4c00166

[34] J.-K. Han, M.-W. Lee, J.-M. Yu, Y.-K. Choi, A single transistor-based threshold switch for a bio-inspired reconfigurable threshold logic. Adv. Electron. Mater. **7**(5), 2100117 (2021). 10.1002/aelm.202100117

[35] J. Jiang, W. Xu, Z. Sun, L. Fu, S. Zhang et al., Wavelength-controlled photoconductance polarity switching *via* harnessing defects in doped PdSe_2_ for artificial synaptic features. Small **20**(13), 2306068 (2024). 10.1002/smll.202306068 10.1002/smll.202306068 37963834

[36] Z. Wang, L. Lu, J. Meng, T. Wang, Emerging negative photoconductivity effect-based synaptic device for optoelectronic in-sensor computing. Adv. Mater. **37**(32), e2504710 (2025). 10.1002/adma.202504710 40417972 10.1002/adma.202504710

[37] W.-A. Mo, G. Ding, Z. Nie, Z. Feng, K. Zhou et al., Spatiotemporal modulation of plasticity in multi-terminal tactile synaptic transistor. Adv. Electron. Mater. **9**(1), 2200733 (2023). 10.1002/aelm.202200733

[38] X. Liu, S. Wang, Z. Di, H. Wu, C. Liu et al., An optoelectronic synapse based on two-dimensional violet phosphorus heterostructure. Adv. Sci. **10**(22), 2301851 (2023). 10.1002/advs.202301851 10.1002/advs.202301851 PMC10401094 37229772

[39] P. Langner, F. Chiabrera, N. Alayo, P. Nizet, L. Morrone et al., Solid-state oxide-ion synaptic transistor for neuromorphic computing. Adv. Mater. **37**(7), e2415743 (2025). 10.1002/adma.202415743 39722152 10.1002/adma.202415743

[40] Q. Lin, Y. Zhu, J. Sun, S. Peng, Z. Wang et al., A full-quantum-dot optoelectronic memristor for in-sensor reservoir computing system with integrated functions. Adv. Funct. Mater. **35**(30), 2423548 (2025). 10.1002/adfm.202423548

[41] Y.-B. Leng, Z. Lv, S. Huang, P. Xie, H.-X. Li et al., A near-infrared retinomorphic device with high dimensionality reservoir expression. Adv. Mater. **36**(48), 2411225 (2024). 10.1002/adma.202411225 39390822 10.1002/adma.202411225 PMC11602693

[42] H. Choi, S. Baek, H. Jung, T. Kang, S. Lee et al., Spiking neural network integrated with impact ionization field-effect transistor neuron and a ferroelectric field-effect transistor synapse. Adv. Mater. **37**(26), 2406970 (2025). 10.1002/adma.202406970 10.1002/adma.202406970 39233555

[43] M. Yan, Q. Zhu, S. Wang, Y. Ren, G. Feng et al., Ferroelectric synaptic transistor network for associative memory. Adv. Electron. Mater. **7**(4), 2001276 (2021). 10.1002/aelm.202001276

[44] M. Huang, X. Liu, F. Yu, J. Li, J. Huang et al., Plasmon-enhanced optoelectronic graded neurons for dual-waveband image fusion and motion perception. Adv. Mater. **37**(4), 2412993 (2025). 10.1002/adma.202412993 10.1002/adma.202412993 39648673

[45] J. Jiang, X. Shan, J. Xu, Y. Sun, T.-F. Xiang et al., Retina-like chlorophyll heterojunction-based optoelectronic memristor with all-optically modulated synaptic plasticity enabling neuromorphic edge detection. Adv. Funct. Mater. **34**(51), 2409677 (2024). 10.1002/adfm.202409677

[46] D. Li, G. Liu, F. Li, H. Ren, Y. Tang et al., Double-opponent spiking neuron array with orientation selectivity for encoding and spatial-chromatic processing. Sci. Adv. **11**(7), eadt3584 (2025). 10.1126/sciadv.adt3584 39937908 10.1126/sciadv.adt3584 PMC11817925

[47] S. Woo, D. Moon, Y. Won, C. Kyung, J. Yoo et al., A pattern recognition artificial olfactory system based on human olfactory receptors and organic synaptic devices. Sci. Adv. **10**(21), eadl2882 (2024). 10.1126/sciadv.adl2882 38781346 10.1126/sciadv.adl2882 PMC11114221

[48] J. Hu, H. Li, Y. Zhang, J. Zhou, Y. Zhao et al., Reconfigurable neuromorphic computing with 2D material heterostructures for versatile neural information processing. Nano Lett. **24**(30), 9391–9398 (2024). 10.1021/acs.nanolett.4c02658 39038296 10.1021/acs.nanolett.4c02658

[49] Y. Chen, H. Wang, H. Chen, W. Zhang, M. Pätzel et al., Li promoting long afterglow organic light-emitting transistor for memory optocoupler module. Adv. Mater. **36**(27), 2402515 (2024). 10.1002/adma.202402515 10.1002/adma.202402515 38616719

[50] R. Li, Z. Yue, H. Luan, Y. Dong, X. Chen et al., Multimodal artificial synapses for neuromorphic application. Research **7**, 427 (2024). 10.34133/research.0427 10.34133/research.0427 PMC11331013 39161534

[51] D. Zendrikov, S. Solinas, G. Indiveri, Brain-inspired methods for achieving robust computation in heterogeneous mixed-signal neuromorphic processing systems. Neuromorph. Comput. Eng. **3**(3), 034002 (2023). 10.1088/2634-4386/ace64c

[52] M.C. Sahu, S. Sahoo, S.K. Mallik, A.K. Jena, S. Sahoo, Multifunctional 2D MoS_2_ optoelectronic artificial synapse with integrated arithmetic and reconfigurable logic operations for in-memory neuromorphic computing applications. Adv. Mater. Technol. **8**(2), 2201125 (2023). 10.1002/admt.202201125

[53] H. Wan, J. Zhao, L.-W. Lo, Y. Cao, N. Sepúlveda et al., Multimodal artificial neurological sensory–memory system based on flexible carbon nanotube synaptic transistor. ACS Nano **15**(9), 14587–14597 (2021). 10.1021/acsnano.1c04298 34472329 10.1021/acsnano.1c04298

[54] C. Weilenmann, A.N. Ziogas, T. Zellweger, K. Portner, M. Mladenović et al., Single neuromorphic memristor closely emulates multiple synaptic mechanisms for energy efficient neural networks. Nat. Commun. **15**(1), 6898 (2024). 10.1038/s41467-024-51093-3 39138160 10.1038/s41467-024-51093-3 PMC11322324

[55] C. Choi, H. Kim, J.-H. Kang, M.-K. Song, H. Yeon et al., Reconfigurable heterogeneous integration using stackable chips with embedded artificial intelligence. Nat. Electron. **5**(6), 386–393 (2022). 10.1038/s41928-022-00778-y

[56] S.H. Jo, T. Chang, I. Ebong, B.B. Bhadviya, P. Mazumder et al., Nanoscale memristor device as synapse in neuromorphic systems. Nano Lett. **10**(4), 1297–1301 (2010). 10.1021/nl904092h 20192230 10.1021/nl904092h

[57] R.A. John, J. Ko, M.R. Kulkarni, N. Tiwari, N.A. Chien et al., Flexible ionic-electronic hybrid oxide synaptic TFTs with programmable dynamic plasticity for brain-inspired neuromorphic computing. Small **13**(32), 1701193 (2017). 10.1002/smll.201701193 10.1002/smll.201701193 28656608

[58] M.-K. Song, J.-H. Kang, X. Zhang, W. Ji, A. Ascoli et al., Recent advances and future prospects for memristive materials, devices, and systems. ACS Nano **17**(13), 11994–12039 (2023). 10.1021/acsnano.3c03505 37382380 10.1021/acsnano.3c03505

[59] S.-M. Kim, S. Kim, L. Ling, S.E. Liu, S. Jin et al., Linear and symmetric Li-based composite memristors for efficient supervised learning. ACS Appl. Mater. Interfaces **14**(4), 5673–5681 (2022). 10.1021/acsami.1c24562 35043617 10.1021/acsami.1c24562

[60] S. Dai, Y. Zhao, Y. Wang, J. Zhang, L. Fang et al., Recent advances in transistor-based artificial synapses. Adv. Funct. Mater. **29**(42), 1903700 (2019). 10.1002/adfm.201903700

[61] A. Citri, R.C. Malenka, Synaptic plasticity: multiple forms, functions, and mechanisms. Neuropsychopharmacology **33**(1), 18–41 (2008). 10.1038/sj.npp.1301559 17728696 10.1038/sj.npp.1301559

[62] X. Niu, B. Tian, Q. Zhu, B. Dkhil, C. Duan, Ferroelectric polymers for neuromorphic computing. Appl. Phys. Rev. **9**(2), 021309 (2022). 10.1063/5.0073085

[63] B. Tian, L. Liu, M. Yan, J. Wang, Q. Zhao et al., A robust artificial synapse based on organic ferroelectric polymer. Adv. Electron. Mater. **5**(1), 1800600 (2019). 10.1002/aelm.201800600

[64] P. Guo, J. Zhang, Z. Hua, T. Sun, L. Li et al., Organic synaptic transistors based on a semiconductor heterojunction for artificial visual and neuromorphic functions. Nano Lett. **25**(8), 3204–3211 (2025). 10.1021/acs.nanolett.4c05809 39960419 10.1021/acs.nanolett.4c05809

[65] Z. Lv, M.-H. Jiang, H.-Y. Liu, Q.-X. Li, T. Xie et al., Temperature-resilient polymeric memristors for effective deblurring in static and dynamic imaging. Adv. Funct. Mater. **35**(23), 2424382 (2025). 10.1002/adfm.202424382

[66] Y. Chen, M. Zhang, D. Li, Y. Tang, H. Ren et al., Bidirectional synaptic phototransistor based on two-dimensional ferroelectric semiconductor for mixed color pattern recognition. ACS Nano **17**(13), 12499–12509 (2023). 10.1021/acsnano.3c02167 37345912 10.1021/acsnano.3c02167

[67] G.W. Baek, Y.J. Kim, J. Kim, J.H. Chang, U. Kim et al., Memristive switching mechanism in colloidal InP/ZnSe/ZnS quantum dot-based synaptic devices for neuromorphic computing. Nano Lett. **24**(19), 5855–5861 (2024). 10.1021/acs.nanolett.4c01083 38690800 10.1021/acs.nanolett.4c01083

[68] D.H. Choi, J.B. An, J. Chung, K. Park, H. Lee et al., Synergistic enhancement of long-term plasticity in solid-state electrolyte-gated synaptic transistors realized by introducing an ion-capturing layer. Nano Today **61**, 102631 (2025). 10.1016/j.nantod.2025.102631

[69] M.A. Zidan, J.P. Strachan, W.D. Lu, The future of electronics based on memristive systems. Nat. Electron. **1**(1), 22–29 (2018). 10.1038/s41928-017-0006-8

[70] L.F. Abbott, W.G. Regehr, Synaptic computation. Nature **431**(7010), 796–803 (2004). 10.1038/nature03010 15483601 10.1038/nature03010

[71] M. Prezioso, F. Merrikh-Bayat, B.D. Hoskins, G.C. Adam, K.K. Likharev et al., Training and operation of an integrated neuromorphic network based on metal-oxide memristors. Nature **521**(7550), 61–64 (2015). 10.1038/nature14441 25951284 10.1038/nature14441

[72] Y. Zhang, Q. Zhu, B. Tian, C. Duan, New-generation ferroelectric AlScN materials. Nano-Micro Lett **16**(1), 227 (2024). 10.1007/s40820-024-01441-1 10.1007/s40820-024-01441-1 PMC11199478 38918252

[73] J. Zhu, C. Liu, R. Gao, Y. Zhang, H. Zhang et al., Ultra-flexible high-linearity silicon nanomembrane synaptic transistor array. Adv. Mater. **37**(7), e2413404 (2025). 10.1002/adma.202413404 39748631 10.1002/adma.202413404

[74] Y. Hwang, B. Park, S. Hwang, S.-W. Choi, H.S. Kim et al., A bioinspired ultra flexible artificial van der Waals 2D-MoS_2_ channel/LiSiO_*x*_ solid electrolyte synapse arrays *via* laser-lift off process for wearable adaptive neuromorphic computing. Small Methods **7**(7), 2201719 (2023). 10.1002/smtd.202201719 10.1002/smtd.202201719 36960927

[75] G. Feng, Q. Zhu, X. Liu, L. Chen, X. Zhao et al., A ferroelectric fin diode for robust non-volatile memory. Nat. Commun. **15**(1), 513 (2024). 10.1038/s41467-024-44759-5 38218871 10.1038/s41467-024-44759-5 PMC10787831

[76] B. Tian, Z. Xie, L. Chen, S. Hao, Y. Liu et al., Ultralow-power in-memory computing based on ferroelectric memcapacitor network. Exploration **3**(3), 20220126 (2023). 10.1002/EXP.20220126 37933380 10.1002/EXP.20220126 PMC10624373

[77] G. Zhang, J. Qin, Y. Zhang, G. Gong, Z.-Y. Xiong et al., Functional materials for memristor-based reservoir computing: dynamics and applications. Adv. Funct. Mater. **33**(42), 2302929 (2023). 10.1002/adfm.202302929

[78] S.H. Sung, T.J. Kim, H. Shin, T.H. Im, K.J. Lee, Simultaneous emulation of synaptic and intrinsic plasticity using a memristive synapse. Nat. Commun. **13**(1), 2811 (2022). 10.1038/s41467-022-30432-2 35589710 10.1038/s41467-022-30432-2 PMC9120471

[79] J. Song, J. Meng, C. Lu, T. Wang, C. Wan et al., Self-powered optoelectronic synaptic device for both static and dynamic reservoir computing. Nano Energy **134**, 110574 (2025). 10.1016/j.nanoen.2024.110574

[80] H.-J. Kim, D.-S. Woo, S.-M. Jin, H.-J. Kwon, K.-H. Kwon et al., Super-linear-threshold-switching selector with multiple jar-shaped Cu-filaments in the amorphous Ge3Se7 resistive switching layer in a cross-point synaptic memristor array. Adv. Mater. **34**(40), 2203643 (2022). 10.1002/adma.202203643 10.1002/adma.202203643 35980937

[81] X. Li, Y. Zhong, H. Chen, J. Tang, X. Zheng et al., A memristors-based dendritic neuron for high-efficiency spatial-temporal information processing. Adv. Mater. **35**(37), e2203684 (2023). 10.1002/adma.202203684 35735048 10.1002/adma.202203684

[82] X. Wu, S. Shi, B. Liang, Y. Dong, R. Yang et al., Ultralow-power optoelectronic synaptic transistors based on polyzwitterion dielectrics for in-sensor reservoir computing. Sci. Adv. **10**(16), eadn4524 (2024). 10.1126/sciadv.adn4524 38630830 10.1126/sciadv.adn4524 PMC11023521

[83] J. Liu, G. Feng, W. Li, S. Hao, S. Han et al., Physical reservoir computing for Edge AI applications. Innov. Mater. **3**(2), 100127 (2025). 10.59717/j.xinn-mater.2025.100127

[84] A. Bednarkiewicz, M. Szalkowski, M. Majak, Z. Korczak, M. Misiak et al., All-optical data processing with photon-avalanching nanocrystalline photonic synapse. Adv. Mater. **35**(42), e2304390 (2023). 10.1002/adma.202304390 37572370 10.1002/adma.202304390

[85] J. Pei, L. Deng, S. Song, M. Zhao, Y. Zhang et al., Towards artificial general intelligence with hybrid Tianjic chip architecture. Nature **572**(7767), 106–111 (2019). 10.1038/s41586-019-1424-8 31367028 10.1038/s41586-019-1424-8

[86] R. Brette, W. Gerstner, Adaptive exponential integrate-and-fire model as an effective description of neuronal activity. J. Neurophysiol. **94**(5), 3637–3642 (2005). 10.1152/jn.00686.2005 16014787 10.1152/jn.00686.2005

[87] J. Feldmann, N. Youngblood, C.D. Wright, H. Bhaskaran, W.H.P. Pernice, All-optical spiking neurosynaptic networks with self-learning capabilities. Nature **569**(7755), 208–214 (2019). 10.1038/s41586-019-1157-8 31068721 10.1038/s41586-019-1157-8 PMC6522354

[88] D. Wang, S. Hao, B. Dkhil, B. Tian, C. Duan, Ferroelectric materials for neuroinspired computing applications. Fundam. Res. **4**(5), 1272–1291 (2024). 10.1016/j.fmre.2023.04.013 39431127 10.1016/j.fmre.2023.04.013 PMC11489484

[89] G. Indiveri, B. Linares-Barranco, R. Legenstein, G. Deligeorgis, T. Prodromakis, Integration of nanoscale memristor synapses in neuromorphic computing architectures. Nanotechnology **24**(38), 384010 (2013). 10.1088/0957-4484/24/38/384010 23999381 10.1088/0957-4484/24/38/384010

[90] S. Liu, Z. Wu, Z. He, W. Chen, X. Zhong et al., Low-power perovskite neuromorphic synapse with enhanced photon efficiency for directional motion perception. ACS Appl. Mater. Interfaces **16**(17), 22303–22311 (2024). 10.1021/acsami.4c04398 38626428 10.1021/acsami.4c04398

[91] Y. Wang, L. Yin, W. Huang, Y. Li, S. Huang et al., Optoelectronic synaptic devices for neuromorphic computing. Adv. Intell. Syst. **3**(1), 2000099 (2021). 10.1002/aisy.202000099

[92] N. Ilyas, J. Wang, C. Li, D. Li, H. Fu et al., Nanostructured materials and architectures for advanced optoelectronic synaptic devices. Adv. Funct. Mater. **32**(15), 2110976 (2022). 10.1002/adfm.202110976

[93] Y. Cai, Y. Jiang, C. Sheng, Z. Wu, L. Chen et al., *In-situ* artificial retina with all-in-one reconfigurable photomemristor networks. NPJ Flex. Electron. **7**, 29 (2023). 10.1038/s41528-023-00262-3

[94] P. Yang, H. Xu, X. Luo, S. Yu, Y. Liu et al., Tailoring dynamic synaptic plasticity in FeTFT optoelectronic synapse for associative learning. Adv. Electron. Mater. **11**(7), 2400732 (2025). 10.1002/aelm.202400732

[95] C. Han, X. Han, J. Han, M. He, S. Peng et al., Light-stimulated synaptic transistor with high PPF feature for artificial visual perception system application. Adv. Funct. Mater. **32**(22), 2113053 (2022). 10.1002/adfm.202113053

[96] P. Wang, W. Xue, J. Zeng, W. Ci, Q. Chen et al., Wavelength-selective photodetector and neuromorphic visual sensor utilizing intrinsic defect semiconductor. Adv. Funct. Mater. **34**(46), 2407746 (2024). 10.1002/adfm.202407746

[97] T. Zeng, Z. Zhao, K. Ye, Z. Yu, J. Yan et al., Anisotropic optoelectronic synapses in 2D Nb_2_GeTe_4_ for direction-programmable neuromorphic perception and decision-making. Adv. Mater. (2025). 10.1002/adma.202509686 10.1002/adma.202509686 40906459

[98] K.-W. Yau, R.C. Hardie, Phototransduction motifs and variations. Cell **139**(2), 246–264 (2009). 10.1016/j.cell.2009.09.029 19837030 10.1016/j.cell.2009.09.029 PMC2885920

[99] Z. Liu, Y. Fang, Z. Cai, Y. Liu, Z. Dong et al., Advanced dual-input artificial optical synapse for recognition and generative neural network. Nano Energy **132**, 110347 (2024). 10.1016/j.nanoen.2024.110347

[100] H. Shao, W. Wang, Y. Zhang, B. Gao, C. Jiang et al., Adaptive in-sensor computing for enhanced feature perception and broadband image restoration. Adv. Mater. **37**(6), e2414261 (2025). 10.1002/adma.202414261 39659128 10.1002/adma.202414261

[101] X.-M. Dong, C. Chen, Y.-X. Li, H.-C. Sun, B. Liu et al., Molecular cocrystal strategy for retinamorphic vision with UV–vis–NIR perception and fast recognition. ACS Nano **19**(5), 5718–5726 (2025). 10.1021/acsnano.4c16251 39885738 10.1021/acsnano.4c16251

[102] W. Liu, J. Wang, J. Guo, L. Wang, Z. Gu et al., Efficient carbon-based optoelectronic synapses for dynamic visual recognition. Adv. Sci. **12**(11), 2414319 (2025). 10.1002/advs.202414319 10.1002/advs.202414319 PMC11923932 39840530

[103] Y. Deng, S. Liu, X. Ma, S. Guo, B. Zhai et al., Intrinsic defect-driven synergistic synaptic heterostructures for gate-free neuromorphic phototransistors. Adv. Mater. **36**(19), e2309940 (2024). 10.1002/adma.202309940 38373410 10.1002/adma.202309940

[104] S. Dokos, T. Guo, Computational models of neural retina. In: Encyclopedia of Computational Neuroscience, pp. 912–930. Springer New York (2022). 10.1007/978-1-0716-1006-0_652

[105] G.D. Field, E.J. Chichilnisky, Information processing in the primate retina: circuitry and coding. Annu. Rev. Neurosci. **30**, 1–30 (2007). 10.1146/annurev.neuro.30.051606.094252 17335403 10.1146/annurev.neuro.30.051606.094252

[106] M. Ptito, M. Bleau, J. Bouskila, The retina: a window into the brain. Cells **10**(12), 3269 (2021). 10.3390/cells10123269 34943777 10.3390/cells10123269 PMC8699497

[107] K. Chen, H. Hu, I. Song, H.B. Gobeze, W.-J. Lee et al., Organic optoelectronic synapse based on photon-modulated electrochemical doping. Nat. Photon. **17**(7), 629–637 (2023). 10.1038/s41566-023-01232-x

[108] L. Wang, H. Wang, J. Liu, Y. Wang, H. Shao et al., Negative photoconductivity transistors for visuomorphic computing. Adv. Mater. **36**(38), e2403538 (2024). 10.1002/adma.202403538 39040000 10.1002/adma.202403538

[109] J. Yao, Q. Wang, Y. Zhang, Y. Teng, J. Li et al., Ultra-low power carbon nanotube/porphyrin synaptic arrays for persistent photoconductivity and neuromorphic computing. Nat. Commun. **15**(1), 6147 (2024). 10.1038/s41467-024-50490-y 39034334 10.1038/s41467-024-50490-y PMC11271480

[110] J. Fu, C. Nie, F. Sun, G. Li, H. Shi et al., Bionic visual-audio photodetectors with in-sensor perception and preprocessing. Sci. Adv. **10**(7), eadk8199 (2024). 10.1126/sciadv.adk8199 38363832 10.1126/sciadv.adk8199 PMC10871537

[111] T. Zhang, C. Fan, L. Hu, F. Zhuge, X. Pan et al., A reconfigurable all-optical-controlled synaptic device for neuromorphic computing applications. ACS Nano **18**(25), 16236–16247 (2024). 10.1021/acsnano.4c02278 38868857 10.1021/acsnano.4c02278

[112] Z. Dang, F. Guo, Z. Wang, W. Jie, K. Jin et al., Object motion detection enabled by reconfigurable neuromorphic vision sensor under ferroelectric modulation. ACS Nano **18**(40), 27727–27737 (2024). 10.1021/acsnano.4c10231 39324409 10.1021/acsnano.4c10231

[113] L. Wang, Y. Zhang, Z. Guo, X. Meng, Q. Li et al., High-precision attention mechanism for machine vision enabled by an artificial optoelectronic memristor synapse. Nano Lett. **25**(7), 2716–2724 (2025). 10.1021/acs.nanolett.4c05764 39909731 10.1021/acs.nanolett.4c05764

[114] Z. Liu, Y. Wang, Y. Zhang, S. Sun, T. Zhang et al., Harnessing defects in SnSe film *via* photo-induced doping for fully light-controlled artificial synapse. Adv. Mater. **37**(4), 2410783 (2025). 10.1002/adma.202410783 10.1002/adma.202410783 39648576

[115] Q. Yang, J. Hu, H. Li, Q. Du, S. Feng et al., All-optical modulation photodetectors based on the CdS/graphene/Ge sandwich structures for integrated sensing-computing. Adv. Sci. **12**(11), 2413662 (2025). 10.1002/advs.202413662 10.1002/advs.202413662 PMC11923923 39840929

[116] K. Roy, A. Jaiswal, P. Panda, Towards spike-based machine intelligence with neuromorphic computing. Nature **575**(7784), 607–617 (2019). 10.1038/s41586-019-1677-2 31776490 10.1038/s41586-019-1677-2

[117] Y. Huang, J. Liu, J. Harkin, L. McDaid, Y. Luo, An memristor-based synapse implementation using BCM learning rule. Neurocomputing **423**, 336–342 (2021). 10.1016/j.neucom.2020.10.106

[118] K. Chang, B. Hyun, K. Hong, K. Young, J. Won, Memristive devices based on two-dimensional transition metal chalcogenides for neuromorphic computing. Nano-Micro Lett. **14**(1), 58 (2022). 10.1007/s40820-021-00784-3 10.1007/s40820-021-00784-3 PMC8818077 35122527

[119] Y. Wang, S. Nie, S. Liu, Y. Hu, J. Fu et al., Dual-adaptive heterojunction synaptic transistors for efficient machine vision in harsh lighting conditions. Adv. Mater. **36**(32), 2404160 (2024). 10.1002/adma.202404160 10.1002/adma.202404160 38815276

[120] W.C. Abraham, Metaplasticity: tuning synapses and networks for plasticity. Nat. Rev. Neurosci. **9**(5), 387 (2008). 10.1038/nrn2356 18401345 10.1038/nrn2356

[121] J. Benda, A.V.M. Herz, A universal model for spike-frequency adaptation. Neural Comput. **15**(11), 2523–2564 (2003). 10.1162/089976603322385063 14577853 10.1162/089976603322385063

[122] N. Caporale, Y. Dan, Spike timing-dependent plasticity: a Hebbian learning rule. Annu. Rev. Neurosci. **31**, 25–46 (2008). 10.1146/annurev.neuro.31.060407.125639 18275283 10.1146/annurev.neuro.31.060407.125639

[123] D.E. Feldman, The spike-timing dependence of plasticity. Neuron **75**(4), 556–571 (2012). 10.1016/j.neuron.2012.08.001 22920249 10.1016/j.neuron.2012.08.001 PMC3431193

[124] S.K. Nath, S.K. Das, S.K. Nandi, C. Xi, C.V. Marquez et al., Optically tunable electrical oscillations in oxide-based memristors for neuromorphic computing. Adv. Mater. **36**(25), e2400904 (2024). 10.1002/adma.202400904 38516720 10.1002/adma.202400904

[125] S. Kim, J. Heo, S. Kim, M.-H. Kim, Dual functionality of NbOx memristors for synaptic and neuronal emulations in advanced neuromorphic systems. J. Mater. Chem. C **12**(40), 16294–16308 (2024). 10.1039/D4TC03212H

[126] T. Zhang, M. Hu, M.Z.A. Mia, H. Zhang, W. Mao et al., Self-sensitizable neuromorphic device based on adaptive hydrogen gradient. Matter **7**(5), 1799–1816 (2024). 10.1016/j.matt.2024.03.002

[127] Z. Lv, S. Zhu, Y. Wang, Y. Ren, M. Luo et al., Development of bio-voltage operated humidity-sensory neurons comprising self-assembled peptide memristors. Adv. Mater. **36**(33), e2405145 (2024). 10.1002/adma.202405145 38877385 10.1002/adma.202405145

[128] T. Mei, W. Liu, F. Sun, Y. Chen, G. Xu et al., Bio-inspired two-dimensional nanofluidic ionic transistor for neuromorphic signal processing. Angew. Chem. Int. Ed. **63**(17), e202401477 (2024). 10.1002/anie.202401477 10.1002/anie.202401477 38419469

[129] M. Xu, X. Chen, Y. Guo, Y. Wang, D. Qiu et al., Reconfigurable neuromorphic computing: materials, devices, and integration. Adv. Mater. **35**(51), 2301063 (2023). 10.1002/adma.202301063 10.1002/adma.202301063 37285592

[130] X. Wu, E. Li, Y. Liu, W. Lin, R. Yu et al., Artificial multisensory integration nervous system with haptic and iconic perception behaviors. Nano Energy **85**, 106000 (2021). 10.1016/j.nanoen.2021.106000

[131] M. Lanza, A. Sebastian, W.D. Lu, M. Le Gallo, M.-F. Chang et al., Memristive technologies for data storage, computation, encryption, and radio-frequency communication. Science **376**(6597), eabj9979 (2022). 10.1126/science.abj9979 35653464 10.1126/science.abj9979

[132] K. Wang, Y. Jia, X. Yan, A biomimetic afferent nervous system based on the flexible artificial synapse. Nano Energy **100**, 107486 (2022). 10.1016/j.nanoen.2022.107486

[133] J. Ko, C. Ock, H. Gim, K. Hong, Y. Lee et al., Two-dimensional materials for artificial sensory devices: advancing neuromorphic sensing technology. npj 2D Mater. Appl. **9**, 35 (2025). 10.1038/s41699-025-00556-2

[134] H.N. Mohanty, T. Tsuruoka, J.R. Mohanty, K. Terabe, Proton-gated synaptic transistors, based on an electron-beam patterned nafion electrolyte. ACS Appl. Mater. Interfaces **15**(15), 19279–19289 (2023). 10.1021/acsami.3c00756 37023114 10.1021/acsami.3c00756

[135] Y. Chu, H. Tan, C. Zhao, X. Wu, S.-J. Ding, Power-efficient gas-sensing and synaptic diodes based on lateral pentacene/a-IGZO PN junctions. ACS Appl. Mater. Interfaces **14**(7), 9368–9376 (2022). 10.1021/acsami.1c19771 35147029 10.1021/acsami.1c19771

[136] L. Dong, B. Xue, G. Wei, S. Yuan, M. Chen et al., Highly promising 2D/1D BP-C/CNT bionic opto-olfactory co-sensory artificial synapses for multisensory integration. Adv. Sci. **11**(29), 2403665 (2024). 10.1002/advs.202403665 10.1002/advs.202403665 PMC11304314 38828870

[137] H. Jang, S. Ju, S. Lee, J. Choi, U. Byun et al., Recent advances in optoelectronic synaptic devices for neuromorphic computing. Biomimetics **10**(9), 584 (2025). 10.3390/biomimetics10090584 41002818 10.3390/biomimetics10090584 PMC12467574

[138] F. Zhang, C. Li, Z. Li, L. Dong, J. Zhao, Recent progress in three-terminal artificial synapses based on 2D materials: from mechanisms to applications. Microsyst. Nanoeng. **9**, 16 (2023). 10.1038/s41378-023-00487-2 36817330 10.1038/s41378-023-00487-2 PMC9935897

[139] M.-K. Song, S.D. Namgung, D. Choi, H. Kim, H. Seo et al., Proton-enabled activation of peptide materials for biological bimodal memory. Nat. Commun. **11**(1), 5896 (2020). 10.1038/s41467-020-19750-5 33214548 10.1038/s41467-020-19750-5 PMC7677316

[140] H. Ma, H. Fang, X. Xie, Y. Liu, H. Tian et al., Optoelectronic synapses based on MXene/violet phosphorus van der Waals heterojunctions for visual-olfactory crossmodal perception. Nano-Micro Lett. **16**(1), 104 (2024). 10.1007/s40820-024-01330-7 10.1007/s40820-024-01330-7 PMC10834395 38300424

[141] J. Lao, C. Jiang, C. Luo, N. Zhong, B. Tian et al., Self-powered and humidity-modulable optoelectronic synapse. Adv. Mater. Technol. **8**(11), 2201779 (2023). 10.1002/admt.202201779

[142] D. Tan, Z. Zhang, H. Shi, N. Sun, Q. Li et al., Bioinspired artificial visual-respiratory synapse as multimodal scene recognition system with oxidized-vacancies MXene. Adv. Mater. **36**(36), 2407751 (2024). 10.1002/adma.202407751 10.1002/adma.202407751 39011791

[143] Y. Yin, T. Sun, L. Wang, L. Li, P. Guo et al., In-sensor organic electrochemical transistor for the multimode neuromorphic olfactory system. ACS Sens. **9**(8), 4277–4285 (2024). 10.1021/acssensors.4c01423 39099107 10.1021/acssensors.4c01423

[144] T. Jiang, Y. Wang, Y. Zheng, L. Wang, X. He et al., Tetrachromatic vision-inspired neuromorphic sensors with ultraweak ultraviolet detection. Nat. Commun. **14**(1), 2281 (2023). 10.1038/s41467-023-37973-0 37085540 10.1038/s41467-023-37973-0 PMC10121588

[145] S. Dai, X. Liu, Y. Liu, Y. Xu, J. Zhang et al., Emerging iontronic neural devices for neuromorphic sensory computing. Adv. Mater. **35**(39), e2300329 (2023). 10.1002/adma.202300329 36891745 10.1002/adma.202300329

[146] C. Wang, X. Xu, X. Pi, M.D. Butala, W. Huang et al., Neuromorphic device based on silicon nanosheets. Nat. Commun. **13**, 5216 (2022). 10.1038/s41467-022-32884-y 36064545 10.1038/s41467-022-32884-y PMC9445003

[147] M. Wang, D. Ouyang, Y. Dai, D. Huo, W. He et al., 2D piezo-Ferro-opto-electronic artificial synapse for bio-inspired multimodal sensory integration. Adv. Mater. **37**(24), e2500049 (2025). 10.1002/adma.202500049 40190081 10.1002/adma.202500049

[148] F. Nie, H. Fang, J. Wang, L. Zhao, C. Jia et al., An adaptive solid-state synapse with bi-directional relaxation for multimodal recognition and spatio-temporal learning. Adv. Mater. **37**(17), 2412006 (2025). 10.1002/adma.202412006 10.1002/adma.202412006 40091421

[149] W. Zhao, Z. Lin, L. Zhang, X. Lin, J. Wang et al., Bioinspired three-mode photosensitive synaptic LED for optical information processing. Nano Lett. **24**(44), 14109–14117 (2024). 10.1021/acs.nanolett.4c04444 39466915 10.1021/acs.nanolett.4c04444

[150] G. Wu, X. Zhang, G. Feng, J. Wang, K. Zhou et al., Ferroelectric-defined reconfigurable homojunctions for in-memory sensing and computing. Nat. Mater. **22**(12), 1499–1506 (2023). 10.1038/s41563-023-01676-0 37770677 10.1038/s41563-023-01676-0

[151] X. Zhang, D. Liu, J. Wu, E. Cheng, C. Qin et al., Pixel-level hardware strategy for large-scale convolution calculation in neuromorphic devices. Adv. Funct. Mater. **35**(17), 2420045 (2025). 10.1002/adfm.202420045

[152] H. Fang, S. Ma, J. Wang, L. Zhao, F. Nie et al., Multimodal in-sensor computing implemented by easily-fabricated oxide-heterojunction optoelectronic synapses. Adv. Funct. Mater. **34**(49), 2409045 (2024). 10.1002/adfm.202409045

[153] J. Sun, Q. Chen, F. Fan, Z. Zhang, T. Han et al., A dual-mode organic memristor for coordinated visual perceptive computing. Fundam. Res. **4**(6), 1666–1673 (2022). 10.1016/j.fmre.2022.06.022 39734520 10.1016/j.fmre.2022.06.022 PMC11670689

[154] C. Yang, H. Wang, G. Zhou, S. Qin, W. Hou et al., A multifunctional memristor with coexistence of NDR and RS behaviors for logic operation and somatosensory temperature sensing applications. Nano Today **57**, 102382 (2024). 10.1016/j.nantod.2024.102382

[155] S. Zhou, H. Fan, S. Wen, Y. Wei, H. Chen et al., Dual-mode photodetectors mimicking retinal rod and cone cells for high dynamic range image sensor. Laser Photon. Rev. **19**(12), 2402192 (2025). 10.1002/lpor.202402192

[156] Q. He, H. Wang, Y. Zhang, A. Chen, Y. Fu et al., Two-dimensional materials based two-transistor-two-resistor synaptic kernel for efficient neuromorphic computing. Nat. Commun. **16**(1), 4340 (2025). 10.1038/s41467-025-59815-x 40346103 10.1038/s41467-025-59815-x PMC12064777

[157] K. Young, K. Eun, K. Sung, C. Yeop, K. Soh et al., Artificial sensory system based on memristive devices. Exploration **4**(1), 20220162 (2024). 10.1002/EXP.20220162 38854486 10.1002/EXP.20220162 PMC10867403

[158] M. Park, J.Y. Yang, M.J. Yeom, B. Bae, Y. Baek et al., An artificial neuromuscular junction for enhanced reflexes and oculomotor dynamics based on a ferroelectric CuInP_2_S_6_/GaN HEMT. Sci. Adv. **9**(38), eadh9889 (2023). 10.1126/sciadv.adh9889 37738348 10.1126/sciadv.adh9889 PMC10516496

[159] X. Shan, Z. Wang, J. Xie, J. Han, Y. Tao et al., Hemispherical retina emulated by plasmonic optoelectronic memristors with all-optical modulation for neuromorphic stereo vision. Adv. Sci. **11**(36), 2405160 (2024). 10.1002/advs.202405160 10.1002/advs.202405160 PMC11423217 39049682

[160] Y. Ma, M. Chen, F. Aguirre, Y. Yan, S. Pazos et al., Van der Waals engineering of one-transistor-one-ferroelectric-memristor architecture for an energy-efficient neuromorphic array. Nano Lett. **25**(6), 2528–2537 (2025). 10.1021/acs.nanolett.4c06118 39898965 10.1021/acs.nanolett.4c06118 PMC11827105

[161] L. Chen, M. Ren, J. Zhou, X. Zhou, F. Liu et al., Bioinspired iontronic synapse fibers for ultralow-power multiplexing neuromorphic sensorimotor textiles. Proc. Natl. Acad. Sci. U. S. A. **121**(33), e2407971121 (2024). 10.1073/pnas.2407971121 39110725 10.1073/pnas.2407971121 PMC11331142

[162] M.J. Rasch, C. Mackin, M. Le Gallo, A. Chen, A. Fasoli et al., Hardware-aware training for large-scale and diverse deep learning inference workloads using in-memory computing-based accelerators. Nat. Commun. **14**, 5282 (2023). 10.1038/s41467-023-40770-4 37648721 10.1038/s41467-023-40770-4 PMC10469175

[163] Y. Cho, J. Heo, S. Kim, S. Kim, Stacked NbOx-based selector and ZrOx-based resistive memory for high-density crossbar array applications. Surf. Interfaces **41**, 103273 (2023). 10.1016/j.surfin.2023.103273

[164] S. Jain, S. Li, H. Zheng, L. Li, X. Fong et al., Heterogeneous integration of 2D memristor arrays and silicon selectors for compute-in-memory hardware in convolutional neural networks. Nat. Commun. **16**, 2719 (2025). 10.1038/s41467-025-58039-3 40108150 10.1038/s41467-025-58039-3 PMC11923061

[165] L. Shi, G. Zheng, B. Tian, B. Dkhil, C. Duan, Research progress on solutions to the sneak path issue in memristor crossbar arrays. Nanoscale Adv. **2**(5), 1811–1827 (2020). 10.1039/d0na00100g 36132530 10.1039/d0na00100g PMC9418872

[166] Q. Li, S. Wang, Z. Li, X. Hu, Y. Liu et al., High-performance ferroelectric field-effect transistors with ultra-thin indium tin oxide channels for flexible and transparent electronics. Nat. Commun. **15**, 2686 (2024). 10.1038/s41467-024-46878-5 38538586 10.1038/s41467-024-46878-5 PMC10973520

[167] C.-Y. Wei, K.-C. Liao, Y.-J. Yao, C.-E. Wu, C.-L. Chen et al., High-***κ*** HfO_2_/ZrO_2_ superlattice for BEOL-compatible GAAFET memory device. Appl. Phys. Lett. **126**(24), 242902 (2025). 10.1063/5.0274127

[168] F. Kiani, J. Yin, Z. Wang, J.J. Yang, Q. Xia, A fully hardware-based memristive multilayer neural network. Sci. Adv. **7**(48), eabj4801 (2021). 10.1126/sciadv.abj4801 34818038 10.1126/sciadv.abj4801 PMC11559551

[169] Y. Li, K.-W. Ang, Hardware implementation of neuromorphic computing using large-scale memristor crossbar arrays. Adv. Intell. Syst. **3**(1), 2000137 (2021). 10.1002/aisy.202000137

[170] J. Meng, T. Wang, H. Zhu, L. Ji, W. Bao et al., Integrated in-sensor computing optoelectronic device for environment-adaptable artificial retina perception application. Nano Lett. **22**(1), 81–89 (2022). 10.1021/acs.nanolett.1c03240 34962129 10.1021/acs.nanolett.1c03240

[171] S.W. Cho, S.M. Kwon, Y.-H. Kim, S.K. Park, Recent progress in transistor-based optoelectronic synapses: from neuromorphic computing to artificial sensory system. Adv. Intell. Syst. **3**(6), 2000162 (2021). 10.1002/aisy.202000162

[172] G. Lee, J.-H. Baek, F. Ren, S.J. Pearton, G.-H. Lee et al., Artificial neuron and synapse devices based on 2D materials. Small **17**(20), 2100640 (2021). 10.1002/smll.202100640 10.1002/smll.202100640 33817985

[173] S. Wang, C.-Y. Wang, P. Wang, C. Wang, Z.-A. Li et al., Networking retinomorphic sensor with memristive crossbar for brain-inspired visual perception. Natl. Sci. Rev. **8**(2), nwaa172 (2020). 10.1093/nsr/nwaa172 34691573 10.1093/nsr/nwaa172 PMC8288371

[174] F. Zhang, C. Li, Z. Chen, H. Tan, Z. Li et al., Large-scale high uniform optoelectronic synapses array for artificial visual neural network. Microsyst. Nanoeng. **11**(1), 5 (2025). 10.1038/s41378-024-00859-2 39805819 10.1038/s41378-024-00859-2 PMC11731047

[175] X. Li, L. Yi, X. Yin, J. Cheng, Q. Xin et al., Fully screen-printed paper-based ZnO synaptic transistor arrays for visual perception and neuromorphic computing. npj Flex. Electron. **9**, 57 (2025). 10.1038/s41528-025-00425-4

[176] J. Lee, J. Lee, H. Bang, T.W. Yoon, J.H. Ko et al., One-shot remote integration of macromolecular synaptic elements on a chip for ultrathin flexible neural network system. Adv. Mater. **37**(26), 2402361 (2025). 10.1002/adma.202402361 38762775 10.1002/adma.202402361 PMC12232246

[177] D. Joksas, A. AlMutairi, O. Lee, M. Cubukcu, A. Lombardo et al., Memristive, spintronic, and 2D-materials-based devices to improve and complement computing hardware. Adv. Intell. Syst. **4**(8), 2200068 (2022). 10.1002/aisy.202200068

[178] T. Li, J. Miao, X. Fu, B. Song, B. Cai et al., Reconfigurable, non-volatile neuromorphic photovoltaics. Nat. Nanotechnol. **18**(11), 1303–1310 (2023). 10.1038/s41565-023-01446-8 37474683 10.1038/s41565-023-01446-8

[179] L. Sun, Z. Wang, J. Jiang, Y. Kim, B. Joo et al., In-sensor reservoir computing for language learning *via* two-dimensional memristors. Sci. Adv. **7**(20), eabg1455 (2021). 10.1126/sciadv.abg1455 33990331 10.1126/sciadv.abg1455 PMC8121431

[180] Y. Xu, X. Xu, Y. Huang, Y. Tian, M. Cheng et al., Gate-tunable positive and negative photoconductance in near-infrared organic heterostructures for in-sensor computing. Adv. Mater. **36**(30), 2470241 (2024). 10.1002/adma.202470241 10.1002/adma.202402903 38710094

[181] X. Liu, S. Dai, W. Zhao, J. Zhang, Z. Guo et al., All-photolithography fabrication of ion-gated flexible organic transistor array for multimode neuromorphic computing. Adv. Mater. **36**(21), 2312473 (2024). 10.1002/adma.202312473 10.1002/adma.202312473 38385598

[182] Q. Duan, T. Zhang, C. Liu, R. Yuan, G. Li et al., Artificial multisensory neurons with fused haptic and temperature perception for multimodal in-sensor computing. Adv. Intell. Syst. **4**(8), 2270039 (2022). 10.1002/aisy.202270039

[183] A. Bag, G. Ghosh, M.J. Sultan, H.H. Chouhdry, S.J. Hong et al., Bio-inspired sensory receptors for artificial-intelligence perception. Adv. Mater. **37**(26), 2403150 (2025). 10.1002/adma.202403150 10.1002/adma.202403150 38699932

[184] M.S. Kim, M.S. Kim, G.J. Lee, S.-H. Sunwoo, S. Chang et al., Bio-inspired artificial vision and neuromorphic image processing devices. Adv. Mater. Technol. **7**(2), 2100144 (2022). 10.1002/admt.202100144

[185] J.-L. Meng, T.-Y. Wang, L. Chen, Q.-Q. Sun, H. Zhu et al., Energy-efficient flexible photoelectric device with 2D/0D hybrid structure for bio-inspired artificial heterosynapse application. Nano Energy **83**, 105815 (2021). 10.1016/j.nanoen.2021.105815

[186] S. Talanti, K. Fu, X. Zheng, Y. Shi, Y. Tan et al., CMOS-integrated organic neuromorphic imagers for high-resolution dual-modal imaging. Nat. Commun. **16**(1), 4311 (2025). 10.1038/s41467-025-59446-2 40340983 10.1038/s41467-025-59446-2 PMC12062424

[187] J. He, R. Wei, S. Ge, W. Wu, J. Guo et al., Artificial visual-tactile perception array for enhanced memory and neuromorphic computations. InfoMat **6**(3), e12493 (2024). 10.1002/inf2.12493

[188] X. Wu, S. Shi, J. Jiang, D. Lin, J. Song et al., Bionic olfactory neuron with in-sensor reservoir computing for intelligent gas recognition. Adv. Mater. **37**(13), 2419159 (2025). 10.1002/adma.202419159 10.1002/adma.202419159 39945055

[189] J. Guo, F. Guo, H. Zhao, H. Yang, X. Du et al., In-sensor computing with visual-tactile perception enabled by mechano-optical artificial synapse. Adv. Mater. **37**(14), e2419405 (2025). 10.1002/adma.202419405 39998263 10.1002/adma.202419405

[190] H. So, H. Ji, S. Kim, S. Kim, Sophisticated conductance control and multiple synapse functions in TiO_2_-based multistack-layer crossbar array memristor for high-performance neuromorphic systems. Adv. Funct. Mater. **34**(51), 2405544 (2024). 10.1002/adfm.202405544

[191] H. Li, S. Wang, X. Zhang, W. Wang, R. Yang et al., Memristive crossbar arrays for storage and computing applications. Adv. Intell. Syst. **3**(9), 2100017 (2021). 10.1002/aisy.202100017

[192] J. Huang, S. Yang, X. Tang, L. Yang, W. Chen et al., Flexible, transparent, and wafer-scale artificial synapse array based on TiO_x_/Ti_3_C_2_T_x_ film for neuromorphic computing. Adv. Mater. **35**(33), e2303737 (2023). 10.1002/adma.202303737 37339620 10.1002/adma.202303737

[193] E. Li, X. Wu, Q. Chen, S. Wu, L. He et al., Nanoscale channel organic ferroelectric synaptic transistor array for high recognition accuracy neuromorphic computing. Nano Energy **85**, 106010 (2021). 10.1016/j.nanoen.2021.106010

[194] X. Wang, C. Chen, L. Zhu, K. Shi, B. Peng et al., Vertically integrated spiking cone photoreceptor arrays for color perception. Nat. Commun. **14**(1), 3444 (2023). 10.1038/s41467-023-39143-8 37301894 10.1038/s41467-023-39143-8 PMC10257685

[195] T. Lu, J. Xue, P. Shen, H. Liu, X. Gao et al., Two-dimensional fully ferroelectric-gated hybrid computing-in-memory hardware for high-precision and energy-efficient dynamic tracking. Sci. Adv. **10**(36), eadp0174 (2024). 10.1126/sciadv.adp0174 39231224 10.1126/sciadv.adp0174 PMC11373588

[196] H. Kim, S. Oh, H. Choo, D.-H. Kang, J.-H. Park, Tactile neuromorphic system: convergence of triboelectric polymer sensor and ferroelectric polymer synapse. ACS Nano **17**(17), 17332–17341 (2023). 10.1021/acsnano.3c05337 37611149 10.1021/acsnano.3c05337

[197] W. Huang, X. Xia, C. Zhu, P. Steichen, W. Quan et al., Memristive artificial synapses for neuromorphic computing. Nano-Micro Lett. **13**(1), 85 (2021). 10.1007/s40820-021-00618-2 10.1007/s40820-021-00618-2 PMC8006524 34138298

[198] Q. Chen, R. Yang, D. Hu, H. Lin, J. Shi et al., All-optically controlled artificial synaptic device for neural behavior simulation and computer vision. Mater. Today **89**, 107–117 (2025). 10.1016/j.mattod.2025.07.029

[199] S.-O. Park, H. Jeong, S. Seo, Y. Kwon, J. Lee et al., Experimental demonstration of third-order memristor-based artificial sensory nervous system for neuro-inspired robotics. Nat. Commun. **16**(1), 5754 (2025). 10.1038/s41467-025-60818-x 40593563 10.1038/s41467-025-60818-x PMC12215477

[200] F. Zhou, Y. Chai, Near-sensor and in-sensor computing. Nat. Electron. **3**(11), 664–671 (2020). 10.1038/s41928-020-00501-9

[201] B. Dang, T. Zhang, X. Wu, K. Liu, R. Huang et al., Reconfigurable in-sensor processing based on a multi-phototransistor–one-memristor array. Nat. Electron. **7**(11), 991–1003 (2024). 10.1038/s41928-024-01280-3

